# Supplementary material for: The Activity of KIF14, Mieap, and EZR in a New Type of the Invasive Component, Torpedo-Like Structures, Predetermines the Metastatic Potential of Breast Cancer
Source: Cancers (Basel). 2020 Jul 15;12(7):1909. doi: 10.3390/cancers12071909 (PMC7409151; doi:10.3390/cancers12071909)
Supplement: Supplementary file 1 [file cancers-12-01909-s001.pdf]

**Table S1.** Differentially expressed genes in solid and trabecular structures compared to normal breast epithelia (p-value < 0.1)

| Gene | Solid structures | | Trabecular structures | |
|---|---|---|---|---|
| | LogFC | P-value | LogFC | P-value |
| *AACS* | 2.791 | 0.038 | 3.038 | 0.026 |
| *AAGAB* | 3.734 | 0.032 | 3.746 | 0.032 |
| *ABCA12* | 5.171 | 0.001 | 5.070 | 0.001 |
| *ABCC11* | 5.805 | 0.003 | 3.742 | 0.039 |
| *ABCF2* | 3.674 | 0.008 | 2.260 | 0.079 |
| *ABCG2* | -2.135 | 0.038 | -2.386 | 0.023 |
| *ABHD16A* | -2.331 | 0.025 | -3.410 | 0.002 |
| *ABLIM1* | -1.969 | 0.035 | -2.498 | 0.010 |
| *ABO* | -1.108 | 0.097 | -1.139 | 0.088 |
| *ACAA1* | -1.865 | 0.021 | -2.303 | 0.006 |
| *ACOX2* | 2.906 | 0.032 | 3.317 | 0.017 |
| *ACPT* | 1.542 | 0.064 | 1.914 | 0.025 |
| *ACTN4* | 2.217 | 0.091 | 3.592 | 0.010 |
| *ACTR1B* | -1.339 | 0.063 | -1.476 | 0.042 |
| *ACTR6* | 1.723 | 0.032 | 1.515 | 0.055 |
| *ADAM15* | 1.370 | 0.040 | 1.268 | 0.055 |
| *ADAMTS16* | 1.968 | 0.030 | 1.849 | 0.040 |
| *ADAMTS19* | -1.411 | 0.059 | -1.583 | 0.037 |
| *ADAMTS4* | 3.073 | 0.029 | 2.552 | 0.065 |
| *ADAMTS5* | -3.518 | 0.008 | -3.434 | 0.009 |
| *ADCK4* | 2.323 | 0.051 | 3.843 | 0.003 |
| *ADNP2* | 3.725 | 0.003 | 3.363 | 0.006 |
| *ADRB2* | -4.990 | 0.003 | -3.719 | 0.021 |
| *AEBP1* | 2.408 | 0.042 | 3.618 | 0.004 |
| *AF111851* | -1.967 | 0.008 | -2.339 | 0.002 |
| *AGPAT4* | -3.783 | 0.047 | -4.097 | 0.033 |
| *AGT* | 2.392 | 0.041 | 3.241 | 0.008 |
| *AIMP2* | 3.261 | 0.012 | 2.729 | 0.031 |
| *AK027667* | -2.933 | 0.012 | -2.304 | 0.041 |
| *AK075182* | -1.420 | 0.029 | -1.228 | 0.055 |
| *AK090485* | -1.478 | 0.063 | -1.805 | 0.027 |
| *AK124642* | -2.601 | 0.051 | -3.026 | 0.026 |
| *AK130224* | -1.889 | 0.039 | -1.908 | 0.037 |
| *AK5* | -3.467 | 0.002 | -3.213 | 0.003 |
| *AKT1* | 2.988 | 0.070 | 3.147 | 0.058 |
| *ALCAM* | 2.860 | 0.042 | 2.483 | 0.073 |
| *ALDH7A1* | -2.031 | 0.035 | -2.932 | 0.004 |
| *ALG11* | 2.324 | 0.058 | 3.300 | 0.010 |
| *ALG5* | 2.529 | 0.008 | 2.014 | 0.029 |
| *ALKBH6* | 4.223 | 0.001 | 2.456 | 0.036 |
| *ALYREF* | 2.728 | 0.045 | 3.047 | 0.027 |
| *AMOTL1* | -2.854 | 0.031 | -3.137 | 0.019 |
| *ANKRD29* | -2.397 | 0.009 | -1.864 | 0.036 |
| *ANKRD30A* | 2.178 | 0.017 | 2.420 | 0.009 |
| *ANKRD35* | -2.879 | 0.013 | -2.186 | 0.051 |
| *ANKRD36* | 1.893 | 0.082 | 2.287 | 0.040 |
| *ANKRD65* | -3.559 | 0.002 | -3.411 | 0.002 |
| *ANPEP* | -2.315 | 0.060 | -2.435 | 0.049 |
| *ANXA1* | -2.595 | 0.068 | -2.957 | 0.040 |

| | | | | |
|---|---|---|---|---|
| *ANXA9* | 4.935 | 0.002 | 4.253 | 0.007 |
| *AOC3* | 2.230 | 0.097 | 2.281 | 0.090 |
| *AP1G2* | 2.144 | 0.009 | 2.744 | 0.002 |
| *APC* | 2.402 | 0.055 | 3.118 | 0.016 |
| *APCDD1* | -4.793 | 0.008 | -3.816 | 0.029 |
| *APCDD1L* | -2.371 | 0.090 | -3.416 | 0.019 |
| *APCDD1L-AS1* | -3.901 | 0.000 | -2.502 | 0.005 |
| *APOA1BP* | 3.700 | 0.029 | 3.293 | 0.049 |
| *APOD* | -3.832 | 0.034 | -3.609 | 0.044 |
| *APOL2* | 2.639 | 0.023 | 3.033 | 0.010 |
| *ARF1* | 2.331 | 0.060 | 2.225 | 0.072 |
| *ARHGEF10* | -1.794 | 0.050 | -1.862 | 0.043 |
| *ARHGEF37* | -2.643 | 0.068 | -2.620 | 0.070 |
| *ARHGEF3-AS1* | -1.691 | 0.043 | -1.470 | 0.074 |
| *ARIH1* | -3.272 | 0.009 | -2.070 | 0.080 |
| *ARL4C* | -5.110 | 0.002 | -4.864 | 0.003 |
| *ARL6IP6* | 1.858 | 0.068 | 2.381 | 0.023 |
| *ARMC10* | -1.255 | 0.079 | -2.969 | 0.000 |
| *ARRDC3* | -2.179 | 0.075 | -2.295 | 0.062 |
| *ARRDC4* | -2.008 | 0.075 | -2.027 | 0.073 |
| *ASCC3* | 3.359 | 0.037 | 3.403 | 0.035 |
| *ASPM* | 3.419 | 0.042 | 4.889 | 0.006 |
| *ASPN* | 4.044 | 0.000 | 4.688 | 0.000 |
| *ATAD2* | 3.859 | 0.003 | 5.215 | 0.000 |
| *AURKA* | 3.052 | 0.015 | 4.396 | 0.001 |
| *AW571659* | -2.770 | 0.035 | -2.719 | 0.038 |
| *B4GALT3* | 2.914 | 0.004 | 2.957 | 0.003 |
| *B9D2* | 2.052 | 0.076 | 3.151 | 0.010 |
| *BASP1* | 3.711 | 0.069 | 3.376 | 0.095 |
| *BBOX1* | -3.328 | 0.006 | -2.900 | 0.014 |
| *BC014063* | -1.574 | 0.092 | -2.045 | 0.033 |
| *BC044628* | -1.726 | 0.077 | -1.798 | 0.067 |
| *BCAP31* | 1.459 | 0.035 | 1.350 | 0.048 |
| *BCDIN3D* | 4.293 | 0.001 | 4.833 | 0.001 |
| *BCL9* | 3.353 | 0.020 | 2.373 | 0.086 |
| *BF175071* | -3.108 | 0.037 | -2.829 | 0.056 |
| *BGN* | 2.947 | 0.003 | 4.852 | 0.000 |
| *BLVRA* | 1.521 | 0.018 | 1.241 | 0.047 |
| *BLVRB* | 1.728 | 0.077 | 1.645 | 0.091 |
| *BMS1* | 2.990 | 0.066 | 3.456 | 0.037 |
| *BNC1* | -2.585 | 0.012 | -1.909 | 0.053 |
| *BOP1* | -1.824 | 0.040 | -2.742 | 0.004 |
| *BRMS1L* | -2.203 | 0.035 | -2.056 | 0.048 |
| *BROX* | 3.614 | 0.015 | 3.312 | 0.024 |
| *BST2* | 2.086 | 0.038 | 2.131 | 0.035 |
| *BU963192* | 3.430 | 0.027 | 3.839 | 0.015 |
| *BUB1B* | 2.158 | 0.053 | 2.964 | 0.011 |
| *BVES* | -2.099 | 0.018 | -2.015 | 0.023 |
| *C10orf25* | -1.460 | 0.069 | -1.421 | 0.076 |
| *C11orf49* | 1.892 | 0.048 | 1.576 | 0.093 |
| *C15orf26* | -1.358 | 0.065 | -1.247 | 0.087 |
| *C15orf40* | 2.866 | 0.006 | 2.195 | 0.027 |
| *C15orf52* | -2.964 | 0.033 | -4.544 | 0.002 |
| *C15orf61* | -1.644 | 0.097 | -3.364 | 0.002 |
| *C16orf45* | 3.360 | 0.017 | 3.794 | 0.008 |

| | | | | |
|---|---|---|---|---|
| *C16orf54* | -1.944 | 0.050 | -1.752 | 0.074 |
| *C16orf92* | -1.828 | 0.010 | -1.166 | 0.082 |
| *C17orf100* | -2.857 | 0.015 | -2.335 | 0.041 |
| *C17orf62* | 2.058 | 0.034 | 3.444 | 0.001 |
| *C18orf32* | 1.720 | 0.050 | 1.954 | 0.028 |
| *C19orf18* | -1.623 | 0.061 | -1.651 | 0.057 |
| *C19orf52* | 3.918 | 0.003 | 3.145 | 0.014 |
| *C1orf186* | -2.053 | 0.031 | -2.728 | 0.006 |
| *C1orf43* | 1.124 | 0.079 | 1.193 | 0.064 |
| *C1orf53* | 1.515 | 0.097 | 1.584 | 0.084 |
| *C1orf56* | 1.196 | 0.061 | 1.150 | 0.071 |
| *C1QTNF3* | 1.566 | 0.066 | 1.417 | 0.093 |
| *C20orf197* | -1.587 | 0.075 | -1.609 | 0.072 |
| *C2orf40* | -5.208 | 0.000 | -7.216 | 0.000 |
| *C2orf66* | -1.618 | 0.080 | -1.659 | 0.073 |
| *C2orf82* | -2.304 | 0.004 | -1.777 | 0.021 |
| *C3* | -3.515 | 0.020 | -3.527 | 0.020 |
| *C3orf58* | -2.728 | 0.064 | -3.899 | 0.011 |
| *C5orf60* | -1.469 | 0.066 | -1.899 | 0.021 |
| *C6orf136* | -2.626 | 0.047 | -3.484 | 0.011 |
| *C6orf52* | 2.064 | 0.067 | 2.121 | 0.060 |
| *CA11* | -2.161 | 0.074 | -3.613 | 0.006 |
| *CACNA1C* | -3.116 | 0.060 | -3.084 | 0.063 |
| *CACNA2D1* | -4.364 | 0.006 | -3.503 | 0.022 |
| *CADM3* | -4.466 | 0.001 | -4.300 | 0.002 |
| *CALHM2* | -1.673 | 0.077 | -3.590 | 0.001 |
| *CALM2* | 1.106 | 0.028 | 0.834 | 0.088 |
| *CAPN13* | 2.306 | 0.022 | 3.108 | 0.003 |
| *CARD6* | -2.540 | 0.070 | -2.487 | 0.076 |
| *CAV1* | -3.196 | 0.046 | -3.204 | 0.045 |
| *CAV2* | -2.383 | 0.093 | -3.017 | 0.038 |
| *CCDC117* | 1.772 | 0.015 | 1.176 | 0.091 |
| *CCDC120* | -4.533 | 0.004 | -3.446 | 0.020 |
| *CCDC157* | -1.967 | 0.031 | -1.729 | 0.055 |
| *CCDC158* | -2.466 | 0.045 | -2.720 | 0.029 |
| *CCDC175* | 3.297 | 0.087 | 3.167 | 0.099 |
| *CCDC47* | 3.014 | 0.008 | 2.493 | 0.025 |
| *CCDC8* | -2.114 | 0.054 | -1.850 | 0.088 |
| *CCDC92* | 3.442 | 0.014 | 5.015 | 0.001 |
| *CCL21* | -1.442 | 0.095 | -1.483 | 0.086 |
| *CCL28* | -2.658 | 0.069 | -4.445 | 0.005 |
| *CCL5* | -3.763 | 0.031 | -3.646 | 0.036 |
| *CCNB1* | 2.788 | 0.032 | 2.376 | 0.062 |
| *CCNB2* | 3.460 | 0.001 | 2.746 | 0.007 |
| *CCND1* | 2.013 | 0.039 | 1.722 | 0.072 |
| *CCPG1* | 1.814 | 0.042 | 1.609 | 0.067 |
| *CCT3* | 2.207 | 0.047 | 2.967 | 0.011 |
| *CD109* | 4.012 | 0.008 | 2.585 | 0.068 |
| *CD59* | -2.907 | 0.092 | -4.922 | 0.008 |
| *CD97* | -2.149 | 0.022 | -2.248 | 0.017 |
| *CDCA4* | 2.843 | 0.080 | 4.196 | 0.014 |
| *CDH3* | -1.444 | 0.043 | -3.467 | 0.000 |
| *CDKN2AIPNL* | 2.373 | 0.067 | 2.432 | 0.061 |
| *CDKN3* | 3.255 | 0.014 | 2.676 | 0.039 |
| *CDPF1* | -2.620 | 0.020 | -2.475 | 0.027 |

| | | | | |
|---|---|---|---|---|
| *CDR1* | 2.518 | 0.017 | 2.077 | 0.043 |
| *CDS2* | 3.534 | 0.024 | 2.778 | 0.068 |
| *CENPF* | 5.567 | 0.005 | 4.958 | 0.010 |
| *CENPW* | 2.178 | 0.072 | 2.836 | 0.023 |
| *CEP19* | 2.689 | 0.064 | 2.404 | 0.094 |
| *CEP55* | 4.032 | 0.008 | 2.666 | 0.061 |
| *CEP78* | 1.569 | 0.038 | 1.564 | 0.038 |
| *CEP85L* | -2.333 | 0.053 | -2.307 | 0.055 |
| *CERS4* | -3.547 | 0.001 | -3.409 | 0.001 |
| *CFB* | 1.876 | 0.007 | 2.008 | 0.004 |
| *CFP* | 2.257 | 0.080 | 2.834 | 0.032 |
| *CHCHD1* | 2.692 | 0.023 | 1.925 | 0.091 |
| *CHEK1* | 3.462 | 0.012 | 2.165 | 0.095 |
| *CHN1* | 3.080 | 0.033 | 2.376 | 0.090 |
| *CHST2* | -2.771 | 0.011 | -2.788 | 0.011 |
| *CHST9* | -2.381 | 0.010 | -3.160 | 0.001 |
| *CIC* | -1.908 | 0.074 | -2.387 | 0.029 |
| *CKS2* | 2.635 | 0.006 | 2.854 | 0.004 |
| *CLDN11* | -3.226 | 0.034 | -4.989 | 0.002 |
| *CLDN8* | -3.129 | 0.029 | -2.314 | 0.095 |
| *CLEC19A* | 1.218 | 0.071 | 1.814 | 0.011 |
| *CLGN* | 2.909 | 0.021 | 3.889 | 0.004 |
| *CLPSL2* | 4.408 | 0.006 | 2.842 | 0.061 |
| *CLSTN3* | 1.498 | 0.098 | 1.896 | 0.041 |
| *CLU* | -1.795 | 0.030 | -2.051 | 0.015 |
| *CLUAP1* | 2.909 | 0.022 | 3.510 | 0.008 |
| *CMA1* | -2.082 | 0.025 | -1.580 | 0.078 |
| *CMPK2* | 2.827 | 0.031 | 2.290 | 0.073 |
| *CMTM4* | -2.739 | 0.048 | -2.247 | 0.098 |
| *CNEP1R1* | 2.404 | 0.099 | 2.841 | 0.055 |
| *CNIH4* | 1.758 | 0.015 | 1.384 | 0.048 |
| *CNST* | 3.014 | 0.041 | 3.596 | 0.017 |
| *CNTNAP3B* | -2.028 | 0.019 | -2.548 | 0.005 |
| *CNTNAP5* | -2.424 | 0.062 | -2.650 | 0.043 |
| *COL10A1* | 4.653 | 0.001 | 6.730 | 0.000 |
| *COL12A1* | 2.971 | 0.012 | 5.315 | 0.000 |
| *COL17A1* | -3.030 | 0.010 | -4.892 | 0.000 |
| *COL1A1* | 4.744 | 0.000 | 5.638 | 0.000 |
| *COL1A2* | 3.501 | 0.004 | 5.086 | 0.000 |
| *COL3A1* | 5.485 | 0.000 | 6.466 | 0.000 |
| *COL5A1* | 3.871 | 0.006 | 4.857 | 0.001 |
| *COL5A2* | 6.433 | 0.000 | 7.054 | 0.000 |
| *COL6A3* | 2.455 | 0.026 | 4.127 | 0.001 |
| *COMP* | 3.793 | 0.005 | 4.511 | 0.002 |
| *COPA* | 3.753 | 0.003 | 4.290 | 0.001 |
| *COPB2* | 1.915 | 0.013 | 1.822 | 0.017 |
| *COQ3* | -1.448 | 0.099 | -2.526 | 0.008 |
| *COX6C* | 2.003 | 0.006 | 1.826 | 0.010 |
| *COX7A1* | -3.167 | 0.059 | -5.240 | 0.004 |
| *CPAMD8* | -3.884 | 0.004 | -3.425 | 0.009 |
| *CPS1* | -3.726 | 0.007 | -4.821 | 0.001 |
| *CR737597* | -2.014 | 0.017 | -1.480 | 0.067 |
| *CRABP2* | 2.556 | 0.048 | 2.974 | 0.024 |
| *CRAT* | 3.383 | 0.000 | 3.348 | 0.000 |
| *CREB3L1* | 3.051 | 0.058 | 4.144 | 0.014 |

| | | | | |
|---|---|---|---|---|
| *CRIP1* | 3.736 | 0.000 | 3.832 | 0.000 |
| *CRIP2* | 1.393 | 0.079 | 1.848 | 0.024 |
| *CRYAB* | -4.198 | 0.015 | -8.426 | 0.000 |
| *CS* | -1.205 | 0.083 | -2.170 | 0.004 |
| *CSN3* | -4.110 | 0.011 | -3.130 | 0.044 |
| *CSRP1* | -2.375 | 0.004 | -2.877 | 0.001 |
| *CST6* | 3.001 | 0.048 | 2.501 | 0.093 |
| *CST9* | -2.570 | 0.048 | -3.134 | 0.019 |
| *CSTA* | -3.077 | 0.013 | -3.349 | 0.008 |
| *CTDSPL* | -2.126 | 0.018 | -1.834 | 0.038 |
| *CTHRC1* | 2.298 | 0.009 | 4.152 | 0.000 |
| *CTIF* | 2.482 | 0.043 | 2.559 | 0.038 |
| *CTNND1* | -2.277 | 0.020 | -2.538 | 0.011 |
| *CTSD* | 5.419 | 0.002 | 5.184 | 0.002 |
| *CTSS* | -1.783 | 0.029 | -2.096 | 0.013 |
| *CU692621* | -1.068 | 0.048 | -0.891 | 0.092 |
| *CUEDC2* | 1.978 | 0.093 | 2.122 | 0.073 |
| *CXorf21* | -2.517 | 0.080 | -2.826 | 0.052 |
| *CXXC5* | 1.899 | 0.024 | 1.381 | 0.089 |
| *CYP7B1* | -3.453 | 0.002 | -2.511 | 0.016 |
| *CYR61* | -2.964 | 0.036 | -2.596 | 0.062 |
| *CYSLTR2* | -1.712 | 0.037 | -1.378 | 0.085 |
| *CYYR1* | -5.640 | 0.000 | -5.796 | 0.000 |
| *D21S2088E* | -1.920 | 0.055 | -2.267 | 0.026 |
| *DAB2* | -1.589 | 0.091 | -2.123 | 0.029 |
| *DAPL1* | -3.174 | 0.005 | -2.835 | 0.011 |
| *DARS2* | 2.760 | 0.008 | 2.153 | 0.031 |
| *DCDC2* | -2.744 | 0.058 | -4.085 | 0.008 |
| *DDA1* | 3.798 | 0.007 | 4.264 | 0.003 |
| *DDB2* | -1.594 | 0.056 | -1.625 | 0.052 |
| *DDI2* | -3.700 | 0.026 | -3.541 | 0.032 |
| *DDX39A* | 1.690 | 0.093 | 2.680 | 0.012 |
| *DEFA5* | -1.307 | 0.097 | -1.306 | 0.097 |
| *DEGS2* | 3.417 | 0.010 | 3.696 | 0.006 |
| *DENND1B* | 3.202 | 0.026 | 2.757 | 0.051 |
| *DENND6A* | 2.799 | 0.022 | 2.556 | 0.035 |
| *DEPDC1* | 2.795 | 0.042 | 3.886 | 0.007 |
| *DHTKD1* | 2.265 | 0.072 | 2.377 | 0.060 |
| *DLGAP5* | 2.834 | 0.026 | 2.614 | 0.038 |
| *DLL1* | 1.784 | 0.085 | 2.749 | 0.012 |
| *DMD* | -4.011 | 0.002 | -5.133 | 0.000 |
| *DNAH1* | -1.401 | 0.048 | -1.846 | 0.012 |
| *DNAH14* | 2.781 | 0.028 | 4.116 | 0.002 |
| *DNAH5* | 2.506 | 0.073 | 3.104 | 0.030 |
| *DNAJA1* | 2.113 | 0.026 | 1.776 | 0.056 |
| *DNAJC10* | 2.748 | 0.009 | 1.928 | 0.055 |
| *DNAJC22* | 2.954 | 0.008 | 2.224 | 0.037 |
| *DNALI1* | 1.570 | 0.075 | 2.154 | 0.019 |
| *DNM1P46* | -3.170 | 0.021 | -3.453 | 0.013 |
| *DOCK4* | -2.306 | 0.026 | -2.418 | 0.020 |
| *DOLPP1* | 2.833 | 0.067 | 3.258 | 0.038 |
| *DPF1* | 1.736 | 0.058 | 1.560 | 0.086 |
| *DPM1* | 2.872 | 0.022 | 2.531 | 0.040 |
| *DPY19L3* | 2.035 | 0.095 | 3.261 | 0.012 |
| *DQ098707* | -4.080 | 0.008 | -3.781 | 0.012 |

| | | | | |
|---|---|---|---|---|
| *DRD2* | -2.439 | 0.014 | -1.735 | 0.069 |
| *DSN1* | 2.697 | 0.031 | 3.451 | 0.008 |
| *DTX3L* | 1.687 | 0.061 | 1.849 | 0.042 |
| *DUPD1* | -1.614 | 0.041 | -1.286 | 0.097 |
| *DUSP1* | -2.907 | 0.025 | -2.278 | 0.071 |
| *DUSP12* | 2.632 | 0.047 | 2.676 | 0.043 |
| *DUSP2* | -2.162 | 0.049 | -2.068 | 0.058 |
| *DZIP3* | 3.205 | 0.028 | 2.635 | 0.064 |
| *EDN3* | -5.101 | 0.000 | -5.016 | 0.000 |
| *EED* | 1.396 | 0.018 | 1.691 | 0.006 |
| *EFCAB1* | -1.759 | 0.030 | -2.162 | 0.010 |
| *EFHC2* | -1.351 | 0.084 | -1.550 | 0.051 |
| *EGFL7* | 2.144 | 0.035 | 2.691 | 0.011 |
| *EGLN3* | 3.580 | 0.001 | 2.862 | 0.004 |
| *EGR1* | -2.538 | 0.001 | -2.045 | 0.007 |
| *EGR2* | -4.660 | 0.021 | -4.120 | 0.037 |
| *EIF2S2* | 2.248 | 0.047 | 2.126 | 0.058 |
| *EIF3L* | -1.771 | 0.028 | -2.168 | 0.009 |
| *EIF4B* | -1.326 | 0.067 | -1.265 | 0.080 |
| *ELF5* | -2.793 | 0.024 | -3.313 | 0.009 |
| *ELK4* | -1.483 | 0.094 | -1.845 | 0.041 |
| *EMC7* | 0.945 | 0.098 | 1.110 | 0.056 |
| *EMP2* | 1.549 | 0.024 | 1.139 | 0.085 |
| *ENPP1* | 2.363 | 0.072 | 2.716 | 0.041 |
| *ENST00000276974* | -1.900 | 0.004 | -2.336 | 0.001 |
| *ENST00000299512* | -1.620 | 0.025 | -1.898 | 0.011 |
| *ENST00000300992* | -2.453 | 0.014 | -3.711 | 0.001 |
| *ENST00000334163* | 2.033 | 0.025 | 2.236 | 0.015 |
| *ENST00000390312* | -3.808 | 0.011 | -3.077 | 0.034 |
| *ENST00000390323* | -4.153 | 0.065 | -5.225 | 0.024 |
| *ENST00000390399* | -2.126 | 0.010 | -1.843 | 0.022 |
| *ENST00000390426* | -1.730 | 0.043 | -1.841 | 0.032 |
| *ENST00000390539* | -6.486 | 0.000 | -5.151 | 0.000 |
| *ENST00000390547* | -6.552 | 0.001 | -7.055 | 0.001 |
| *ENST00000390606* | -2.273 | 0.059 | -2.220 | 0.065 |
| *ENST00000397595* | -1.753 | 0.089 | -1.777 | 0.085 |
| *ENST00000398190* | -0.909 | 0.094 | -1.210 | 0.031 |
| *ENST00000401715* | -1.280 | 0.019 | -1.219 | 0.025 |
| *ENST00000401937* | -1.344 | 0.058 | -1.251 | 0.076 |
| *ENST00000404609* | -1.602 | 0.009 | -1.377 | 0.021 |
| *ENST00000410078* | -4.740 | 0.030 | -5.896 | 0.009 |
| *ENST00000412855* | 2.206 | 0.053 | 2.133 | 0.060 |
| *ENST00000413663* | -1.547 | 0.082 | -1.640 | 0.066 |
| *ENST00000414915* | 1.285 | 0.083 | 1.422 | 0.057 |
| *ENST00000416350* | -1.464 | 0.072 | -1.802 | 0.030 |
| *ENST00000416507* | -1.551 | 0.051 | -1.466 | 0.063 |
| *ENST00000416519* | 1.871 | 0.074 | 2.017 | 0.056 |
| *ENST00000416593* | -1.467 | 0.085 | -2.071 | 0.019 |
| *ENST00000417039* | -1.882 | 0.069 | -1.974 | 0.058 |
| *ENST00000417510* | -1.992 | 0.018 | -2.909 | 0.001 |
| *ENST00000417823* | -1.628 | 0.096 | -1.986 | 0.047 |
| *ENST00000419614* | -1.738 | 0.014 | -1.251 | 0.066 |
| *ENST00000420096* | -2.888 | 0.023 | -3.053 | 0.017 |
| *ENST00000420273* | -1.527 | 0.041 | -1.331 | 0.071 |
| *ENST00000421481* | -1.670 | 0.042 | -1.997 | 0.017 |

| | | | | |
|---|---|---|---|---|
| *ENST00000421581* | -1.588 | 0.065 | -1.715 | 0.048 |
| *ENST00000422792* | -1.427 | 0.018 | -1.693 | 0.006 |
| *ENST00000424565* | -1.152 | 0.041 | -0.969 | 0.080 |
| *ENST00000424633* | -1.358 | 0.019 | -1.189 | 0.037 |
| *ENST00000424969* | -4.348 | 0.005 | -4.264 | 0.006 |
| *ENST00000425181* | -1.723 | 0.030 | -1.987 | 0.014 |
| *ENST00000425226* | 1.780 | 0.015 | 1.943 | 0.009 |
| *ENST00000427962* | -1.568 | 0.037 | -1.731 | 0.023 |
| *ENST00000429552* | -1.202 | 0.047 | -1.193 | 0.048 |
| *ENST00000430152* | -1.882 | 0.036 | -1.774 | 0.046 |
| *ENST00000430258* | -1.215 | 0.067 | -1.230 | 0.064 |
| *ENST00000431782* | -1.379 | 0.027 | -1.407 | 0.025 |
| *ENST00000432629* | -1.337 | 0.020 | -1.265 | 0.027 |
| *ENST00000433051* | -1.875 | 0.031 | -1.897 | 0.030 |
| *ENST00000434244* | 2.321 | 0.013 | 2.198 | 0.017 |
| *ENST00000434710* | -3.955 | 0.009 | -3.563 | 0.017 |
| *ENST00000436077* | 2.192 | 0.011 | 2.366 | 0.007 |
| *ENST00000439423* | -0.874 | 0.084 | -0.924 | 0.069 |
| *ENST00000441444* | -1.538 | 0.064 | -1.509 | 0.069 |
| *ENST00000441465* | -1.619 | 0.057 | -1.672 | 0.050 |
| *ENST00000442083* | -0.949 | 0.084 | -1.134 | 0.042 |
| *ENST00000442318* | -1.442 | 0.057 | -1.286 | 0.086 |
| *ENST00000443330* | -2.054 | 0.042 | -1.795 | 0.072 |
| *ENST00000450820* | -2.629 | 0.008 | -2.596 | 0.009 |
| *ENST00000451609* | 2.492 | 0.004 | 2.336 | 0.006 |
| *ENST00000452286* | -1.728 | 0.051 | -2.123 | 0.020 |
| *ENST00000452501* | -1.682 | 0.010 | -1.841 | 0.006 |
| *ENST00000453166* | -2.799 | 0.046 | -3.837 | 0.009 |
| *ENST00000453179* | -1.156 | 0.090 | -1.458 | 0.037 |
| *ENST00000453309* | -2.409 | 0.034 | -2.906 | 0.013 |
| *ENST00000453678* | -1.914 | 0.046 | -1.854 | 0.053 |
| *ENST00000455206* | -1.216 | 0.036 | -1.276 | 0.029 |
| *ENST00000456185* | -1.217 | 0.032 | -1.192 | 0.035 |
| *ENST00000456555* | -1.078 | 0.091 | -1.411 | 0.032 |
| *ENST00000457863* | 2.409 | 0.014 | 2.264 | 0.019 |
| *ENST00000458293* | -1.466 | 0.080 | -1.443 | 0.085 |
| *ENST00000461060* | -1.065 | 0.055 | -1.197 | 0.034 |
| *ENST00000461513* | -1.221 | 0.031 | -1.670 | 0.005 |
| *ENST00000462503* | -1.438 | 0.072 | -1.538 | 0.056 |
| *ENST00000462693* | -2.321 | 0.006 | -1.547 | 0.049 |
| *ENST00000464118* | -1.058 | 0.058 | -1.364 | 0.018 |
| *ENST00000466297* | -1.263 | 0.067 | -1.428 | 0.041 |
| *ENST00000469899* | -1.222 | 0.046 | -1.198 | 0.050 |
| *ENST00000471205* | -1.136 | 0.071 | -1.358 | 0.034 |
| *ENST00000476880* | -1.731 | 0.058 | -1.580 | 0.081 |
| *ENST00000481963* | -1.193 | 0.060 | -1.038 | 0.097 |
| *ENST00000483158* | -3.803 | 0.014 | -4.457 | 0.005 |
| *ENST00000488396* | -1.199 | 0.044 | -1.970 | 0.002 |
| *ENST00000492167* | -4.072 | 0.011 | -4.151 | 0.010 |
| *ENST00000494861* | -1.411 | 0.022 | -1.174 | 0.051 |
| *ENST00000496042* | -1.744 | 0.025 | -1.322 | 0.080 |
| *ENST00000496488* | 3.596 | 0.004 | 2.035 | 0.077 |
| *ENST00000497325* | -1.450 | 0.018 | -1.539 | 0.013 |
| *ENST00000502684* | -1.756 | 0.022 | -1.698 | 0.026 |
| *ENST00000503394* | -1.994 | 0.052 | -1.969 | 0.082 |

| | | | | |
|---|---|---|---|---|
| *ENST00000506710* | -2.078 | 0.008 | -1.494 | 0.043 |
| *ENST00000508156* | -2.780 | 0.024 | -2.158 | 0.071 |
| *ENST00000509568* | -1.411 | 0.022 | -1.279 | 0.036 |
| *ENST00000510758* | -1.404 | 0.063 | -1.287 | 0.086 |
| *ENST00000510975* | -1.977 | 0.027 | -2.242 | 0.014 |
| *ENST00000511794* | -1.778 | 0.025 | -1.561 | 0.046 |
| *ENST00000511953* | -2.049 | 0.024 | -1.536 | 0.080 |
| *ENST00000512898* | -2.105 | 0.021 | -1.934 | 0.033 |
| *ENST00000512915* | 1.891 | 0.084 | 2.448 | 0.030 |
| *ENST00000514327* | -1.544 | 0.068 | -1.628 | 0.055 |
| *ENST00000515599* | 3.191 | 0.003 | 3.300 | 0.002 |
| *ENST00000521432* | 2.750 | 0.077 | 3.024 | 0.055 |
| *ENST00000524287* | -1.316 | 0.098 | -1.530 | 0.058 |
| *ENST00000527434* | 2.689 | 0.003 | 2.114 | 0.015 |
| *ENST00000550042* | -2.176 | 0.061 | -2.647 | 0.026 |
| *ENST00000554307* | -1.528 | 0.074 | -1.406 | 0.097 |
| *ENST00000559385* | -0.852 | 0.089 | -0.915 | 0.070 |
| *ENST00000567054* | -1.901 | 0.092 | -2.175 | 0.057 |
| *ENST00000567899* | -1.286 | 0.090 | -1.421 | 0.063 |
| *ENST00000575236* | -1.526 | 0.053 | -1.664 | 0.037 |
| *ENST00000579793* | 2.005 | 0.022 | 1.465 | 0.082 |
| *ENST00000585065* | 2.885 | 0.065 | 3.034 | 0.053 |
| *ENST00000589177* | 3.156 | 0.003 | 3.004 | 0.005 |
| *ENST00000596267* | 2.483 | 0.021 | 2.502 | 0.020 |
| *ENST00000600588* | -1.012 | 0.071 | -1.485 | 0.012 |
| *ENST00000605188* | 1.080 | 0.067 | 1.567 | 0.012 |
| *ENST00000607314* | -5.947 | 0.000 | -6.531 | 0.000 |
| *ENST00000607355* | 2.503 | 0.046 | 2.879 | 0.024 |
| *ENST00000608694* | 3.214 | 0.004 | 2.096 | 0.046 |
| *ENST00000609203* | -1.479 | 0.075 | -1.372 | 0.097 |
| *ENST00000610439* | -3.743 | 0.028 | -5.630 | 0.002 |
| *ENST00000612474* | -3.351 | 0.000 | -2.343 | 0.007 |
| *ENST00000612725* | -1.835 | 0.024 | -1.294 | 0.098 |
| *ENST00000613254* | 2.133 | 0.068 | 2.643 | 0.028 |
| *ENST00000613594* | -3.543 | 0.024 | -4.782 | 0.004 |
| *ENST00000615743* | 1.236 | 0.091 | 1.603 | 0.033 |
| *ENST00000616656* | -1.552 | 0.026 | -1.769 | 0.013 |
| *ENST00000618777* | -1.938 | 0.053 | -1.940 | 0.052 |
| *ENST00000619495* | -2.041 | 0.063 | -1.817 | 0.095 |
| *ENST00000620395* | -3.590 | 0.004 | -3.008 | 0.007 |
| *ENST00000621052* | 2.548 | 0.011 | 2.875 | 0.005 |
| *EPB41* | -2.728 | 0.013 | -2.057 | 0.052 |
| *EPHB1* | -3.636 | 0.000 | -2.528 | 0.000 |
| *EPSTI1* | 2.152 | 0.029 | 1.571 | 0.099 |
| *ERICH5* | -2.708 | 0.000 | -2.529 | 0.001 |
| *ERMAP* | -2.161 | 0.035 | -2.643 | 0.012 |
| *ERMP1* | 3.820 | 0.004 | 3.925 | 0.003 |
| *ERN2* | 2.697 | 0.058 | 3.504 | 0.017 |
| *ESR1* | 3.844 | 0.004 | 2.379 | 0.055 |
| *ETF1* | 1.914 | 0.030 | 1.695 | 0.052 |
| *ETFDH* | 2.367 | 0.048 | 3.473 | 0.006 |
| *ETNK2* | 2.178 | 0.005 | 1.612 | 0.028 |
| *ETS2* | -3.201 | 0.014 | -2.051 | 0.097 |
| *EXOC1* | 1.947 | 0.035 | 3.249 | 0.001 |
| *EZR* | 1.993 | 0.036 | 1.918 | 0.043 |

| | | | | |
|---|---|---|---|---|
| *F11R* | 1.067 | 0.090 | 1.093 | 0.083 |
| *F13A1* | 3.636 | 0.019 | 4.639 | 0.004 |
| *FABP5* | 2.902 | 0.062 | 2.610 | 0.090 |
| *FABP7* | -2.037 | 0.060 | -2.264 | 0.039 |
| *FADD* | 2.325 | 0.045 | 2.038 | 0.075 |
| *FAHD2A* | -2.692 | 0.032 | -2.227 | 0.071 |
| *FAM109A* | -3.613 | 0.015 | -3.259 | 0.026 |
| *FAM114A1* | 3.723 | 0.004 | 2.812 | 0.021 |
| *FAM138A* | -2.195 | 0.018 | -1.467 | 0.097 |
| *FAM160A1* | -2.237 | 0.015 | -3.416 | 0.001 |
| *FAM189B* | 3.821 | 0.002 | 3.570 | 0.003 |
| *FAM212A* | -1.366 | 0.078 | -1.704 | 0.032 |
| *FAM3D* | -6.058 | 0.000 | -5.426 | 0.000 |
| *FAM53B* | -3.044 | 0.026 | -4.832 | 0.001 |
| *FAM53B-AS1* | -1.476 | 0.056 | -1.688 | 0.031 |
| *FAM90A27P* | -1.387 | 0.072 | -1.563 | 0.045 |
| *FANCI* | 2.428 | 0.029 | 2.807 | 0.013 |
| *FAP* | 3.294 | 0.008 | 5.527 | 0.000 |
| *FARSB* | 1.978 | 0.099 | 2.489 | 0.043 |
| *FASN* | 2.399 | 0.001 | 2.138 | 0.003 |
| *FASTKD3* | 4.482 | 0.002 | 3.124 | 0.018 |
| *FBXO28* | 2.370 | 0.015 | 1.881 | 0.047 |
| *FCER1A* | -3.174 | 0.026 | -3.224 | 0.024 |
| *FCGBP* | -3.388 | 0.030 | -3.936 | 0.014 |
| *FCGRT* | -1.864 | 0.047 | -1.576 | 0.088 |
| *FCRL1* | -2.122 | 0.035 | -1.737 | 0.078 |
| *FDCSP* | -3.715 | 0.030 | -3.687 | 0.031 |
| *FEM1C* | 3.637 | 0.001 | 2.419 | 0.012 |
| *FERMT1* | -2.009 | 0.058 | -1.750 | 0.094 |
| *FGF14* | -3.749 | 0.003 | -2.799 | 0.019 |
| *FGF14-AS2* | -3.034 | 0.023 | -2.815 | 0.033 |
| *FGF2* | -3.750 | 0.000 | -4.080 | 0.000 |
| *FGL2* | -2.261 | 0.011 | -2.408 | 0.007 |
| *FIGF* | -2.150 | 0.068 | -2.584 | 0.032 |
| *FIGN* | -2.064 | 0.029 | -2.534 | 0.010 |
| *FKBP4* | 2.858 | 0.017 | 2.811 | 0.018 |
| *FLJ22447* | 2.203 | 0.029 | 1.923 | 0.053 |
| *FLVCR1* | 2.168 | 0.067 | 3.835 | 0.003 |
| *FLVCR2* | -1.997 | 0.045 | -2.031 | 0.042 |
| *FMO2* | -3.179 | 0.022 | -2.328 | 0.083 |
| *FN1* | 6.517 | 0.000 | 7.046 | 0.000 |
| *FOLR1* | -4.694 | 0.021 | -5.393 | 0.009 |
| *FOLR3* | -1.793 | 0.004 | -1.250 | 0.035 |
| *FOSB* | -3.058 | 0.063 | -4.067 | 0.017 |
| *FOXN3* | -3.435 | 0.028 | -2.852 | 0.063 |
| *FRG2* | -1.805 | 0.057 | -1.626 | 0.084 |
| *FSIP1* | 2.645 | 0.038 | 4.276 | 0.002 |
| *FSTL3* | 2.255 | 0.041 | 4.099 | 0.001 |
| *FUT3* | 3.667 | 0.007 | 3.641 | 0.007 |
| *FUT8* | 3.424 | 0.001 | 4.541 | 0.000 |
| *FYB* | -2.850 | 0.013 | -4.170 | 0.001 |
| *FZD7* | -2.923 | 0.039 | -3.386 | 0.019 |
| *G0S2* | -5.023 | 0.000 | -5.655 | 0.000 |
| *GABRG1* | -1.618 | 0.025 | -1.274 | 0.069 |
| *GABRP* | -3.858 | 0.007 | -4.418 | 0.002 |

| | | | | |
|---|---|---|---|---|
| *GALK2* | -2.351 | 0.091 | -3.413 | 0.019 |
| *GALNT8* | -1.142 | 0.053 | -1.154 | 0.051 |
| *GAPDHS* | -1.529 | 0.058 | -1.455 | 0.069 |
| *GAS2L1* | -2.426 | 0.036 | -2.230 | 0.051 |
| *GBA* | 3.187 | 0.018 | 2.308 | 0.075 |
| *GBGT1* | -2.638 | 0.030 | -2.411 | 0.045 |
| *GCNT2* | -1.610 | 0.014 | -1.923 | 0.004 |
| *GFM2* | 2.799 | 0.028 | 2.908 | 0.023 |
| *GGH* | 4.151 | 0.001 | 3.708 | 0.002 |
| *GGTA1P* | -2.347 | 0.022 | -3.211 | 0.003 |
| *GINS1* | 3.975 | 0.000 | 2.658 | 0.004 |
| *GJA1* | 3.344 | 0.033 | 2.683 | 0.080 |
| *GJB2* | 2.889 | 0.056 | 3.167 | 0.038 |
| *GLB1L* | -2.000 | 0.070 | -2.461 | 0.030 |
| *GLG1* | -1.616 | 0.040 | -1.737 | 0.029 |
| *GLIPR2* | -2.693 | 0.088 | -3.124 | 0.051 |
| *GLT8D1* | 2.473 | 0.048 | 2.411 | 0.053 |
| *GLTSCR2* | -1.288 | 0.046 | -1.406 | 0.031 |
| *GLUL* | 3.259 | 0.008 | 3.150 | 0.010 |
| *GMEB1* | 2.893 | 0.034 | 2.903 | 0.033 |
| *GNB2L1* | -1.698 | 0.027 | -1.342 | 0.072 |
| *GPR141* | -1.239 | 0.059 | -1.643 | 0.016 |
| *GPR75* | -2.847 | 0.048 | -4.330 | 0.005 |
| *GPRC5A* | 6.217 | 0.000 | 5.854 | 0.000 |
| *GPRC5B* | -5.398 | 0.002 | -6.709 | 0.000 |
| *GPX8* | 3.758 | 0.001 | 4.538 | 0.000 |
| *GRAMD3* | -2.804 | 0.045 | -2.707 | 0.052 |
| *GRB14* | 6.529 | 0.011 | 4.826 | 0.050 |
| *GRB2* | 4.053 | 0.009 | 2.952 | 0.047 |
| *GRIN3A* | -1.427 | 0.070 | -1.794 | 0.027 |
| *GSAP* | 3.861 | 0.023 | 2.995 | 0.070 |
| *GSTP1* | -2.075 | 0.014 | -2.604 | 0.007 |
| *GTF2A1* | 2.531 | 0.075 | 2.560 | 0.072 |
| *GTF2H2C_2* | 2.515 | 0.011 | 2.293 | 0.019 |
| *GTF3C5* | -1.515 | 0.012 | -1.911 | 0.002 |
| *GUK1* | 1.551 | 0.079 | 1.756 | 0.049 |
| *GUSBP5* | -1.664 | 0.045 | -1.441 | 0.079 |
| *H2AFV* | 1.775 | 0.096 | 2.215 | 0.042 |
| *H2AFY* | 1.882 | 0.004 | 2.414 | 0.000 |
| *HAGH* | 3.679 | 0.004 | 3.107 | 0.012 |
| *HAX1* | 1.422 | 0.036 | 1.274 | 0.058 |
| *HCLS1* | -3.755 | 0.011 | -4.499 | 0.003 |
| *HDAC3* | 2.046 | 0.016 | 1.978 | 0.020 |
| *HDAC5* | -2.477 | 0.075 | -2.468 | 0.076 |
| *HEATR5A* | 2.453 | 0.009 | 1.637 | 0.066 |
| *HEPACAM2* | -2.735 | 0.009 | -2.483 | 0.016 |
| *HHAT* | 2.660 | 0.043 | 3.659 | 0.008 |
| *HIGD2B* | -1.474 | 0.096 | -1.528 | 0.085 |
| *HIST1H1C* | 2.882 | 0.038 | 3.985 | 0.006 |
| *HIST1H2AI* | 2.846 | 0.014 | 2.549 | 0.026 |
| *HIST1H2BC* | 1.082 | 0.082 | 1.290 | 0.042 |
| *HIST1H2BD* | 1.094 | 0.052 | 1.243 | 0.030 |
| *HIST1H2BI* | 1.471 | 0.049 | 1.852 | 0.016 |
| *HIST1H2BJ* | 1.251 | 0.030 | 1.541 | 0.010 |
| *HIST1H2BK* | 0.994 | 0.082 | 1.237 | 0.035 |

| | | | | |
|---|---|---|---|---|
| *HIST1H2BL* | 1.302 | 0.099 | 1.880 | 0.022 |
| *HIST1H2BM* | 1.644 | 0.038 | 2.013 | 0.014 |
| *HIST1H3B* | 2.651 | 0.027 | 2.864 | 0.018 |
| *HIST1H3H* | 1.752 | 0.035 | 2.246 | 0.009 |
| *HIST1H3J* | 2.958 | 0.045 | 2.490 | 0.086 |
| *HIST2H2AA4* | 2.281 | 0.071 | 2.834 | 0.029 |
| *HIST2H2AB* | 1.107 | 0.048 | 1.631 | 0.006 |
| *HIST2H2AC* | 1.291 | 0.069 | 1.387 | 0.053 |
| *HIST2H2BF* | 1.472 | 0.041 | 1.640 | 0.025 |
| *HJURP* | 3.100 | 0.054 | 2.748 | 0.083 |
| *HLF* | -5.713 | 0.006 | -4.342 | 0.028 |
| *HMGB2* | 1.748 | 0.029 | 1.691 | 0.034 |
| *HMGB3* | 5.350 | 0.000 | 4.831 | 0.000 |
| *HMGB3P1* | 2.657 | 0.009 | 2.486 | 0.013 |
| *HMMR* | 4.244 | 0.005 | 3.891 | 0.008 |
| *HN1L* | 1.796 | 0.009 | 1.933 | 0.006 |
| *HOTAIRM1* | -4.547 | 0.000 | -4.162 | 0.000 |
| *HOXA2* | -2.734 | 0.001 | -2.466 | 0.001 |
| *HOXA3* | -4.818 | 0.003 | -4.609 | 0.004 |
| *HOXA7* | -2.130 | 0.047 | -1.933 | 0.069 |
| *HOXC6* | 4.115 | 0.012 | 2.970 | 0.057 |
| *HSD17B3* | -1.281 | 0.075 | -1.527 | 0.037 |
| *HSPA13* | 1.788 | 0.041 | 2.064 | 0.021 |
| *HSPA14* | 3.079 | 0.014 | 2.332 | 0.053 |
| *HSPA4* | 2.495 | 0.033 | 2.290 | 0.048 |
| *HSPA8* | 1.089 | 0.085 | 1.233 | 0.054 |
| *HSPB1* | 1.613 | 0.018 | 2.186 | 0.003 |
| *HULC* | -2.077 | 0.035 | -2.083 | 0.035 |
| *HYDIN* | -2.838 | 0.042 | -3.054 | 0.030 |
| *IARS2* | 1.963 | 0.051 | 1.900 | 0.058 |
| *ID4* | -3.515 | 0.033 | -3.183 | 0.051 |
| *IDS* | 1.964 | 0.092 | 2.519 | 0.035 |
| *IFFO2* | -5.354 | 0.001 | -4.135 | 0.008 |
| *IFI27* | 4.121 | 0.000 | 3.901 | 0.000 |
| *IFI6* | 1.903 | 0.091 | 2.464 | 0.033 |
| *IGFBP5* | 3.532 | 0.002 | 3.354 | 0.003 |
| *IGFBP6* | -2.849 | 0.026 | -2.815 | 0.028 |
| *IGJ* | -6.064 | 0.002 | -5.598 | 0.004 |
| *IGLL1* | -4.961 | 0.014 | -6.183 | 0.003 |
| *IGLL5* | -4.822 | 0.019 | -5.743 | 0.007 |
| *IL10RB-AS1* | -3.571 | 0.006 | -3.745 | 0.004 |
| *IL13RA1* | 1.883 | 0.037 | 2.077 | 0.023 |
| *IL17RD* | -2.874 | 0.073 | -2.851 | 0.075 |
| *IL22RA1* | -1.830 | 0.050 | -1.515 | 0.098 |
| *IMPAD1* | 2.244 | 0.004 | 1.698 | 0.024 |
| *INAFM1* | 3.515 | 0.003 | 4.587 | 0.000 |
| *INAFM2* | -2.447 | 0.078 | -2.784 | 0.048 |
| *INHBA* | 1.685 | 0.036 | 3.173 | 0.001 |
| *INTS6* | 3.364 | 0.009 | 2.971 | 0.018 |
| *IPO13* | 2.522 | 0.064 | 2.343 | 0.083 |
| *IPPK* | 3.776 | 0.017 | 3.360 | 0.031 |
| *IQCA1* | -2.748 | 0.007 | -2.458 | 0.013 |
| *IQSEC3* | -1.683 | 0.047 | -2.189 | 0.013 |
| *IRF3* | 2.358 | 0.045 | 2.426 | 0.039 |
| *IRX1* | -4.662 | 0.001 | -7.259 | 0.000 |

| | | | | |
|---|---|---|---|---|
| *IRX4* | -3.933 | 0.015 | -4.785 | 0.004 |
| *ISM1* | -4.617 | 0.000 | -5.717 | 0.000 |
| *ISY1-RAB43* | -2.462 | 0.021 | -2.836 | 0.009 |
| *ITGAV* | 1.765 | 0.042 | 1.548 | 0.070 |
| *ITGB1BP1* | 2.093 | 0.062 | 2.096 | 0.061 |
| *ITGB3BP* | 2.286 | 0.045 | 2.110 | 0.062 |
| *ITM2A* | -3.462 | 0.001 | -4.521 | 0.000 |
| *ITPA* | -2.466 | 0.023 | -1.728 | 0.097 |
| *IWS1* | 3.510 | 0.028 | 4.465 | 0.007 |
| *JMJD7* | -1.715 | 0.012 | -1.167 | 0.074 |
| *JPH2* | -2.303 | 0.053 | -3.035 | 0.014 |
| *KANK1* | -2.877 | 0.061 | -4.627 | 0.005 |
| *KANK2* | 3.306 | 0.031 | 4.005 | 0.011 |
| *KAZALD1* | 2.383 | 0.088 | 4.134 | 0.006 |
| *KBTBD6* | -2.913 | 0.014 | -2.251 | 0.049 |
| *KCNH8* | -1.672 | 0.006 | -1.847 | 0.003 |
| *KCNIP3* | -1.493 | 0.012 | -1.398 | 0.018 |
| *KCNJ8* | -4.067 | 0.000 | -4.865 | 0.000 |
| *KCNK1* | 2.590 | 0.013 | 2.286 | 0.026 |
| *KCNK15* | 3.100 | 0.004 | 3.223 | 0.003 |
| *KCNK16* | -1.208 | 0.096 | -1.202 | 0.098 |
| *KCNK5* | -3.230 | 0.039 | -2.697 | 0.079 |
| *KCNN4* | -4.298 | 0.006 | -3.610 | 0.017 |
| *KCTD12* | -2.463 | 0.011 | -1.955 | 0.036 |
| *KCTD14* | -4.498 | 0.000 | -4.619 | 0.000 |
| *KDM5B* | 1.658 | 0.059 | 1.955 | 0.029 |
| *KHNYN* | -2.660 | 0.067 | -3.544 | 0.019 |
| *KIAA0101* | 4.516 | 0.001 | 4.293 | 0.001 |
| *KIAA0196* | 1.947 | 0.074 | 2.167 | 0.049 |
| *KIF14* | 3.874 | 0.060 | 3.422 | 0.093 |
| *KIF25* | -2.366 | 0.053 | -2.671 | 0.031 |
| *KIT* | -4.153 | 0.000 | -7.598 | 0.000 |
| *KLHL13* | -2.410 | 0.037 | -3.886 | 0.002 |
| *KLHL14* | 1.386 | 0.034 | 1.870 | 0.006 |
| *KLHL21* | -2.881 | 0.056 | -3.960 | 0.012 |
| *KLHL7* | -1.814 | 0.084 | -3.311 | 0.004 |
| *KLK7* | -2.966 | 0.019 | -2.802 | 0.026 |
| *KLK8* | -2.240 | 0.034 | -4.611 | 0.000 |
| *KLRB1* | -3.087 | 0.006 | -3.111 | 0.006 |
| *KLRC3* | -2.124 | 0.025 | -2.377 | 0.014 |
| *KPNA2* | 1.922 | 0.041 | 2.760 | 0.006 |
| *KPNB1* | 1.769 | 0.032 | 1.760 | 0.033 |
| *KRT13* | -4.652 | 0.000 | -3.281 | 0.006 |
| *KRT14* | -3.254 | 0.011 | -4.327 | 0.002 |
| *KRT15* | -6.310 | 0.001 | -6.971 | 0.000 |
| *KRT17P5* | -1.650 | 0.077 | -1.637 | 0.079 |
| *KRT19* | 1.532 | 0.029 | 1.702 | 0.017 |
| *KRT19P2* | 1.470 | 0.099 | 1.559 | 0.083 |
| *KRT5* | -3.557 | 0.012 | -4.124 | 0.005 |
| *KRT8* | 1.334 | 0.027 | 1.728 | 0.006 |
| *KRT8P12* | 1.483 | 0.022 | 2.016 | 0.003 |
| *KRTAP6-3* | -1.852 | 0.098 | -2.179 | 0.055 |
| *L3HYPDH* | 2.652 | 0.040 | 2.902 | 0.027 |
| *LAGE3* | 1.805 | 0.081 | 1.698 | 0.099 |
| *LAMA3* | -3.169 | 0.089 | -3.579 | 0.057 |

| | | | | |
|---|---|---|---|---|
| *LARP1B* | 3.046 | 0.031 | 3.032 | 0.032 |
| *LARP6* | -1.648 | 0.093 | -1.928 | 0.053 |
| *LAYN* | -2.229 | 0.039 | -2.293 | 0.034 |
| *LBR* | 3.657 | 0.015 | 3.521 | 0.019 |
| *LDHB* | -3.603 | 0.024 | -3.647 | 0.022 |
| *LEF1* | 4.216 | 0.009 | 4.025 | 0.012 |
| *LFNG* | 3.980 | 0.007 | 3.003 | 0.034 |
| *LGALS9* | 3.325 | 0.003 | 2.110 | 0.042 |
| *LGR6* | -2.889 | 0.028 | -4.666 | 0.001 |
| *LHFPL4* | -2.842 | 0.004 | -2.930 | 0.003 |
| *LIMK2* | -3.000 | 0.016 | -5.288 | 0.000 |
| *LIN9* | 4.072 | 0.033 | 4.186 | 0.029 |
| *LINC00086* | -2.084 | 0.053 | -2.864 | 0.011 |
| *LINC00152* | 2.110 | 0.023 | 2.445 | 0.010 |
| *LINC00240* | -2.121 | 0.029 | -2.374 | 0.016 |
| *LINC00298* | 1.541 | 0.086 | 1.830 | 0.045 |
| *LINC00515* | -2.597 | 0.087 | -2.558 | 0.091 |
| *LINC00842* | -2.034 | 0.042 | -2.119 | 0.035 |
| *LINC00865* | -2.723 | 0.018 | -2.708 | 0.019 |
| *LINC01000* | -1.260 | 0.099 | -2.195 | 0.007 |
| *LINC01152* | -3.773 | 0.003 | -3.239 | 0.009 |
| *LINC01235* | -2.532 | 0.028 | -2.998 | 0.011 |
| *LINC01270* | 2.407 | 0.056 | 2.281 | 0.068 |
| *LIPG* | -1.589 | 0.022 | -1.625 | 0.020 |
| *LMCD1* | 5.390 | 0.000 | 6.042 | 0.000 |
| *LOC100130152* | 1.950 | 0.025 | 2.156 | 0.014 |
| *LOC100130232* | -1.329 | 0.063 | -1.322 | 0.064 |
| *LOC100130540* | -1.873 | 0.029 | -1.608 | 0.056 |
| *LOC100130560* | -2.452 | 0.017 | -3.060 | 0.004 |
| *LOC100130899* | -3.231 | 0.005 | -2.680 | 0.016 |
| *LOC100131907* | -2.614 | 0.016 | -2.509 | 0.020 |
| *LOC100134423* | -1.654 | 0.036 | -2.033 | 0.013 |
| *LOC100287477* | -2.013 | 0.056 | -2.700 | 0.014 |
| *LOC100288619* | -1.263 | 0.048 | -1.340 | 0.037 |
| *LOC100505923* | -2.511 | 0.012 | -2.568 | 0.011 |
| *LOC100506314* | -1.646 | 0.046 | -2.120 | 0.013 |
| *LOC100506411* | 2.372 | 0.016 | 1.905 | 0.047 |
| *LOC100506674* | 5.205 | 0.001 | 3.913 | 0.010 |
| *LOC100506827* | -1.602 | 0.026 | -1.662 | 0.022 |
| *LOC100507131* | -1.885 | 0.015 | -1.750 | 0.023 |
| *LOC100507291* | 1.717 | 0.099 | 1.731 | 0.097 |
| *LOC100507599* | -1.968 | 0.031 | -1.621 | 0.070 |
| *LOC100652768* | -2.494 | 0.021 | -2.293 | 0.032 |
| *LOC100996535* | -1.937 | 0.075 | -1.813 | 0.094 |
| *LOC101926934* | -1.651 | 0.023 | -1.531 | 0.033 |
| *LOC101927322* | -1.802 | 0.046 | -2.040 | 0.026 |
| *LOC101927331* | -1.871 | 0.065 | -2.164 | 0.036 |
| *LOC101927354* | -1.522 | 0.044 | -1.329 | 0.074 |
| *LOC101927389* | -1.603 | 0.057 | -1.461 | 0.080 |
| *LOC101927584* | -1.293 | 0.068 | -1.205 | 0.086 |
| *LOC101927770* | 2.588 | 0.088 | 3.045 | 0.048 |
| *LOC101928201* | -1.581 | 0.019 | -1.495 | 0.025 |
| *LOC101928347* | -1.061 | 0.067 | -1.256 | 0.033 |
| *LOC101928413* | 1.961 | 0.026 | 1.734 | 0.045 |
| *LOC101928659* | 1.926 | 0.084 | 2.596 | 0.024 |

| | | | | |
|---|---|---|---|---|
| *LOC101928865* | 1.762 | 0.060 | 1.808 | 0.054 |
| *LOC101929128* | -1.874 | 0.078 | -2.323 | 0.033 |
| *LOC101929181* | -1.693 | 0.079 | -1.717 | 0.076 |
| *LOC101929231* | -2.270 | 0.036 | -1.742 | 0.098 |
| *LOC101929998* | -2.250 | 0.072 | -3.805 | 0.005 |
| *LOC102723428* | 2.181 | 0.009 | 1.537 | 0.052 |
| *LOC102723505* | -6.450 | 0.000 | -6.291 | 0.000 |
| *LOC102723765* | -1.788 | 0.046 | -1.941 | 0.032 |
| *LOC102723854* | -2.744 | 0.001 | -3.526 | 0.000 |
| *LOC102723882* | -1.863 | 0.035 | -1.716 | 0.050 |
| *LOC102723920* | -3.106 | 0.011 | -2.595 | 0.029 |
| *LOC102724505* | -1.509 | 0.036 | -1.310 | 0.064 |
| *LOC102725242* | -1.399 | 0.084 | -1.378 | 0.088 |
| *LOC102725394* | -3.038 | 0.031 | -4.622 | 0.002 |
| *LOC102800447* | -2.212 | 0.008 | -2.296 | 0.006 |
| *LOC154761* | -2.711 | 0.016 | -2.561 | 0.021 |
| *LOC283299* | -1.653 | 0.054 | -1.960 | 0.025 |
| *LOC339988* | -1.133 | 0.099 | -2.037 | 0.006 |
| *LOC340017* | -2.239 | 0.042 | -2.045 | 0.060 |
| *LOC388242* | -2.389 | 0.004 | -2.690 | 0.001 |
| *LOC401287* | -2.472 | 0.060 | -2.281 | 0.080 |
| *LOC401296* | -1.499 | 0.073 | -1.869 | 0.029 |
| *LOC440300* | 2.189 | 0.097 | 2.878 | 0.034 |
| *LOC541473* | -2.646 | 0.044 | -2.634 | 0.045 |
| *LOC643454* | 1.896 | 0.024 | 1.664 | 0.044 |
| *LOC643733* | -2.994 | 0.008 | -2.813 | 0.011 |
| *LOC644070* | -1.788 | 0.054 | -2.139 | 0.024 |
| *LOC644919* | -1.833 | 0.035 | -1.895 | 0.030 |
| *LOC645188* | -1.713 | 0.029 | -1.274 | 0.093 |
| *LOC728040* | -1.271 | 0.090 | -1.435 | 0.059 |
| *LOC729047* | -2.165 | 0.011 | -2.208 | 0.010 |
| *LOC93432* | -1.738 | 0.017 | -1.830 | 0.013 |
| *LOXL3* | 2.823 | 0.051 | 2.868 | 0.048 |
| *LPCAT1* | 3.811 | 0.007 | 2.632 | 0.049 |
| *LRFN1* | -2.556 | 0.075 | -2.659 | 0.065 |
| *LRG1* | 3.790 | 0.064 | 3.703 | 0.070 |
| *LRRC42* | 2.179 | 0.058 | 2.293 | 0.048 |
| *LRRC56* | 3.013 | 0.025 | 3.032 | 0.025 |
| *LRRFIP1* | 1.868 | 0.095 | 2.660 | 0.022 |
| *LRRN2* | 2.718 | 0.071 | 2.985 | 0.049 |
| *LRRN4CL* | 3.000 | 0.041 | 3.525 | 0.019 |
| *LRRTM4* | -1.718 | 0.095 | -1.732 | 0.093 |
| *LTB* | -3.045 | 0.011 | -2.651 | 0.023 |
| *LTBP1* | 4.948 | 0.005 | 3.402 | 0.042 |
| *LTBP2* | -2.676 | 0.041 | -2.126 | 0.096 |
| *LUC7L2* | -2.566 | 0.011 | -2.435 | 0.015 |
| *LUM* | 3.865 | 0.004 | 4.755 | 0.001 |
| *LUZP2* | -3.344 | 0.008 | -2.436 | 0.044 |
| *LY75* | -4.050 | 0.014 | -4.853 | 0.004 |
| *LY9* | 1.812 | 0.024 | 1.518 | 0.054 |
| *LYN* | -3.238 | 0.008 | -2.883 | 0.017 |
| *LYPD6* | -2.492 | 0.054 | -3.103 | 0.020 |
| *LYRM9* | -4.852 | 0.003 | -3.363 | 0.030 |
| *MAB21L1* | -1.386 | 0.040 | -1.088 | 0.099 |
| *MAG* | -1.763 | 0.057 | -1.646 | 0.074 |

| | | | | |
|---|---|---|---|---|
| *MAGI1* | -3.338 | 0.059 | -4.007 | 0.027 |
| *MAGI2-AS3* | -2.461 | 0.049 | -3.263 | 0.012 |
| *MAL2* | 2.327 | 0.019 | 1.946 | 0.045 |
| *MAML3* | -3.152 | 0.078 | -3.328 | 0.064 |
| *MAP2K6* | -2.630 | 0.016 | -4.515 | 0.000 |
| *MAP3K12* | 2.365 | 0.026 | 1.966 | 0.058 |
| *MAPK9* | 2.469 | 0.046 | 3.256 | 0.011 |
| *MAPKAPK2* | 1.478 | 0.076 | 1.558 | 0.063 |
| *MAST4* | 2.228 | 0.098 | 3.520 | 0.013 |
| *MASTL* | 3.283 | 0.049 | 3.579 | 0.034 |
| *MATN2* | -5.135 | 0.001 | -4.883 | 0.001 |
| *MBNL1-AS1* | -1.551 | 0.062 | -1.488 | 0.073 |
| *MED20* | 1.701 | 0.061 | 1.824 | 0.046 |
| *MED27* | -1.763 | 0.050 | -2.095 | 0.023 |
| *MEN1* | 2.291 | 0.049 | 2.187 | 0.059 |
| *MESP1* | -2.444 | 0.018 | -1.945 | 0.052 |
| *METTL10* | 1.855 | 0.042 | 1.864 | 0.042 |
| *METTL7A* | -1.789 | 0.083 | -3.296 | 0.004 |
| *MFI2* | -2.873 | 0.023 | -3.043 | 0.017 |
| *MIA* | -4.018 | 0.007 | -7.409 | 0.000 |
| *MID1* | -2.831 | 0.018 | -2.419 | 0.038 |
| *MIEF1* | -2.549 | 0.033 | -2.315 | 0.050 |
| *MIR100HG* | -3.264 | 0.078 | -3.329 | 0.073 |
| *MIR31HG* | -2.201 | 0.040 | -2.299 | 0.033 |
| *MIR3916* | 2.218 | 0.033 | 2.143 | 0.039 |
| *MIR503HG* | 1.585 | 0.040 | 1.762 | 0.024 |
| *MITD1* | 3.063 | 0.005 | 2.148 | 0.036 |
| *MMD* | 2.609 | 0.060 | 2.589 | 0.061 |
| *MME* | -4.328 | 0.002 | -4.214 | 0.002 |
| *MMP1* | 3.555 | 0.006 | 2.363 | 0.051 |
| *MMP11* | 3.624 | 0.016 | 4.978 | 0.002 |
| *MMP7* | -5.326 | 0.010 | -7.057 | 0.001 |
| *MOAP1* | 2.932 | 0.003 | 1.727 | 0.058 |
| *MOB3B* | -3.320 | 0.007 | -2.399 | 0.040 |
| *MPC2* | 2.695 | 0.007 | 2.794 | 0.005 |
| *MPHOSPH10* | 2.878 | 0.029 | 2.968 | 0.025 |
| *MRAS* | -2.697 | 0.014 | -3.013 | 0.007 |
| *MRGBP* | 2.332 | 0.022 | 3.087 | 0.004 |
| *MRPL27* | 2.202 | 0.039 | 2.686 | 0.014 |
| *MRPL28* | 2.978 | 0.068 | 3.103 | 0.058 |
| *MRPL47* | 3.378 | 0.015 | 2.779 | 0.040 |
| *MRPL55* | 1.307 | 0.036 | 1.632 | 0.011 |
| *MRPS30* | 3.293 | 0.025 | 2.589 | 0.069 |
| *MRVI1* | 1.848 | 0.089 | 3.023 | 0.009 |
| *MRVI1-AS1* | -2.012 | 0.086 | -1.967 | 0.093 |
| *MSI1* | -2.687 | 0.067 | -2.823 | 0.056 |
| *MSL3P1* | -2.547 | 0.007 | -1.548 | 0.082 |
| *MSMB* | 6.000 | 0.000 | 4.127 | 0.004 |
| *MSRA* | -2.854 | 0.044 | -4.766 | 0.002 |
| *MSRB1* | 1.745 | 0.096 | 2.249 | 0.036 |
| *MST1R* | 2.434 | 0.050 | 3.825 | 0.004 |
| *MSTO1* | 2.022 | 0.075 | 1.898 | 0.093 |
| *MT1A* | -1.642 | 0.034 | -1.834 | 0.019 |
| *MT1E* | -2.409 | 0.005 | -2.817 | 0.002 |
| *MT1G* | -2.193 | 0.020 | -4.823 | 0.000 |

| | | | | |
|---|---|---|---|---|
| *MT1HL1* | -1.796 | 0.040 | -2.219 | 0.014 |
| *MT1L* | -1.799 | 0.018 | -1.890 | 0.014 |
| *MT1M* | -3.188 | 0.010 | -5.596 | 0.000 |
| *MT1X* | -1.867 | 0.038 | -2.650 | 0.005 |
| *MTA1* | 3.820 | 0.009 | 3.030 | 0.031 |
| *MTF2* | 2.787 | 0.033 | 2.715 | 0.037 |
| *MTX1* | 1.831 | 0.054 | 1.617 | 0.085 |
| *MUC1* | 2.313 | 0.003 | 2.098 | 0.006 |
| *MUC15* | -3.744 | 0.004 | -3.086 | 0.015 |
| *MUC7* | -2.685 | 0.045 | -2.275 | 0.084 |
| *MUTYH* | 2.956 | 0.032 | 3.573 | 0.012 |
| *MVB12B* | -2.918 | 0.050 | -3.041 | 0.042 |
| *MYC* | -3.178 | 0.006 | -4.012 | 0.001 |
| *MYCBP* | 2.809 | 0.005 | 2.351 | 0.015 |
| *MYL5* | -1.732 | 0.036 | -1.547 | 0.058 |
| *MYLK* | -1.545 | 0.028 | -2.371 | 0.002 |
| *NAA10* | 2.254 | 0.049 | 2.996 | 0.012 |
| *NAGK* | 1.545 | 0.065 | 1.394 | 0.093 |
| *NAP1L2* | -2.435 | 0.011 | -2.335 | 0.013 |
| *NARF* | 2.231 | 0.067 | 2.647 | 0.033 |
| *NAT1* | 5.978 | 0.000 | 5.657 | 0.000 |
| *NAT2* | 3.481 | 0.013 | 2.360 | 0.077 |
| *NBPF10* | 1.401 | 0.012 | 1.216 | 0.025 |
| *NBPF6* | 3.111 | 0.064 | 4.094 | 0.019 |
| *NBPF9* | 1.590 | 0.035 | 1.331 | 0.071 |
| *NCALD* | -4.083 | 0.006 | -4.878 | 0.002 |
| *NCKAP1L* | -1.724 | 0.027 | -1.737 | 0.026 |
| *NCMAP* | -2.374 | 0.009 | -5.249 | 0.000 |
| *NDFIP1* | 1.857 | 0.051 | 2.421 | 0.014 |
| *NDRG2* | -2.820 | 0.041 | -4.245 | 0.004 |
| *NDUFAB1* | 2.075 | 0.060 | 2.871 | 0.013 |
| *NDUFB6* | 2.949 | 0.021 | 3.260 | 0.012 |
| *NEDD4L* | -3.474 | 0.006 | -2.434 | 0.042 |
| *NEIL1* | -2.997 | 0.043 | -3.746 | 0.014 |
| *NENF* | 1.173 | 0.067 | 1.270 | 0.049 |
| *NES* | -3.898 | 0.013 | -3.764 | 0.016 |
| *NEURL3* | -4.162 | 0.005 | -4.825 | 0.002 |
| *NFIB* | -2.820 | 0.003 | -3.567 | 0.000 |
| *NIPSNAP1* | 1.523 | 0.063 | 2.190 | 0.011 |
| *NOTCH4* | -2.151 | 0.072 | -2.749 | 0.025 |
| *NPAT* | -2.099 | 0.043 | -2.368 | 0.025 |
| *NPNT* | 2.452 | 0.088 | 3.527 | 0.019 |
| *NPRL3* | 1.845 | 0.075 | 2.630 | 0.015 |
| *NR3C1* | -1.222 | 0.080 | -1.546 | 0.031 |
| *NT5DC3* | -1.824 | 0.058 | 1.652 | 0.083 |
| *NTRK3* | -2.014 | 0.064 | -1.876 | 0.083 |
| *NUCKS1* | 1.510 | 0.027 | 1.498 | 0.029 |
| *NUDT5* | 1.942 | 0.093 | 1.978 | 0.088 |
| *NUDT6* | -5.234 | 0.000 | -4.603 | 0.000 |
| *NUDT9P1* | -4.438 | 0.004 | -3.635 | 0.013 |
| *NUF2* | 2.650 | 0.029 | 2.201 | 0.064 |
| *NUP37* | 2.224 | 0.023 | 2.277 | 0.020 |
| *NUSAP1* | 3.581 | 0.006 | 3.146 | 0.014 |
| *NVL* | 3.505 | 0.031 | 3.812 | 0.020 |
| *OGFRL1* | -2.256 | 0.078 | -3.132 | 0.018 |

| | | | | |
|---|---|---|---|---|
| *OR10AD1* | -1.305 | 0.097 | -1.473 | 0.064 |
| *OR12D2* | -1.564 | 0.028 | -1.416 | 0.044 |
| *OR1L3* | -1.780 | 0.048 | -1.495 | 0.092 |
| *OXTR* | -2.684 | 0.028 | -3.224 | 0.011 |
| *P2RY12* | -2.017 | 0.062 | -1.853 | 0.084 |
| *PADI2* | -3.253 | 0.011 | -3.584 | 0.006 |
| *PAK7* | -2.789 | 0.006 | -2.007 | 0.038 |
| *PALMD* | -2.618 | 0.067 | -3.681 | 0.014 |
| *PANK2* | 3.052 | 0.024 | 2.271 | 0.083 |
| *PARD6B* | 2.962 | 0.012 | 2.052 | 0.069 |
| *PARP14* | 1.657 | 0.030 | 1.840 | 0.018 |
| *PBK* | 2.717 | 0.055 | 3.042 | 0.034 |
| *PCDH15* | -3.304 | 0.030 | -2.930 | 0.051 |
| *PCDHB4* | -2.729 | 0.040 | -2.383 | 0.069 |
| *PDCD5* | 1.429 | 0.077 | 1.467 | 0.070 |
| *PDE8A* | 3.003 | 0.013 | 2.378 | 0.042 |
| *PDGFA* | -2.087 | 0.089 | -4.582 | 0.001 |
| *PDGFRA* | -3.439 | 0.003 | -3.246 | 0.005 |
| *PDK4* | -3.981 | 0.003 | -5.753 | 0.000 |
| *PDLIM3* | -3.019 | 0.038 | -2.464 | 0.084 |
| *PDP1* | 2.148 | 0.007 | 1.638 | 0.033 |
| *PER1* | -2.469 | 0.034 | -3.249 | 0.008 |
| *PGAM1* | 2.224 | 0.011 | 1.486 | 0.072 |
| *PHF1* | -2.060 | 0.043 | -3.575 | 0.002 |
| *PHF13* | -2.134 | 0.055 | -2.914 | 0.012 |
| *PHLDA1* | -3.000 | 0.004 | -3.334 | 0.002 |
| *PI15* | -5.018 | 0.001 | -6.295 | 0.000 |
| *PI4K2A* | -2.685 | 0.053 | -2.274 | 0.095 |
| *PICK1* | -2.829 | 0.005 | -3.071 | 0.003 |
| *PIGC* | 2.301 | 0.042 | 2.228 | 0.049 |
| *PIGN* | 2.101 | 0.029 | 2.799 | 0.005 |
| *PIGR* | -7.006 | 0.002 | -7.964 | 0.001 |
| *PIK3C3* | 2.996 | 0.058 | 3.128 | 0.048 |
| *PIK3CG* | -2.533 | 0.090 | -2.523 | 0.091 |
| *PINLYP* | -3.862 | 0.000 | -4.034 | 0.000 |
| *PITX1* | 2.851 | 0.058 | 3.699 | 0.018 |
| *PKD1P6* | 3.863 | 0.010 | 2.571 | 0.070 |
| *PKN3* | -2.108 | 0.097 | -2.642 | 0.042 |
| *PLA2G6* | -1.691 | 0.096 | -2.569 | 0.016 |
| *PLAGL1* | -3.288 | 0.001 | -1.741 | 0.056 |
| *PLCD1* | -2.173 | 0.098 | -2.821 | 0.036 |
| *PLCH1* | -1.734 | 0.046 | -1.762 | 0.043 |
| *PLEKHM3* | -2.201 | 0.090 | -4.884 | 0.001 |
| *PLK1* | 2.831 | 0.034 | 2.471 | 0.089 |
| *PLOD2* | 3.300 | 0.001 | 2.716 | 0.003 |
| *PLS3* | -2.049 | 0.048 | -1.928 | 0.062 |
| *PMAIP1* | 2.678 | 0.038 | 2.172 | 0.086 |
| *PMF1* | 2.556 | 0.044 | 2.466 | 0.051 |
| *PMPCA* | -2.124 | 0.064 | -2.049 | 0.073 |
| *PMS2L2* | 2.381 | 0.051 | 2.240 | 0.064 |
| *PMVK* | 2.974 | 0.052 | 2.521 | 0.095 |
| *PNPLA7* | -2.027 | 0.036 | -1.841 | 0.054 |
| *PNRC1* | -1.695 | 0.030 | -2.027 | 0.012 |
| *PODXL2* | -1.471 | 0.051 | -1.546 | 0.042 |
| *POLD3* | 3.947 | 0.016 | 4.796 | 0.005 |

| | | | | |
|---|---|---|---|---|
| *POLQ* | 2.419 | 0.005 | 2.225 | 0.009 |
| *POLR1E* | -2.122 | 0.057 | -2.303 | 0.040 |
| *POLR2C* | -1.834 | 0.079 | -1.786 | 0.086 |
| *POSTN* | 3.748 | 0.004 | 4.578 | 0.001 |
| *POTEB3* | 3.955 | 0.000 | 3.180 | 0.002 |
| *POTED* | 2.544 | 0.032 | 2.585 | 0.030 |
| *POU5F2* | -2.507 | 0.009 | -2.040 | 0.027 |
| *PPARA* | -1.905 | 0.044 | -1.691 | 0.070 |
| *PPFIA1* | 3.176 | 0.003 | 4.354 | 0.000 |
| *PPM1F* | -2.628 | 0.027 | -2.253 | 0.054 |
| *PPP1R15A* | -3.729 | 0.002 | -3.195 | 0.005 |
| *PPP1R1A* | -1.856 | 0.034 | -1.714 | 0.048 |
| *PRB3* | -1.287 | 0.086 | -2.037 | 0.010 |
| *PRDX2* | 1.407 | 0.084 | 1.380 | 0.090 |
| *PRKCQ* | -2.064 | 0.034 | -2.172 | 0.027 |
| *PRKCQ-AS1* | -3.662 | 0.004 | -2.553 | 0.034 |
| *PRKX* | -2.989 | 0.023 | -3.535 | 0.009 |
| *PRMT5* | 2.707 | 0.016 | 2.572 | 0.021 |
| *PRPF3* | 3.337 | 0.027 | 2.605 | 0.075 |
| *PRR19* | 2.068 | 0.023 | 2.054 | 0.024 |
| *PRR34* | -1.330 | 0.056 | -1.540 | 0.030 |
| *PRR34-AS1* | -1.920 | 0.025 | -2.343 | 0.008 |
| *PRRT3* | 3.872 | 0.002 | 5.126 | 0.000 |
| *PRSS21* | -1.804 | 0.030 | -1.431 | 0.077 |
| *PRSS3* | -1.939 | 0.030 | -1.718 | 0.052 |
| *PSCA* | 2.071 | 0.045 | 3.296 | 0.003 |
| *PSEN2* | 3.610 | 0.019 | 3.173 | 0.035 |
| *PSMC1* | 1.735 | 0.066 | 1.677 | 0.075 |
| *PSMC4* | 1.188 | 0.089 | 1.558 | 0.030 |
| *PSMD2* | 1.657 | 0.040 | 1.432 | 0.071 |
| *PSMD4* | 1.176 | 0.046 | 1.450 | 0.017 |
| *PTGS2* | -1.910 | 0.078 | -1.975 | 0.069 |
| *PTN* | -4.036 | 0.026 | -5.222 | 0.006 |
| *PTPN13* | 2.124 | 0.078 | 2.263 | 0.062 |
| *PTPRO* | -1.677 | 0.099 | -1.830 | 0.074 |
| *PTRF* | -1.610 | 0.079 | -2.586 | 0.008 |
| *QRFPR* | -2.699 | 0.059 | -2.940 | 0.042 |
| *RAB14* | -2.637 | 0.027 | -3.677 | 0.004 |
| *RAB30* | 2.201 | 0.098 | 2.224 | 0.095 |
| *RABAC1* | 1.274 | 0.045 | 1.564 | 0.017 |
| *RAD17* | -2.498 | 0.013 | -2.049 | 0.036 |
| *RAD21* | 1.777 | 0.018 | 1.323 | 0.068 |
| *RANBP3L* | -2.230 | 0.027 | -2.685 | 0.010 |
| *RAPH1* | 1.755 | 0.041 | 1.536 | 0.070 |
| *RARRES1* | -3.680 | 0.012 | -3.840 | 0.009 |
| *RASA3* | -2.395 | 0.004 | -1.997 | 0.012 |
| *RASAL1* | -1.840 | 0.074 | -2.203 | 0.036 |
| *RASAL2* | -2.322 | 0.045 | -4.031 | 0.002 |
| *RASGEF1B* | -2.179 | 0.093 | -2.336 | 0.074 |
| *RASGRF2-AS1* | -1.246 | 0.095 | -1.237 | 0.097 |
| *RBAK* | 4.520 | 0.009 | 2.866 | 0.078 |
| *RBBP8* | 1.913 | 0.044 | 1.925 | 0.043 |
| *RBM24* | 3.160 | 0.035 | 2.468 | 0.091 |
| *RBM25* | 2.842 | 0.022 | 2.126 | 0.077 |
| *RBM34* | 2.970 | 0.020 | 2.401 | 0.054 |

| | | | | |
|---|---|---|---|---|
| *RBM6* | 2.449 | 0.005 | 3.974 | 0.000 |
| *RBM8A* | 1.871 | 0.019 | 2.254 | 0.006 |
| *RBMS3* | -3.033 | 0.019 | -2.570 | 0.042 |
| *RBSG2* | -1.719 | 0.014 | -1.784 | 0.011 |
| *RCBTB1* | 4.310 | 0.009 | 3.084 | 0.050 |
| *RECQL* | 2.091 | 0.049 | 3.304 | 0.004 |
| *REEP2* | 1.909 | 0.053 | 2.025 | 0.042 |
| *REEP4* | 3.582 | 0.012 | 4.068 | 0.005 |
| *RERGL* | -3.622 | 0.006 | -3.071 | 0.016 |
| *RFTN1* | 2.980 | 0.012 | 2.530 | 0.030 |
| *RGN* | -3.232 | 0.004 | -2.124 | 0.045 |
| *RGS2* | -4.400 | 0.016 | -4.384 | 0.016 |
| *RIMS3* | -3.602 | 0.004 | -2.649 | 0.025 |
| *RIMS4* | 3.174 | 0.056 | 3.662 | 0.030 |
| *RIPPLY3* | 2.973 | 0.021 | 2.047 | 0.099 |
| *RMI1* | 3.636 | 0.001 | 2.418 | 0.022 |
| *RNF103* | 1.549 | 0.024 | 1.185 | 0.074 |
| *RNF150* | -2.919 | 0.003 | -2.625 | 0.007 |
| *RNF170* | 3.046 | 0.008 | 1.771 | 0.095 |
| *RNF213* | 4.151 | 0.048 | 4.071 | 0.052 |
| *RNU11* | -1.592 | 0.043 | -2.080 | 0.011 |
| *ROPN1* | -4.528 | 0.000 | -5.081 | 0.000 |
| *RP2* | 2.653 | 0.002 | 2.470 | 0.003 |
| *RPAP3* | 1.790 | 0.011 | 1.288 | 0.054 |
| *RPF1* | 2.410 | 0.013 | 1.990 | 0.034 |
| *RPH3AL* | 3.244 | 0.013 | 3.763 | 0.005 |
| *RPL10A* | -1.272 | 0.022 | -0.945 | 0.077 |
| *RPL13* | -1.348 | 0.012 | -1.200 | 0.022 |
| *RPL13A* | -1.153 | 0.059 | -1.082 | 0.074 |
| *RPL18A* | -1.408 | 0.035 | -1.276 | 0.054 |
| *RPL21P44* | -1.936 | 0.002 | -1.651 | 0.006 |
| *RPL26L1* | 3.892 | 0.016 | 2.836 | 0.069 |
| *RPL7A* | -1.081 | 0.051 | -1.150 | 0.039 |
| *RPS18* | -1.289 | 0.045 | -1.281 | 0.046 |
| *RPS19* | -1.482 | 0.044 | -1.248 | 0.085 |
| *RPS27A* | -1.563 | 0.014 | -1.646 | 0.011 |
| *RPS6KC1* | 1.346 | 0.082 | 1.275 | 0.098 |
| *RPUSD2* | -2.778 | 0.088 | -3.936 | 0.020 |
| *RRAS* | 1.846 | 0.044 | 2.613 | 0.007 |
| *RRM2* | 4.440 | 0.005 | 4.135 | 0.008 |
| *RUNX3* | -3.020 | 0.059 | -3.427 | 0.035 |
| *RUSC1* | 1.803 | 0.092 | 1.929 | 0.073 |
| *S100A10* | 1.543 | 0.018 | 1.700 | 0.010 |
| *S100A14* | 2.842 | 0.004 | 2.788 | 0.005 |
| *S100A4* | 2.703 | 0.063 | 2.782 | 0.057 |
| *S100A7* | 9.062 | 0.000 | 8.425 | 0.000 |
| *S100A9* | 3.967 | 0.002 | 2.526 | 0.030 |
| *S100B* | -2.066 | 0.007 | -2.514 | 0.002 |
| *S100P* | 5.401 | 0.005 | 4.982 | 0.008 |
| *S1PR3* | 2.554 | 0.027 | 2.105 | 0.062 |
| *SAA4* | -2.274 | 0.044 | -3.304 | 0.006 |
| *SAMD5* | -4.546 | 0.000 | -5.010 | 0.000 |
| *SAMD9L* | 3.144 | 0.016 | 2.151 | 0.086 |
| *SAP30BP* | 2.467 | 0.068 | 2.663 | 0.050 |
| *SAR1A* | 2.258 | 0.026 | 1.649 | 0.092 |

| | | | | |
|---|---|---|---|---|
| *SARM1* | -3.514 | 0.007 | -3.373 | 0.009 |
| *SARS* | -1.339 | 0.052 | -1.258 | 0.067 |
| *SASH1* | -2.949 | 0.017 | -2.905 | 0.018 |
| *SCAMP1* | 2.096 | 0.020 | 2.172 | 0.016 |
| *SCAMP3* | 1.588 | 0.022 | 1.517 | 0.027 |
| *SCD* | 4.781 | 0.000 | 3.746 | 0.001 |
| *SCG2* | 1.158 | 0.094 | 1.656 | 0.021 |
| *SCGB3A2* | -1.924 | 0.060 | -2.132 | 0.040 |
| *SCN2B* | -2.844 | 0.008 | -2.784 | 0.010 |
| *SCN3B* | -3.020 | 0.001 | -2.878 | 0.002 |
| *SCN4B* | -3.419 | 0.015 | -3.742 | 0.009 |
| *SCNN1B* | -1.680 | 0.081 | -1.920 | 0.049 |
| *SDF2* | 2.587 | 0.078 | 2.656 | 0.071 |
| *SEC22B* | 1.317 | 0.026 | 0.989 | 0.084 |
| *SEC23A* | 2.877 | 0.036 | 2.434 | 0.070 |
| *SEC62* | 1.672 | 0.076 | 1.698 | 0.072 |
| *SEMA3B* | 4.449 | 0.000 | 4.437 | 0.000 |
| *SEMA3B-AS1* | 3.257 | 0.024 | 2.769 | 0.051 |
| *SEMA5A* | -3.198 | 0.015 | -3.471 | 0.009 |
| *SEMA6D* | -1.628 | 0.055 | -1.747 | 0.041 |
| *SEPT4* | -1.930 | 0.034 | -1.504 | 0.090 |
| *SEPT7* | 2.562 | 0.008 | 2.524 | 0.008 |
| *SERPINA1* | 2.204 | 0.044 | 2.023 | 0.062 |
| *SERPINB5* | -2.957 | 0.050 | -5.999 | 0.001 |
| *SERPINH1* | 1.508 | 0.052 | 1.293 | 0.091 |
| *SERTAD2* | 2.089 | 0.055 | 3.422 | 0.004 |
| *SF3B2* | 1.966 | 0.023 | 2.080 | 0.017 |
| *SFRP1* | -5.240 | 0.000 | -5.572 | 0.000 |
| *SFRP4* | 3.417 | 0.000 | 4.870 | 0.000 |
| *SFT2D2* | -2.760 | 0.074 | -2.750 | 0.075 |
| *SGK1* | -2.479 | 0.018 | -2.051 | 0.044 |
| *SGSH* | -3.049 | 0.019 | -2.282 | 0.069 |
| *SH3BGRL* | 1.847 | 0.012 | 1.342 | 0.056 |
| *SH3BGRL2* | -3.401 | 0.030 | -4.140 | 0.011 |
| *SH3BGRL3* | 2.408 | 0.011 | 1.654 | 0.065 |
| *SIAH2* | 2.295 | 0.021 | 1.661 | 0.083 |
| *SIDT1* | 3.547 | 0.020 | 3.115 | 0.037 |
| *SIGIRR* | 2.722 | 0.004 | 2.527 | 0.006 |
| *SIGLEC8* | -2.143 | 0.028 | -2.726 | 0.007 |
| *SKAP2* | -4.251 | 0.003 | -3.651 | 0.009 |
| *SLC16A3* | 2.729 | 0.043 | 3.918 | 0.006 |
| *SLC1A1* | 3.595 | 0.028 | 3.484 | 0.032 |
| *SLC22A1* | -2.277 | 0.006 | -1.988 | 0.015 |
| *SLC24A1* | -2.194 | 0.053 | -2.075 | 0.065 |
| *SLC25A39* | 1.886 | 0.044 | 1.689 | 0.069 |
| *SLC25A44* | 2.320 | 0.017 | 2.485 | 0.011 |
| *SLC27A2* | 3.922 | 0.032 | 3.669 | 0.043 |
| *SLC2A10* | 3.372 | 0.021 | 3.353 | 0.022 |
| *SLC2A4RG* | 1.511 | 0.066 | 1.367 | 0.093 |
| *SLC35F2* | 2.604 | 0.030 | 3.075 | 0.012 |
| *SLC35F5* | 2.695 | 0.074 | 3.236 | 0.036 |
| *SLC39A6* | 3.660 | 0.001 | 2.659 | 0.007 |
| *SLC4A11* | -3.117 | 0.013 | -3.617 | 0.005 |
| *SLC5A9* | -1.440 | 0.090 | -1.952 | 0.026 |
| *SLC6A14* | -5.878 | 0.004 | -6.232 | 0.003 |

| | | | | |
|---|---|---|---|---|
| *SLC7A2* | 3.117 | 0.004 | 3.088 | 0.004 |
| *SLFN12* | -1.905 | 0.031 | -1.741 | 0.047 |
| *SLITRK6* | 3.031 | 0.041 | 3.200 | 0.032 |
| *SMAD3* | -1.849 | 0.063 | -1.934 | 0.053 |
| *SMAD5* | 1.715 | 0.068 | 1.635 | 0.081 |
| *SMARCC2* | 3.767 | 0.011 | 2.758 | 0.052 |
| *SMCO4* | -2.615 | 0.067 | -3.691 | 0.013 |
| *SMIM17* | 3.243 | 0.027 | 3.682 | 0.013 |
| *SMOX* | -1.712 | 0.031 | -2.385 | 0.004 |
| *SMYD1* | -1.877 | 0.032 | -1.782 | 0.041 |
| *SMYD4* | -3.940 | 0.031 | -3.906 | 0.032 |
| *SNAPC1* | -1.339 | 0.057 | -1.682 | 0.020 |
| *SND1* | -1.456 | 0.027 | -1.478 | 0.025 |
| *SNHG8* | -1.095 | 0.095 | -1.524 | 0.025 |
| *SNORA37* | 2.086 | 0.092 | 2.041 | 0.098 |
| *SNORD104* | 4.456 | 0.011 | 3.969 | 0.022 |
| *SNORD48* | 2.015 | 0.011 | 2.076 | 0.009 |
| *SNORD6* | 2.562 | 0.013 | 2.552 | 0.014 |
| *SNORD75* | 2.270 | 0.031 | 3.479 | 0.002 |
| *SNORD76* | 3.971 | 0.009 | 2.640 | 0.068 |
| *SNRPB2* | 1.236 | 0.058 | 1.368 | 0.038 |
| *SNRPE* | 1.253 | 0.025 | 0.973 | 0.073 |
| *SNRPG* | 1.114 | 0.052 | 0.939 | 0.096 |
| *SOCS2* | -4.446 | 0.003 | -5.053 | 0.001 |
| *SOSTDC1* | -4.276 | 0.000 | -3.133 | 0.005 |
| *SOX10* | -5.259 | 0.000 | -4.090 | 0.007 |
| *SOX8* | -1.853 | 0.025 | -2.445 | 0.005 |
| *SOX9-AS1* | -2.505 | 0.035 | -2.775 | 0.021 |
| *SP110* | 3.109 | 0.011 | 1.923 | 0.097 |
| *SPAG4* | 2.182 | 0.021 | 2.413 | 0.012 |
| *SPATA18* | -2.714 | 0.010 | -3.020 | 0.005 |
| *SPATA6L* | 1.858 | 0.014 | 1.405 | 0.053 |
| *SPCS2* | 1.571 | 0.011 | 1.468 | 0.017 |
| *SPECC1* | 2.694 | 0.007 | 1.547 | 0.095 |
| *SPESP1* | -1.784 | 0.036 | -1.441 | 0.084 |
| *SPPL3* | 3.077 | 0.020 | 2.950 | 0.025 |
| *SPRN* | -2.529 | 0.037 | -2.591 | 0.033 |
| *SPRY2* | -3.882 | 0.001 | -4.008 | 0.001 |
| *SPTLC3* | -3.272 | 0.041 | -3.654 | 0.024 |
| *SPTSSA* | 1.525 | 0.034 | 1.386 | 0.051 |
| *SRCAP* | 1.462 | 0.070 | 2.193 | 0.010 |
| *SREBF1* | 2.916 | 0.003 | 2.690 | 0.005 |
| *SREK1* | 3.159 | 0.016 | 3.243 | 0.014 |
| *SRGAP2* | 3.088 | 0.015 | 4.303 | 0.002 |
| *SRP9* | 1.050 | 0.037 | 0.969 | 0.052 |
| *SRRM2-AS1* | 1.770 | 0.060 | 2.831 | 0.005 |
| *SRSF11* | 1.220 | 0.057 | 1.159 | 0.069 |
| *ST3GAL6* | -3.688 | 0.000 | -4.219 | 0.000 |
| *STAC* | -1.483 | 0.045 | -1.339 | 0.067 |
| *STAC2* | -3.798 | 0.000 | -3.639 | 0.000 |
| *STARD10* | 3.101 | 0.005 | 2.904 | 0.007 |
| *STX2* | 2.316 | 0.083 | 3.670 | 0.010 |
| *STX7* | -1.847 | 0.058 | -1.745 | 0.072 |
| *STYK1* | 2.242 | 0.083 | 3.139 | 0.019 |
| *STYXL1* | 2.754 | 0.031 | 2.988 | 0.021 |

| | | | | |
|---|---|---|---|---|
| *SUCO* | 1.700 | 0.014 | 1.191 | 0.071 |
| *SUFU* | -3.306 | 0.016 | -2.154 | 0.098 |
| *SVIL* | -1.926 | 0.083 | -3.709 | 0.003 |
| *SYAP1* | 3.645 | 0.029 | 3.535 | 0.033 |
| *SYNM* | -5.387 | 0.007 | -8.543 | 0.000 |
| *SYT13* | 6.926 | 0.000 | 5.584 | 0.002 |
| *TAF1* | 2.622 | 0.055 | 2.580 | 0.058 |
| *TAGLN* | -1.540 | 0.046 | -1.931 | 0.015 |
| *TARS2* | 3.210 | 0.035 | 3.080 | 0.042 |
| *TAS2R40* | 1.272 | 0.077 | 1.519 | 0.038 |
| *TBCE* | 1.834 | 0.041 | 2.014 | 0.027 |
| *TBL1Y* | -1.916 | 0.035 | -1.732 | 0.054 |
| *TCF4* | 2.514 | 0.022 | 2.315 | 0.033 |
| *TEFM* | -1.997 | 0.094 | -3.413 | 0.008 |
| *TERF2IP* | -1.620 | 0.037 | -1.634 | 0.036 |
| *TESC* | -5.858 | 0.000 | -5.330 | 0.000 |
| *TEX10* | -2.273 | 0.039 | -2.312 | 0.036 |
| *TF* | -2.813 | 0.005 | -4.064 | 0.000 |
| *TFAP4* | -2.896 | 0.020 | -3.408 | 0.008 |
| *TFB1M* | -2.403 | 0.005 | -1.694 | 0.038 |
| *TFF1* | 3.443 | 0.000 | 2.974 | 0.000 |
| *TFF3* | 3.555 | 0.010 | 2.816 | 0.034 |
| *TFPI* | -4.674 | 0.002 | -4.098 | 0.005 |
| *TFRC* | 2.801 | 0.006 | 1.891 | 0.051 |
| *TGFB1I1* | -2.968 | 0.024 | -2.650 | 0.041 |
| *TGFBR2* | -4.572 | 0.004 | -6.776 | 0.000 |
| *THBS3* | 1.952 | 0.079 | 3.869 | 0.002 |
| *THC2497493* | -2.898 | 0.043 | -2.354 | 0.093 |
| *THC2507047* | 7.695 | 0.000 | 9.125 | 0.000 |
| *THC2537043* | -2.171 | 0.055 | -2.158 | 0.056 |
| *THC2538174* | 4.375 | 0.001 | 5.970 | 0.000 |
| *THC2581513* | 2.679 | 0.064 | 3.025 | 0.039 |
| *THC2586088* | -3.838 | 0.006 | -3.588 | 0.009 |
| *THC2588995* | -1.571 | 0.074 | -1.454 | 0.097 |
| *THC2670954* | -1.884 | 0.005 | -1.266 | 0.046 |
| *THC2686816* | -1.705 | 0.036 | -1.596 | 0.048 |
| *THC2698991* | -2.182 | 0.035 | -1.805 | 0.074 |
| *THC2769306* | -1.296 | 0.051 | -1.377 | 0.039 |
| *THC2772172* | 2.612 | 0.013 | 1.896 | 0.060 |
| *THEMIS* | -1.695 | 0.039 | -1.683 | 0.040 |
| *THOC2* | 2.161 | 0.070 | 2.677 | 0.028 |
| *THSD4* | -3.848 | 0.010 | -2.455 | 0.081 |
| *THY1* | 1.768 | 0.063 | 1.934 | 0.044 |
| *TIFA* | -1.538 | 0.093 | -1.626 | 0.077 |
| *TIMM17B* | 1.341 | 0.058 | 1.162 | 0.096 |
| *TIMM8A* | -2.609 | 0.052 | -3.880 | 0.007 |
| *TIPRL* | 1.479 | 0.064 | 1.654 | 0.041 |
| *TJP3* | 3.967 | 0.033 | 3.254 | 0.074 |
| *TK1* | 3.222 | 0.011 | 4.367 | 0.001 |
| *TK2* | -2.400 | 0.069 | -3.455 | 0.013 |
| *TLR6* | -2.105 | 0.018 | -1.508 | 0.077 |
| *TMEM108* | -1.367 | 0.053 | -1.356 | 0.055 |
| *TMEM126A* | 3.323 | 0.008 | 2.281 | 0.054 |
| *TMEM126B* | 2.007 | 0.019 | 1.455 | 0.078 |
| *TMEM139* | -4.105 | 0.021 | -4.279 | 0.017 |

| | | | | |
|---|---|---|---|---|
| *TMEM14A* | 3.295 | 0.061 | 3.701 | 0.037 |
| *TMEM255A* | -1.858 | 0.023 | -2.032 | 0.015 |
| *TMEM41A* | 2.166 | 0.073 | 2.701 | 0.029 |
| *TMOD4* | 1.623 | 0.097 | 1.937 | 0.052 |
| *TMPRSS6* | 1.963 | 0.095 | 2.206 | 0.064 |
| *TNFAIP1* | 1.563 | 0.047 | 1.721 | 0.030 |
| *TNPO1* | 3.473 | 0.019 | 2.421 | 0.089 |
| *TNS4* | -3.075 | 0.030 | -3.883 | 0.008 |
| *TOP2A* | 3.758 | 0.021 | 4.072 | 0.013 |
| *TOX3* | 2.108 | 0.080 | 2.534 | 0.039 |
| *TP53INP1* | 5.120 | 0.002 | 4.508 | 0.006 |
| *TPBG* | 1.617 | 0.055 | 1.499 | 0.073 |
| *TPK1* | -4.476 | 0.000 | -4.733 | 0.000 |
| *TPM3* | 2.402 | 0.004 | 2.288 | 0.006 |
| *TPPP2* | -2.198 | 0.005 | -1.471 | 0.046 |
| *TPR* | 2.560 | 0.006 | 2.849 | 0.003 |
| *TPRXL* | -2.253 | 0.017 | -3.155 | 0.002 |
| *TRAF3IP2* | -2.978 | 0.027 | -2.374 | 0.070 |
| *TRAPPC9* | 2.049 | 0.071 | 2.597 | 0.026 |
| *TRIB3* | 2.816 | 0.021 | 2.205 | 0.062 |
| *TRIM11* | 1.557 | 0.099 | 2.073 | 0.033 |
| *TRIM29* | -4.741 | 0.009 | -5.106 | 0.006 |
| *TRIM52-AS1* | -1.654 | 0.055 | -1.747 | 0.044 |
| *TRIM67* | -1.893 | 0.023 | -1.449 | 0.072 |
| *TRMT1* | -2.916 | 0.095 | -4.269 | 0.019 |
| *TSPAN31* | -1.977 | 0.094 | -2.079 | 0.079 |
| *TSPAN7* | -2.183 | 0.015 | -1.688 | 0.050 |
| *TSTA3* | 1.877 | 0.088 | 2.998 | 0.011 |
| *TTC37* | 1.481 | 0.086 | 1.908 | 0.031 |
| *TTLL11* | -3.704 | 0.001 | -4.655 | 0.000 |
| *TTLL4* | -2.920 | 0.023 | -3.766 | 0.005 |
| *TTPAL* | 4.122 | 0.017 | 4.200 | 0.015 |
| *TTYH1* | -3.869 | 0.001 | -3.710 | 0.002 |
| *TUBB3* | 3.975 | 0.007 | 5.106 | 0.001 |
| *TUBE1* | -3.212 | 0.042 | -4.450 | 0.007 |
| *TWSG1* | 1.524 | 0.072 | 1.574 | 0.064 |
| *TXNDC8* | 1.415 | 0.016 | 1.283 | 0.026 |
| *TXNIP* | -1.607 | 0.020 | -1.252 | 0.063 |
| *TXNL4A* | 1.945 | 0.007 | 1.173 | 0.081 |
| *TYMS* | 3.205 | 0.024 | 3.707 | 0.011 |
| *UBE2C* | 3.561 | 0.015 | 3.143 | 0.029 |
| *UBE2Q2P1* | 1.863 | 0.081 | 2.893 | 0.010 |
| *UBE2S* | 2.103 | 0.087 | 2.044 | 0.095 |
| *UBE2T* | 4.658 | 0.000 | 3.169 | 0.006 |
| *UBXN4* | 2.631 | 0.022 | 2.902 | 0.024 |
| *UCP3* | 2.004 | 0.032 | 1.878 | 0.042 |
| *UGDH* | 2.085 | 0.006 | 2.250 | 0.004 |
| *UGT2B15* | -1.703 | 0.099 | -1.774 | 0.087 |
| *UHMK1* | 2.880 | 0.006 | 3.274 | 0.002 |
| *UHRF1* | 2.739 | 0.076 | 2.719 | 0.078 |
| *ULK4P3* | -2.751 | 0.047 | -2.974 | 0.033 |
| *UNC13B* | 2.589 | 0.092 | 2.975 | 0.056 |
| *UNC5B* | 2.209 | 0.030 | 2.390 | 0.020 |
| *USPL1* | -2.127 | 0.095 | -2.701 | 0.039 |
| *UTP3* | 2.093 | 0.077 | 2.403 | 0.045 |

| | | | | |
|---|---|---|---|---|
| *UTP6* | 2.630 | 0.021 | 2.153 | 0.053 |
| *UTRN* | -1.656 | 0.017 | -2.708 | 0.000 |
| *VAC14-AS1* | 2.465 | 0.017 | 2.201 | 0.031 |
| *VAT1L* | -1.313 | 0.089 | -1.267 | 0.099 |
| *VCAN* | 2.566 | 0.054 | 3.514 | 0.012 |
| *VCP* | 1.581 | 0.021 | 1.183 | 0.075 |
| *VCPIP1* | 3.145 | 0.022 | 4.462 | 0.002 |
| *VDAC3* | 4.073 | 0.006 | 3.133 | 0.026 |
| *VEGFA* | 2.816 | 0.059 | 2.754 | 0.064 |
| *VEPH1* | -1.826 | 0.029 | -1.374 | 0.089 |
| *VGLL1* | -3.038 | 0.007 | -1.746 | 0.098 |
| *VPS13D* | -2.018 | 0.017 | -1.608 | 0.051 |
| *VPS37A* | 2.443 | 0.009 | 2.784 | 0.004 |
| *VPS37B* | -2.630 | 0.085 | -2.930 | 0.057 |
| *VPS51* | -1.657 | 0.082 | -2.715 | 0.008 |
| *VPS72* | 1.146 | 0.076 | 1.317 | 0.044 |
| *VSIG10L* | -1.954 | 0.024 | -2.356 | 0.008 |
| *VSNL1* | -2.467 | 0.021 | -2.081 | 0.047 |
| *WBP1* | 3.537 | 0.053 | 3.729 | 0.042 |
| *WDHD1* | 3.510 | 0.015 | 2.565 | 0.065 |
| *WDR35* | -2.463 | 0.092 | -2.845 | 0.055 |
| *WDR86* | -2.068 | 0.069 | -2.230 | 0.051 |
| *WDR91* | 3.224 | 0.067 | 2.998 | 0.086 |
| *WIF1* | -4.287 | 0.003 | -3.945 | 0.005 |
| *WIPI1* | 2.957 | 0.024 | 3.228 | 0.015 |
| *WNT6* | -2.355 | 0.044 | -2.215 | 0.057 |
| *WNT9B* | -1.537 | 0.050 | -1.545 | 0.049 |
| *WSB2* | 1.041 | 0.072 | 1.224 | 0.037 |
| *WWP1* | 2.420 | 0.013 | 2.172 | 0.023 |
| *XAB2* | 3.464 | 0.071 | 3.639 | 0.059 |
| *XIRP2* | -1.450 | 0.033 | -1.342 | 0.046 |
| *XK* | -1.487 | 0.082 | -1.577 | 0.067 |
| *YBX3* | -2.204 | 0.001 | -1.316 | 0.027 |
| *YWHAZ* | 0.876 | 0.091 | 0.917 | 0.078 |
| *ZBTB49* | -2.534 | 0.002 | -2.482 | 0.003 |
| *ZBTB7C* | 2.772 | 0.017 | 3.213 | 0.007 |
| *ZC3H14* | 1.648 | 0.034 | 1.880 | 0.018 |
| *ZC4H2* | -2.284 | 0.075 | -2.917 | 0.027 |
| *ZDHHC16* | 2.337 | 0.079 | 2.517 | 0.060 |
| *ZFP36L2* | -2.210 | 0.066 | -2.515 | 0.039 |
| *ZG16B* | 2.414 | 0.006 | 3.100 | 0.001 |
| *ZKSCAN2* | 2.270 | 0.045 | 3.318 | 0.006 |
| *ZNF285* | -2.784 | 0.022 | -3.377 | 0.007 |
| *ZNF322* | 2.353 | 0.008 | 2.217 | 0.011 |
| *ZNF350* | 2.315 | 0.069 | 2.277 | 0.073 |
| *ZNF358* | -1.183 | 0.098 | -2.066 | 0.007 |
| *ZNF442* | -1.604 | 0.063 | -1.586 | 0.096 |
| *ZNF470* | 2.077 | 0.068 | 2.480 | 0.032 |
| *ZNF471* | -1.806 | 0.071 | -1.687 | 0.089 |
| *ZNF501* | -3.222 | 0.002 | -2.238 | 0.022 |
| *ZNF502* | -4.927 | 0.001 | -4.157 | 0.004 |
| *ZNF506* | -2.719 | 0.014 | -1.824 | 0.084 |
| *ZNF521* | -5.390 | 0.002 | -2.820 | 0.073 |
| *ZNF548* | 2.158 | 0.052 | 2.067 | 0.061 |
| *ZNF567* | 2.557 | 0.020 | 2.136 | 0.047 |

| | | | | |
|---|---|---|---|---|
| *ZNF586* | -2.931 | 0.051 | -2.469 | 0.095 |
| *ZNF627* | 2.602 | 0.019 | 1.894 | 0.075 |
| *ZNF649* | -1.912 | 0.026 | -2.173 | 0.013 |
| *ZNF699* | -1.603 | 0.079 | -2.091 | 0.026 |
| *ZNF705G* | -1.489 | 0.051 | -1.859 | 0.018 |
| *ZNF721* | 3.608 | 0.000 | 3.452 | 0.000 |
| *ZNF827* | -1.386 | 0.094 | -2.069 | 0.017 |
| *ZNF846* | -1.320 | 0.075 | -1.397 | 0.061 |
| *ZNFX1* | 4.348 | 0.007 | 3.272 | 0.035 |
| *ZNRF1* | -2.437 | 0.027 | -2.529 | 0.023 |
| *ZP1* | 1.296 | 0.048 | 1.098 | 0.089 |
| *ZSCAN9* | -2.540 | 0.005 | -3.341 | 0.001 |
| *ZWINT* | 5.104 | 0.000 | 4.458 | 0.001 |

**Table S2.** Gene-set enrichment analysis of cell migration in solid and trabecular structures

| Gene Set Name | Overlap | P-value | FDR q-value | Genes |
|---|---|---|---|---|
| GO_LOCOMOTION | 141/1984 | 2.15E-22 | 1.62E-19 | *AKT1; CAV1; AGT; LYN; PTGS2; DAB2; SPRY2; DRD2; ITGB1BP1; SMAD3; SFRP1; FADD; GJA1; TGFB1I1; MESP1; IGFBP5; KANK1; LGALS9; PDGFRA; FGF2; SEMA5A; COL3A1; APC; TGFBR2; THY1; PRKCQ; ITGAV; GLG1; HMGB2; CTHRC1; SOX10; PTPRO; ANXA1; RPS19; SEMA3B; SEMA6D; VEGFA; CCN1; EDN3; EMP2; KIT; NTRK3; GCNT2; GLIPR2; PTN; P2RY12; S100A9; F11R; ACTN4; CHN1; ASPM; COL1A1; KIF14 ; FN1; NCKAP1L; EPHB1; PRKX; LEF1; HDAC5; SRGAP2; TP53INP1; HOXA7; SOX8; HOXA2; TRIM11; POSTN; MAG; COL5A1; BMERB1; RACK1; CAV2; CCL5; GRB2; FERMT1; GRB14; UNC5B; PDGFA; BST2; PAK5; EZR ; HSPB1; DNAJA1; DUSP1; GSTP1; ARRDC3; SCG2; TNFAIP1; KANK2; APOD; PADI2; CYP7B1; APCDD1; FAP; PLA2G6; CCL21; PIK3CG; TF; SASH1; S100A7; GLUL; BVES; LGR6; DCDC2; PPM1F; RGN; VEGFD; ROPN1; RRAS; GAPDHS; SGK1; MYLK; PKN3; MMP1; VPS37B; ADAM15; PRSS3; DOCK4; SEPTIN7; PARD6B; TUBB3; NFIB; FOLR1; JCHAIN; COL1A2; DNAH5; DNAH1; EGR2; CCL28; LAMA3; PODXL2; MATN2; AMOTL1; S100A14; FUT8; IGLL1; SAA4; CAVIN1; S100P; SLC16A3; ALCAM; ZNF502* |
| GO_LAMELLIPODIUM | 18/194 | 0.0000184 | 0.000263 | *ITGAV; APC; FAP; AMOTL1; SRGAP2; CTNND1; SLC39A6; HCLS1; STX2; PTPRO; MYLK; TESC; HAX ; TUBB3; ITGB1BP1; PTPN13; RAPH1; ABLIM1* |

**Table S3.** Genes expressed in solid and trabecular structures but not in tubular and alveolar structures of breast cancer (p-value < 0.05)

| Gene | Solid structures | | Trabecular structures | | Association with cell migration | |
|---|---|---|---|---|---|---|
| | LogFC | P-value | LogFC | P-value | Reference | Link |
| *AACS* | 2.791 | 0.038 | 3.038 | 0.026 | No data | |
| *ABLIM1* | -1.969 | 0.035 | -2.498 | 0.010 | Narahara et al, J Cell Physiol. 2018 | https://doi.org/10.1002/jcp.26605 |
| *ADAMTS16* | 1.968 | 0.030 | 1.849 | 0.040 | Surridge et al., Matrix Biol. 2009 | https://doi.org/10.1016/j.matbio.2009.07.001 |
| *AK130224* | -1.889 | 0.039 | -1.908 | 0.037 | No data | |
| *ALDH7A1* | -2.031 | 0.035 | -2.932 | 0.004 | Elsalem et al., EJC 2016 | https://doi.org/10.1016/S0959-8049(16)61361-8 |
| *ALG5* | 2.529 | 0.008 | 2.014 | 0.029 | No data | |
| *ALYREF* | 2.728 | 0.045 | 3.047 | 0.027 | Saito et al., J Cancer Res Clin Oncol. 2013 | https://doi.org/10.1007/s00432-012-1361-5 |
| *AMOTL1* | -2.854 | 0.031 | -3.137 | 0.019 | Couderc et al., Neoplasia 2016 | https://doi.org/10.1016/j.neo.2015.11.010 |
| *APOA1BP* | 3.700 | 0.029 | 3.293 | 0.049 | No data | |
| *ASCC3* | 3.359 | 0.037 | 3.403 | 0.035 | No data | |
| *BRMS1L* | -2.203 | 0.035 | -2.056 | 0.048 | Gong et al., Nat Commun. 2014 | https://doi.org/10.1038/ncomms6406 |
| *BU963192* | 3.430 | 0.027 | 3.839 | 0.015 | No data | |
| *BVES* | -2.099 | 0.018 | -2.015 | 0.023 | Benesh et al., Mol Biol Cell. 2013 | https://doi.org/10.1091/mbc.E12-07-0539 |
| *CAV1* | -3.196 | 0.046 | -3.204 | 0.045 | Grande-García et al., J Cell Biol. 2007 | https://doi.org/10.1083/jcb.200701006 |
| *CEP78* | 1.569 | 0.038 | 1.564 | 0.038 | No data | |
| *CHST2* | -2.771 | 0.011 | -2.788 | 0.011 | No data | |
| *CLUAP1* | 2.909 | 0.022 | 3.510 | 0.008 | Beyer et al., Mol Cell Proteomics. 2018 | https://doi.org/10.1074/mcp.RA117.000487 |
| *CNIH4* | 1.758 | 0.015 | 1.384 | 0.048 | Mishra et al., Oncogene 2019 | https://doi.org/10.1038/s41388-019-0845-z |
| *CTIF* | 2.482 | 0.043 | 2.559 | 0.038 | No data | |
| *CTNND1* | -2.277 | 0.020 | -2.538 | 0.011 | Tang et al., J Exp Clin Cancer Res. 2016 | https://doi.org/10.1186/s13046-016-0344-9 |
| *DDI2* | -3.700 | 0.026 | -3.541 | 0.032 | No data | |
| *DNAH1* | -1.401 | 0.048 | -1.846 | 0.012 | Milosevic et al., BMC Cancer. 2013 | https://doi.org/10.1186/1471-2407-13-473 |
| *DNAJC22* | 2.954 | 0.008 | 2.224 | 0.037 | No data | |
| *ENST00000299512* | -1.620 | 0.025 | -1.898 | 0.011 | No data | |
| *ENST00000334163* | 2.033 | 0.025 | 2.236 | 0.015 | No data | |
| *ENST00000417510* | -1.992 | 0.018 | -2.909 | 0.001 | No data | |
| *ENST00000429552* | -1.202 | 0.047 | -1.193 | 0.048 | No data | |
| *ENST00000433051* | -1.875 | 0.031 | -1.897 | 0.030 | No data | |
| *ENST00000455206* | -1.216 | 0.036 | -1.276 | 0.029 | No data | |
| *ENST00000457863* | 2.409 | 0.014 | 2.264 | 0.019 | No data | |
| *ENST00000502684* | -1.756 | 0.022 | -1.698 | 0.026 | No data | |

| | | | | | | |
|---|---|---|---|---|---|---|
| *ENST00000506710* | -2.078 | 0.008 | -1.494 | 0.043 | No data | |
| *ENST00000527434* | 2.689 | 0.003 | 2.114 | 0.015 | No data | |
| *ENST00000596267* | 2.483 | 0.021 | 2.502 | 0.020 | No data | |
| *ETFDH* | 2.367 | 0.048 | 3.473 | 0.006 | No data | |
| *EXOC1* | 1.947 | 0.035 | 3.249 | 0.001 | Andersen & Yeaman, Mol Biol Cell. 2010 | https://doi.org/10.1091/mbc.E09-06-0459 |
| *EZR* | 1.993 | 0.036 | 1.918 | 0.043 | Arpin et al., Cell Adh Migr. 2011 | https://doi.org/10.4161/cam.5.2.15081 |
| *F13A1* | 3.636 | 0.019 | 4.639 | 0.004 | Dardik et al., Cell Physiol Biochem. 2007 | https://doi.org/10.1159/000099199 |
| *FAM109A* | -3.613 | 0.015 | -3.259 | 0.026 | No data | |
| *FBXO28* | 2.370 | 0.015 | 1.881 | 0.047 | No data | |
| *FCER1A* | -3.174 | 0.026 | -3.224 | 0.024 | No data | |
| *FDCSP* | -3.715 | 0.030 | -3.687 | 0.031 | Wang et al., Oncol Rep. 2010 | https://www.ncbi.nlm.nih.gov/pubmed/20811673 |
| *GLG1* | -1.616 | 0.040 | -1.737 | 0.029 | Planche et al., Cancer Res. 2012 | https://doi.org/10.1158/1538-7445.AM2012-3245 |
| *GPR75* | -2.847 | 0.048 | -4.330 | 0.005 | Fan & Roman, Circ Res. 2017 | https://doi.org/10.1161/CIRCRESAHA.117.311022 |
| *HDAC3* | 2.046 | 0.016 | 1.978 | 0.020 | He et al., Int J Oncol. 2018 | https://doi.org/10.3892/ijo.2018.4284 |
| *HMGB3P1* | 2.657 | 0.009 | 2.486 | 0.013 | No data | |
| *IL13RA1* | 1.883 | 0.037 | 2.077 | 0.023 | Moretti et al., Medical Express 2015 | https://doi.org/10.5935/MedicalExpress.2015.05.05 |
| *IQSEC3* | -1.683 | 0.047 | -2.189 | 0.013 | No data | |
| *KBTBD6* | -2.913 | 0.014 | -2.251 | 0.049 | No data | |
| *KCNIP3* | -1.493 | 0.012 | -1.398 | 0.018 | No data | |
| *KLHL14* | 1.386 | 0.034 | 1.870 | 0.006 | Di Lollo et al., Sci Rep. 2020 | https://doi.org/10.1038/s41598-020-61017-y |
| *KPNB1* | 1.769 | 0.032 | 1.760 | 0.033 | Stelma & Leaner, Oncotarget. 2017 | https://doi.org/10.18632/oncotarget.15834 |
| *L3HYPDH* | 2.652 | 0.040 | 2.902 | 0.027 | No data | |
| *LINC00240* | -2.121 | 0.029 | -2.374 | 0.016 | No data | |
| *LINC00865* | -2.723 | 0.018 | -2.708 | 0.019 | No data | |
| *LIPG* | -1.589 | 0.022 | -1.625 | 0.020 | Lo et al., eLife. 2018 | https://doi.org/10.7554/eLife.31334 |
| *LOC100506314* | -1.646 | 0.046 | -2.120 | 0.013 | No data | |
| *LOC100506411* | 2.372 | 0.016 | 1.905 | 0.047 | No data | |
| *LOC100506827* | -1.602 | 0.026 | -1.662 | 0.022 | No data | |
| *LOC101927322* | -1.802 | 0.046 | -2.040 | 0.026 | No data | |
| *LOC102723882* | -1.863 | 0.035 | -1.716 | 0.050 | No data | |
| *LOC643454* | 1.896 | 0.024 | 1.664 | 0.044 | No data | |
| *LOC643733* | -2.994 | 0.008 | -2.813 | 0.011 | No data | |
| *LRRN4CL* | 3.000 | 0.041 | 3.525 | 0.019 | No data | |
| *LUC7L2* | -2.566 | 0.011 | -2.435 | 0.015 | No data | |
| *MASTL* | 3.283 | 0.049 | 3.579 | 0.034 | Rogers et al., Oncogene. 2018 | https://doi.org/10.1038/s41388-018-0295-z |
| *METTL10* | 1.855 | 0.042 | 1.864 | 0.042 | No data | |

| | | | | | | |
|---|---|---|---|---|---|---|
| *MFI2* | -2.873 | 0.023 | -3.043 | 0.017 | Yin et al., Oncol Rep. 2016 | https://doi.org/10.3892/or.2016.5013 |
| *MIR3916* | 2.218 | 0.033 | 2.143 | 0.039 | No data | |
| *MIR503HG* | 1.585 | 0.040 | 1.762 | 0.024 | No data | |
| *MRPL55* | 1.307 | 0.036 | 1.632 | 0.011 | No data | |
| *MTF2* | 2.787 | 0.033 | 2.715 | 0.037 | No data | |
| *MUTYH* | 2.956 | 0.032 | 3.573 | 0.012 | Gao et al., Onco Targets Ther. 2018 | https://doi.org/10.2147/OTT.S174223 |
| *MVB12B* | -2.918 | 0.050 | -3.041 | 0.042 | No data | |
| *NAA10* | 2.254 | 0.049 | 2.996 | 0.012 | No data | |
| *NCKAP1L* | -1.724 | 0.027 | -1.737 | 0.026 | Afzal et al., J Am Heart Assoc. 2016 | https://doi.org/10.1161/JAHA.116.004629 |
| *NEIL1* | -2.997 | 0.043 | -3.746 | 0.014 | No data | |
| *PLCH1* | -1.734 | 0.046 | -1.762 | 0.043 | No data | |
| *PRR19* | 2.068 | 0.023 | 2.054 | 0.024 | No data | |
| *PSCA* | 2.071 | 0.045 | 3.296 | 0.003 | Wang et al., J Pathol. 2015 | https://doi.org/10.1002/path.4582 |
| *RAD17* | -2.498 | 0.013 | -2.049 | 0.036 | No data | |
| *RBBP8* | 1.913 | 0.044 | 1.925 | 0.043 | No data | |
| *RCBTB1* | 4.310 | 0.009 | 3.084 | 0.050 | No data | |
| *RECQL* | 2.091 | 0.049 | 3.304 | 0.004 | No data | |
| *RFTN1* | 2.980 | 0.012 | 2.530 | 0.030 | No data | |
| *RPS18* | -1.289 | 0.045 | -1.281 | 0.046 | No data | |
| *SCAMP3* | 1.588 | 0.022 | 1.517 | 0.027 | No data | |
| *SLC16A3* | 2.729 | 0.043 | 3.918 | 0.006 | No data | |
| *SLC4A11* | -3.117 | 0.013 | -3.617 | 0.005 | No data | |
| *SLFN12* | -1.905 | 0.031 | -1.741 | 0.047 | No data | |
| *SMIM17* | 3.243 | 0.027 | 3.682 | 0.013 | No data | |
| *SNORD104* | 4.456 | 0.011 | 3.969 | 0.022 | No data | |
| *SPAG4* | 2.182 | 0.021 | 2.413 | 0.012 | Ji et al., Oncol Rep. 2018 | https://doi.org/10.3892/or.2018.6473 |
| *SPATA18* | -2.714 | 0.010 | -3.020 | 0.005 | Okuyama et al., Sci Rep. 2019 | https://doi.org/10.1038/s41598-019-39563-x |
| *TARS2* | 3.210 | 0.035 | 3.080 | 0.042 | No data | |
| *TERF2IP* | -1.620 | 0.037 | -1.634 | 0.036 | No data | |
| *TFAP4* | -2.896 | 0.020 | -3.408 | 0.008 | Huang et al., Dis Markers. 2019 | https://doi.org/10.1155/2019/7129214 |
| *TFB1M* | -2.403 | 0.005 | -1.694 | 0.038 | No data | |
| *TFF3* | 3.555 | 0.010 | 2.816 | 0.034 | Yusufu et al., Int J Oncol. 2019 | https://doi.org/10.3892/ijo.2019.4854 |
| *TGFB1I1* | -2.968 | 0.024 | -2.650 | 0.041 | Gulvady et al., Mol Biol Cell. 2018 | https://doi.org/10.1091/mbc.E18-02-0092 |
| *THEMIS* | -1.695 | 0.039 | -1.683 | 0.040 | No data | |
| *TNFAIP1* | 1.563 | 0.047 | 1.721 | 0.030 | Xiao et al., EBioMedicine. 2020 | https://doi.org/10.1016/j.ebiom.2019.102603 |
| *TUBE1* | -3.212 | 0.042 | -4.450 | 0.007 | No data | |

| | | | | | | |
|---|---|---|---|---|---|---|
| *TXNDC8* | 1.415 | 0.016 | 1.283 | 0.026 | No data | |
| *ULK4P3* | -2.751 | 0.047 | -2.974 | 0.033 | No data | |
| *UNC5B* | 2.209 | 0.030 | 2.390 | 0.020 | Kong et al., BMC Cancer. 2016 | https://doi.org/10.1186/s12885-016-2922-9 |
| *WWP1* | 2.420 | 0.013 | 2.172 | 0.023 | Goto et al., Br J Cancer. 2016 | https://doi.org/10.1038/bjc.2016.95 |
| *ZKSCAN2* | 2.270 | 0.045 | 3.318 | 0.006 | Kawahara et al., Oncotarget. 2016 | https://doi.org/10.18632/oncotarget.10679 |
| *ZNF285* | -2.784 | 0.022 | -3.377 | 0.007 | No data | |
| *ZNF567* | 2.557 | 0.020 | 2.136 | 0.047 | No data | |
| *ZNRF1* | -2.437 | 0.027 | -2.529 | 0.023 | No data | |

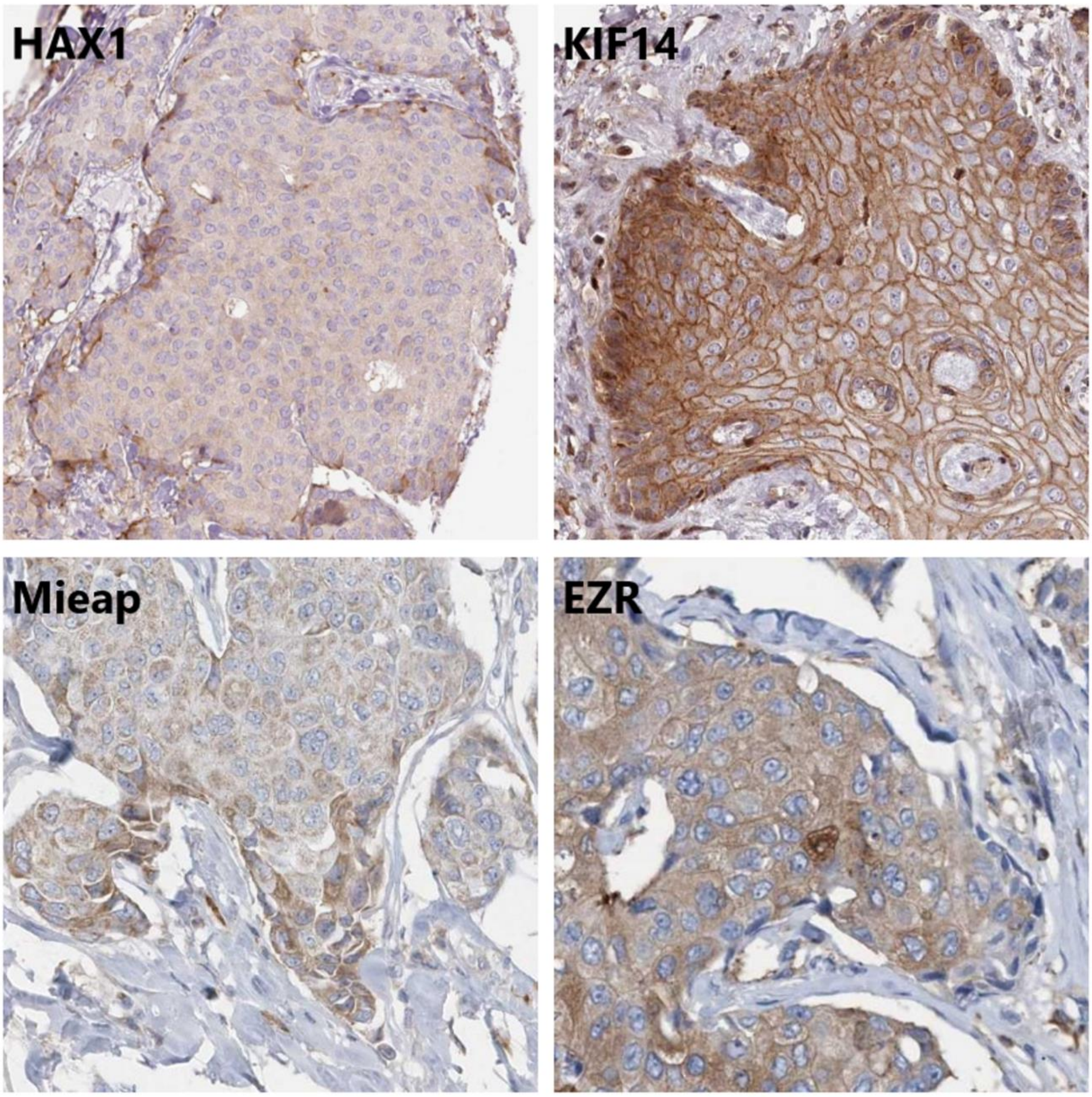


**Figure S1.** Expression of HAX1, KIF14, Mieap and EZR proteins in breast cancer (according to the Human Protein Atlas). HAX1 is predominantly expressed at the periphery of solid structure. Positive expression of KIF14 and Mieap and negative expression of EZR are observed at the tips of the solid structures.

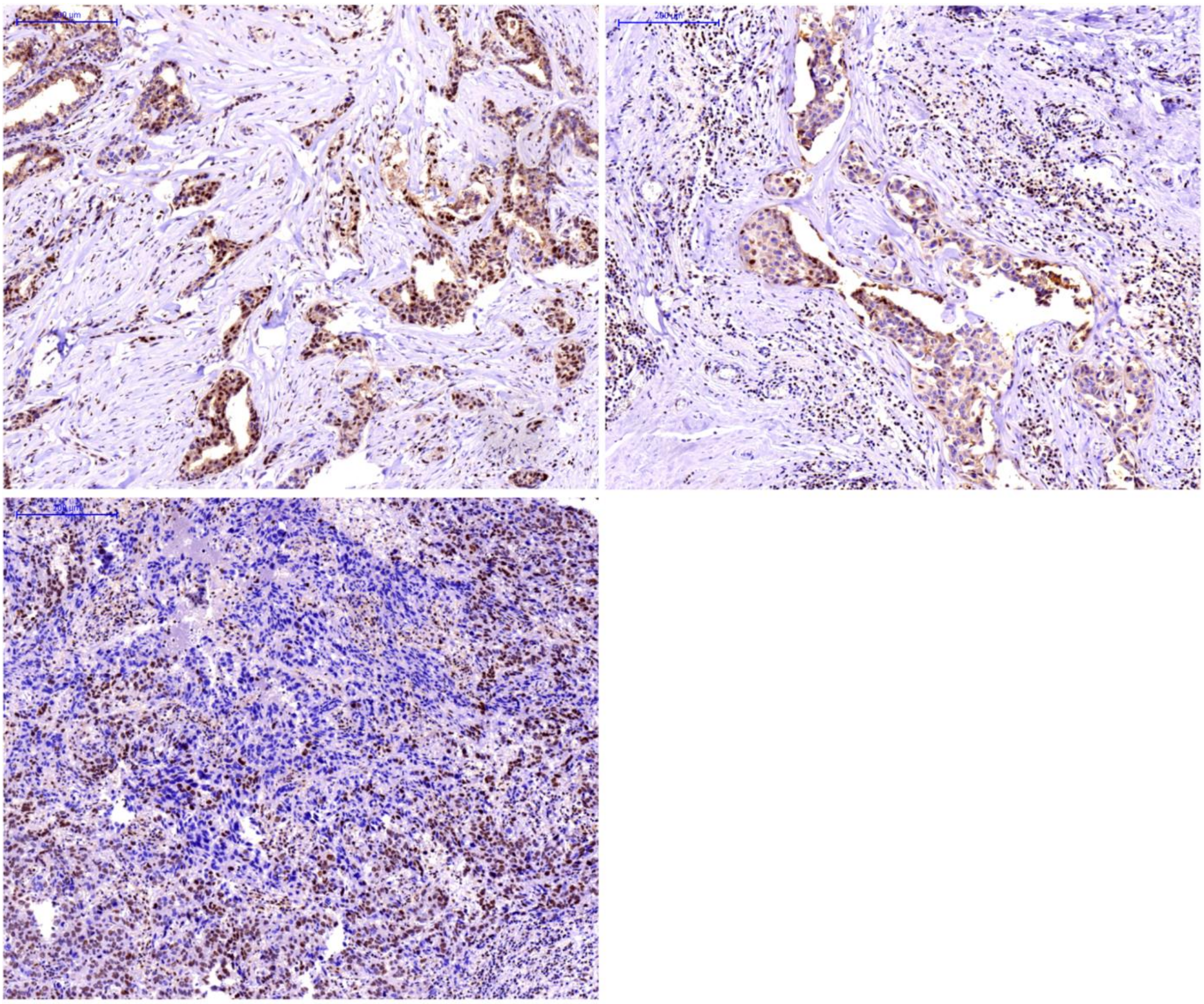

**Figure S2.** Immunohistochemical staining for KIF14 in breast cancers. 200x magnification.

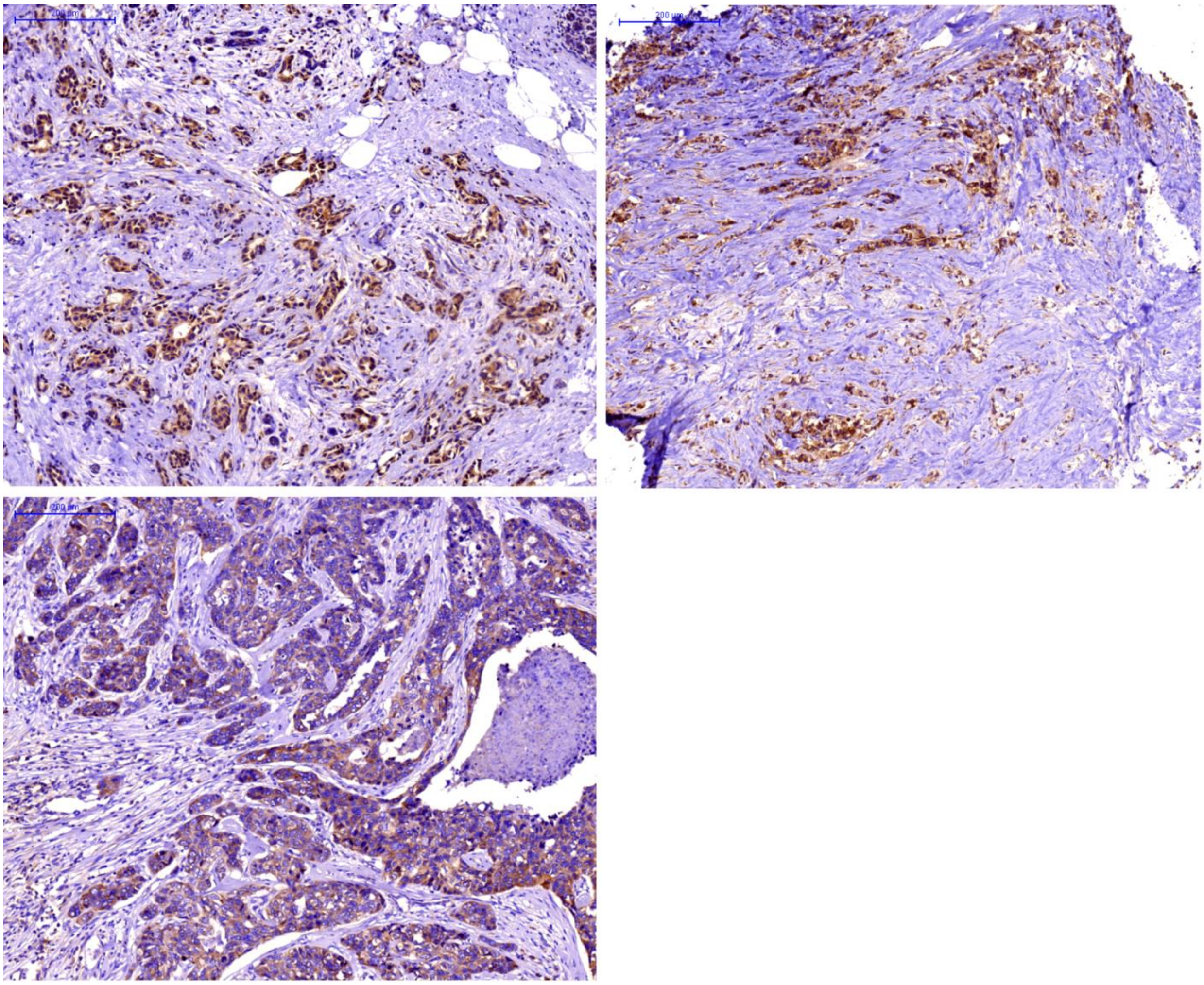

**Figure S3.** Immunohistochemical staining for Mieap in breast cancers. 200x magnification.

.

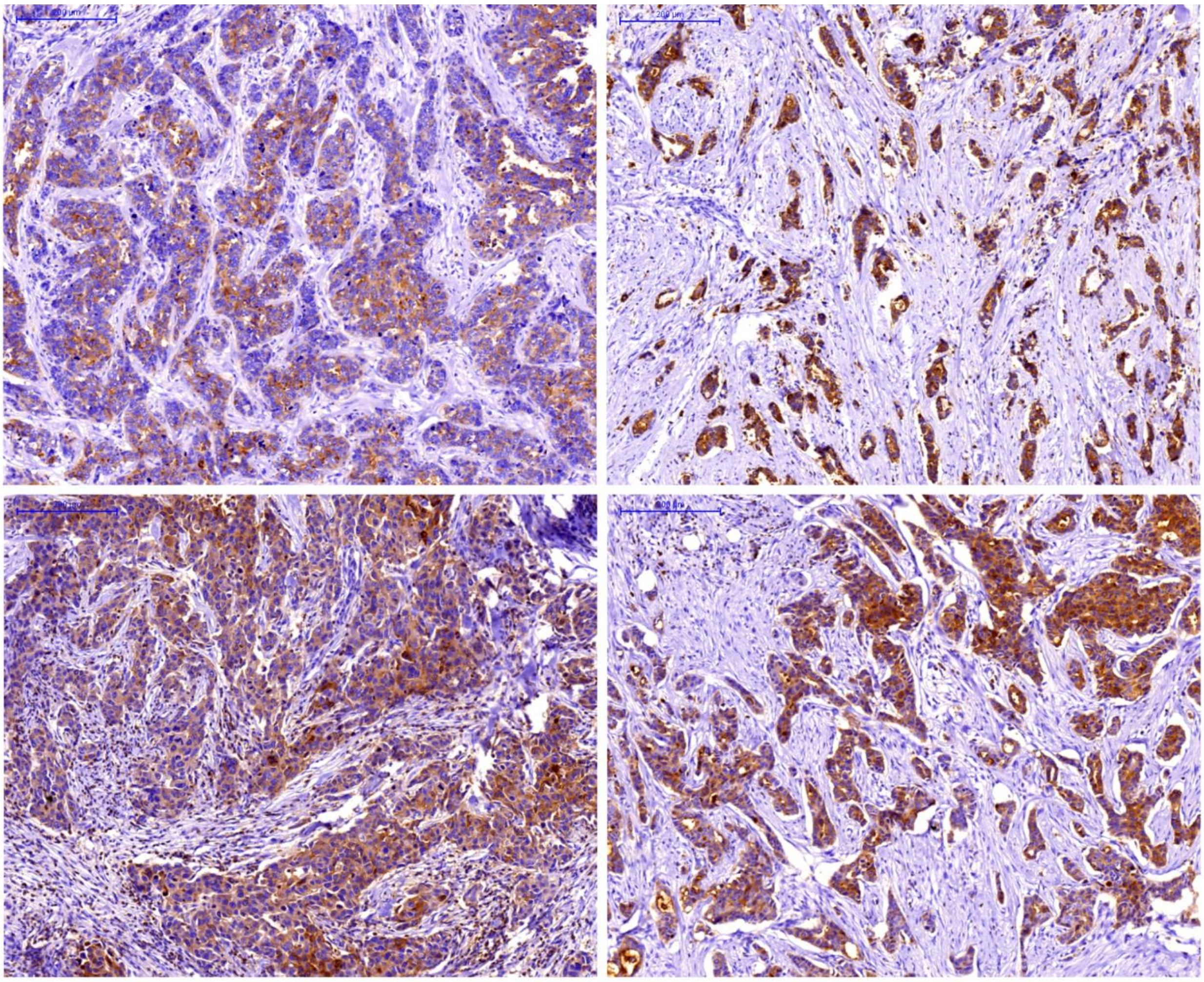

**Figure S4.** Immunohistochemical staining for EZR in breast cancers. 200x magnification.

**Table S4.** The frequency of nuclear expression of KIF14 in different morphological structures of breast tumors

| | Morphological structures | Number of cases (%) |
|---|---|---|
| 1 | Single tumor cells | 27/69 (39.1)<br>$p_2$=0.05<br>$p_3$=0.02 |
| 2 | At the tips of torpedo-like sprouts associated with solid structures | 26/48 (54.2) |
| 3 | At the tips of torpedo-like structures (separated from solid structures) | 21/35 (60.0) |
| 4 | At the periphery of solid structures | 37/71 (52.1) |
| 5 | At the periphery of alveolar structures | 35/67 (52.2) |
| 6 | At the periphery of trabecular structures | 33/36 (91.7)<br>$p_1$=0.0000<br>$p_2$=0.0001<br>$p_3$=0.0008<br>$p_4$=0.0000<br>$p_5$=0.0000 |
| 7 | At the periphery of tubular structures | 14/16 (87.5)<br>$p_1$=0.0002<br>$p_2$=0.007<br>$p_3$=0.02<br>$p_4$=0.004<br>$p_5$=0.004 |

$p_{1\text{-}5,}$ comparison between different structures (1-5); Chi-square test

**Table S5.** The frequency of cytoplasmic expression of Mieap in different morphological structures of breast tumors

| | Morphological structures | Number of cases (%) |
|---|---|---|
| 1 | Single tumor cells | 30/70 (42.8) |
| 2 | At the tips of torpedo-like sprouts associated with solid structures | 24/49 (48.9) |
| 3 | At the tips of torpedo-like structures (separated from solid structures) | 20/36 (55.5) |
| 4 | At the periphery of solid structures | 29/72 (40.2) |
| 5 | At the periphery of alveolar structures | 30/68 (44.1) |
| 6 | At the periphery of trabecular structures | 29/39 (74.3)<br>$p_1$=0.0009<br>$p_2$=0.008<br>$p_3$=0.05<br>$p_4$=0.003<br>$p_5$=0.01 |
| 7 | At the periphery of tubular structures | 10/16 (62.5)<br>$p_4$=0.04 |

$p_{1\text{-}5,}$ comparison between different structures (1-5); Chi-square test

**Table S6.** The frequency of negative cytoplasmic expression of EZR in different morphological structures of breast tumors

| | Morphological structures | Number of cases (%) |
|---|---|---|
| 1 | Single tumor cells | 33/66 (50.0) |
| 2 | At the tips of torpedo-like sprouts associated with solid structures | 25/45 (55.5) |
| 3 | At the tips of torpedo-like structures (separated from solid structures) | 19/34 (55.9) |
| 4 | At the periphery of solid structures | 36/69 (52.2) |
| 5 | At the periphery of alveolar structures | 19/35 (54.3) |
| 6 | At the periphery of trabecular structures | 28/34 (82.3)<br>$p_1$=0.0009<br>$p_2$=0.007<br>$p_3$=0.01<br>$p_4$=0.002<br>$p_5$=0.006 |
| 7 | Tubular structures | 15/17 (88.2)<br>$p_1$=0.002<br>$p_2$=0.009<br>$p_3$=0.01<br>$p_4$=0.003<br>$p_5$=0.007 |

$p_{1-5,}$ comparison between different structures (1-5); Chi-square test

**Table S7.** Differentially expressed genes between KIF14-positive and KIF14-negative cells located in torpedo-like structures ($p < 0.05$)

| Gene | P-value | LogFC |
|---|---|---|
| *SLITRK6* | 0.000 | -22.277 |
| *NHS* | 0.000 | 8.725 |
| *CNOT2-DT* | 0.000 | 7.941 |
| *CLIP4* | 0.001 | 7.223 |
| *LOC101928728* | 0.001 | 8.473 |
| *THBS4* | 0.002 | 6.420 |
| *DDHD1* | 0.002 | -5.906 |
| *LUM* | 0.002 | -5.989 |
| *GPRIN1* | 0.002 | 5.594 |
| *HIF1A-AS3* | 0.002 | 7.172 |
| *PIK3CB* | 0.003 | -4.657 |
| *UGT8* | 0.003 | 6.986 |
| *ARL13A* | 0.003 | 6.792 |
| *ATPAF2* | 0.003 | -5.752 |
| *LRRC26* | 0.004 | 5.290 |
| *GPR87* | 0.004 | 6.137 |
| *RBM12B* | 0.004 | -4.698 |
| *PRICKLE2* | 0.004 | -5.305 |
| *DENND5B* | 0.004 | -5.869 |
| *CROT* | 0.004 | -5.644 |
| *ZNF761* | 0.004 | -4.858 |
| *TMEM175* | 0.005 | -5.734 |
| *DCHS2* | 0.005 | 5.624 |
| *GTF2E1* | 0.005 | -5.567 |
| *EYA1* | 0.005 | -6.392 |
| *KIF21A* | 0.005 | -5.652 |
| *RALB* | 0.005 | -5.550 |
| *NEURL1* | 0.005 | 7.024 |
| *ARPC5L* | 0.005 | -5.536 |
| *C2orf66* | 0.006 | 7.442 |
| *ADGRG7* | 0.006 | 7.442 |
| *EPHB1* | 0.006 | 7.442 |
| *CPN1* | 0.006 | 7.442 |
| *MYADM* | 0.006 | -5.601 |
| *PLA2G7* | 0.006 | 6.576 |
| *CALD1* | 0.006 | -3.831 |
| *DPT* | 0.008 | 6.774 |
| *PLCL1* | 0.008 | -6.319 |
| *PSAPL1* | 0.008 | 6.957 |
| *TRIM38* | 0.008 | -5.336 |
| *PLA2G4F* | 0.008 | 4.146 |
| *LOC105369779* | 0.010 | 6.630 |
| *FAM102B* | 0.010 | 4.540 |
| *XKR5* | 0.010 | 7.064 |
| *PITRM1-AS1* | 0.010 | 7.064 |
| *BCL2A1* | 0.010 | 7.064 |
| *MAMDC2-AS1* | 0.010 | 6.423 |

| | | |
|---|---|---|
| *PLCB4* | 0.010 | -5.201 |
| *ZNF460* | 0.011 | -4.177 |
| *NAPB* | 0.011 | -5.202 |
| *MSANTD1* | 0.011 | 6.262 |
| *LEPROT* | 0.011 | -4.477 |
| *METTL6* | 0.012 | -5.210 |
| *THBS2* | 0.012 | -4.013 |
| *CYP4F8* | 0.012 | -5.747 |
| *C17orf58* | 0.013 | -5.114 |
| *ADAMTS7* | 0.013 | 6.692 |
| *DTNA* | 0.013 | -5.138 |
| *RBM48* | 0.013 | -5.334 |
| *DDX20* | 0.013 | -5.159 |
| *INPP5J* | 0.013 | -4.556 |
| *DLC1* | 0.013 | 5.076 |
| *USP46* | 0.014 | -4.618 |
| *LINC00635* | 0.014 | 6.831 |
| *TRPC7* | 0.014 | 6.831 |
| *LTA* | 0.014 | 6.831 |
| *KRTAP13-4* | 0.014 | 6.831 |
| *EPHB6* | 0.014 | 5.616 |
| *C1orf105* | 0.015 | 6.228 |
| *BMP2K* | 0.015 | -4.558 |
| *POT1* | 0.015 | -5.083 |
| *QDPR* | 0.015 | -5.517 |
| *VPS29* | 0.015 | -4.826 |
| *SNAPC1* | 0.015 | -5.149 |
| *PGAM5* | 0.015 | -5.070 |
| *SAP30L* | 0.015 | -4.447 |
| *IL1RN* | 0.015 | 6.171 |
| *CXorf56* | 0.016 | -5.047 |
| *DGAT1* | 0.016 | 3.952 |
| *ANTKMT* | 0.016 | -5.028 |
| *LIMD1* | 0.016 | -3.737 |
| *SEC61A2* | 0.016 | -5.088 |
| *ERCC4* | 0.017 | -5.131 |
| *TLL2* | 0.017 | 6.538 |
| *FCGBP* | 0.017 | -4.525 |
| *PVT1* | 0.017 | -5.537 |
| *SLC26A2* | 0.017 | -5.081 |
| *CINP* | 0.018 | -4.998 |
| *ASTN2* | 0.018 | -5.407 |
| *ST7L* | 0.018 | -4.985 |
| *LAP3* | 0.018 | -3.951 |
| *AIP* | 0.019 | 3.591 |
| *ZNF273* | 0.019 | -4.026 |
| *CDH6* | 0.019 | 6.472 |
| *ZNF726* | 0.019 | -5.149 |
| *CYP2U1* | 0.020 | 6.307 |
| *KIAA0930* | 0.020 | -4.112 |

| | | |
|---|---|---|
| *B4GALT6* | 0.020 | 6.091 |
| *AKR7A3* | 0.021 | -5.242 |
| *NFATC1* | 0.021 | -4.878 |
| *CAMP* | 0.021 | 6.402 |
| *EDN2* | 0.021 | 6.555 |
| *IVL* | 0.021 | 6.555 |
| *GAL3ST4* | 0.021 | 6.555 |
| *AGAP4* | 0.021 | 6.555 |
| *CCDC196* | 0.021 | 6.555 |
| *ADGRG3* | 0.021 | 6.555 |
| *B4GAT1* | 0.022 | 5.163 |
| *CDAN1* | 0.022 | 3.286 |
| *MIR3136* | 0.022 | 6.519 |
| *RAD51B* | 0.022 | -4.903 |
| *SLC49A3* | 0.022 | -4.943 |
| *CSGALNACT1* | 0.022 | -4.510 |
| *ASCC2* | 0.023 | -4.195 |
| *HMGN4* | 0.023 | -4.903 |
| *SNHG3* | 0.023 | -4.976 |
| *N4BP3* | 0.023 | 3.499 |
| *ATP6AP1L* | 0.023 | -3.818 |
| *MGAT5* | 0.023 | -3.534 |
| *STAM* | 0.023 | -4.845 |
| *CRY1* | 0.023 | -4.861 |
| *EEF1A2* | 0.024 | -5.047 |
| *CCDC61* | 0.024 | -4.833 |
| *MAB21L2* | 0.024 | -4.899 |
| *ATF3* | 0.024 | -4.911 |
| *TBC1D8B* | 0.024 | -5.028 |
| *NOS1AP* | 0.024 | -4.506 |
| *TMEM169* | 0.024 | 4.867 |
| *KIF1A* | 0.025 | 6.318 |
| *RGP1* | 0.025 | -4.088 |
| *NAPRT* | 0.026 | 5.682 |
| *NEDD1* | 0.027 | -5.206 |
| *MMP13* | 0.027 | -5.336 |
| *ALDH1A1* | 0.027 | 5.857 |
| *ZNF519* | 0.028 | -4.868 |
| *LCP1* | 0.028 | -4.780 |
| *SLC15A2* | 0.028 | -4.819 |
| *PARP12* | 0.029 | -4.126 |
| *EIF2B3* | 0.029 | -4.728 |
| *CREBL2* | 0.029 | -4.290 |
| *TMEM170B* | 0.029 | 4.354 |
| *GLI4* | 0.029 | 4.170 |
| *EIF4ENIF1* | 0.029 | -4.416 |
| *BET1* | 0.030 | -4.024 |
| *TMEM41B* | 0.030 | -3.824 |
| *TSBP1* | 0.030 | 5.596 |
| *RAB18* | 0.030 | -3.087 |

| | | |
|---|---|---|
| *ASB7* | 0.031 | -4.715 |
| *CLCA2* | 0.031 | 6.189 |
| *ACYP2* | 0.031 | 5.729 |
| *ZFHX4* | 0.031 | -4.859 |
| *NDUFAF1* | 0.031 | -4.700 |
| *SDHAF1* | 0.031 | -4.702 |
| *NAA10* | 0.031 | 5.233 |
| *RPL22L1* | 0.032 | -4.795 |
| *ZSCAN9* | 0.032 | -4.680 |
| *TLE6* | 0.033 | 4.771 |
| *VCX* | 0.033 | 5.545 |
| *ZRANB3* | 0.033 | -4.768 |
| *ENC1* | 0.033 | -3.147 |
| *VPS9D1* | 0.034 | 4.028 |
| *FGFR1* | 0.034 | 3.521 |
| *ZNF571-AS1* | 0.034 | -4.529 |
| *VRK1* | 0.034 | -4.665 |
| *GMPS* | 0.034 | -3.475 |
| *TUBGCP5* | 0.034 | -3.967 |
| *SCNM1* | 0.035 | -4.745 |
| *CRIPT* | 0.035 | -4.412 |
| *LY6G5B* | 0.035 | 5.756 |
| *CLDN19* | 0.035 | 6.216 |
| *PRR9* | 0.035 | 6.216 |
| *OTOL1* | 0.035 | 6.216 |
| *LINC02015* | 0.035 | 6.216 |
| *PDE6A* | 0.035 | 6.216 |
| *RS1* | 0.035 | 6.216 |
| *MROH5* | 0.035 | 6.216 |
| *SH2D4B* | 0.035 | 6.216 |
| *SLC10A2* | 0.035 | 6.216 |
| *KCNH5* | 0.035 | 6.216 |
| *GCNT3* | 0.035 | 6.216 |
| *LINC01255* | 0.035 | 6.216 |
| *PLA2G4C* | 0.035 | 6.216 |
| *TGIF2LY* | 0.035 | 6.216 |
| *TOP3B* | 0.035 | 6.216 |
| *ZSCAN22* | 0.035 | -4.682 |
| *B3GALT2* | 0.035 | -4.650 |
| *DFFA* | 0.036 | -4.141 |
| *IFT27* | 0.036 | -4.658 |
| *EDARADD* | 0.036 | -5.528 |
| *LINS1* | 0.037 | -4.166 |
| *SIRPA* | 0.037 | -4.636 |
| *IPMK* | 0.037 | -4.631 |
| *USP45* | 0.037 | -4.046 |
| *PLEKHG6* | 0.037 | 3.310 |
| *LINC01608* | 0.037 | 6.069 |
| *FAM86DP* | 0.039 | -4.769 |
| *RAB11FIP5* | 0.039 | -4.597 |

| | | |
|---|---|---|
| *COL2A1* | 0.039 | 5.579 |
| *ARVCF* | 0.039 | -3.402 |
| *SSH2* | 0.039 | -4.155 |
| *UNKL* | 0.039 | 2.608 |
| *LOC100129484* | 0.039 | -4.687 |
| *RGS1* | 0.040 | -4.659 |
| *FAM200A* | 0.040 | -4.575 |
| *NETO2* | 0.040 | 6.023 |
| *CAMK1D* | 0.040 | 4.303 |
| *CYP20A1* | 0.041 | -3.263 |
| *BBS5* | 0.041 | -4.579 |
| *KCNK5* | 0.041 | 6.011 |
| *ANXA3* | 0.041 | -4.977 |
| *PPA1* | 0.041 | -4.042 |
| *SCUBE3* | 0.041 | -4.977 |
| *PROS1* | 0.042 | 4.855 |
| *CTTNBP2* | 0.042 | -4.909 |
| *PPRC1* | 0.042 | -3.940 |
| *CSRNP2* | 0.042 | -3.233 |
| *SOCS6* | 0.042 | -3.746 |
| *GET1* | 0.042 | 4.264 |
| *KLK14* | 0.043 | -5.400 |
| *SLC9A2* | 0.043 | -5.031 |
| *NRAS* | 0.043 | -3.599 |
| *GALNT3* | 0.043 | -4.461 |
| *CSF1R* | 0.043 | -4.652 |
| *YPEL5* | 0.043 | -3.124 |
| *ZBTB26* | 0.043 | -4.509 |
| *ASNS* | 0.044 | 4.668 |
| *PDE5A* | 0.044 | -4.546 |
| *RNVU1-7* | 0.044 | 5.410 |
| *S1PR3* | 0.044 | -4.874 |
| *FAM71F2* | 0.044 | 4.839 |
| *KIAA0319* | 0.044 | 5.468 |
| *EEF1E1* | 0.044 | 3.848 |
| *PCNP* | 0.044 | -3.357 |
| *PDZD11* | 0.044 | -4.201 |
| *ZNF639* | 0.044 | -4.543 |
| *FBLIM1* | 0.045 | -4.511 |
| *TXNDC15* | 0.045 | -4.554 |
| *ZNF876P* | 0.045 | 4.421 |
| *MAPK14* | 0.045 | -3.050 |
| *QTRT1* | 0.046 | -4.499 |
| *PKDREJ* | 0.046 | 5.320 |
| *TRIM44* | 0.046 | -2.630 |
| *PPA2* | 0.046 | -2.867 |
| *DNAJC14* | 0.046 | -4.575 |
| *DNAJC22* | 0.047 | -4.868 |
| *PCSK1* | 0.047 | 5.155 |
| *KANK1* | 0.047 | -4.188 |

| | | |
|---|---|---|
| *ZFR* | 0.047 | -2.608 |
| *PSMG3* | 0.048 | -4.489 |
| *PTPRC* | 0.048 | -4.515 |
| *ARV1* | 0.048 | -3.930 |
| *DYNLT3* | 0.048 | -3.983 |
| *TBX3* | 0.048 | -5.302 |
| *ZNF438* | 0.048 | -4.617 |
| *GSTCD-AS1* | 0.049 | 5.983 |
| *CCDC87* | 0.049 | 5.983 |
| *C12orf71* | 0.049 | 5.983 |
| *ZNF136* | 0.049 | -4.276 |
| *TBP* | 0.049 | -4.831 |
| *POLR1E* | 0.049 | 3.419 |

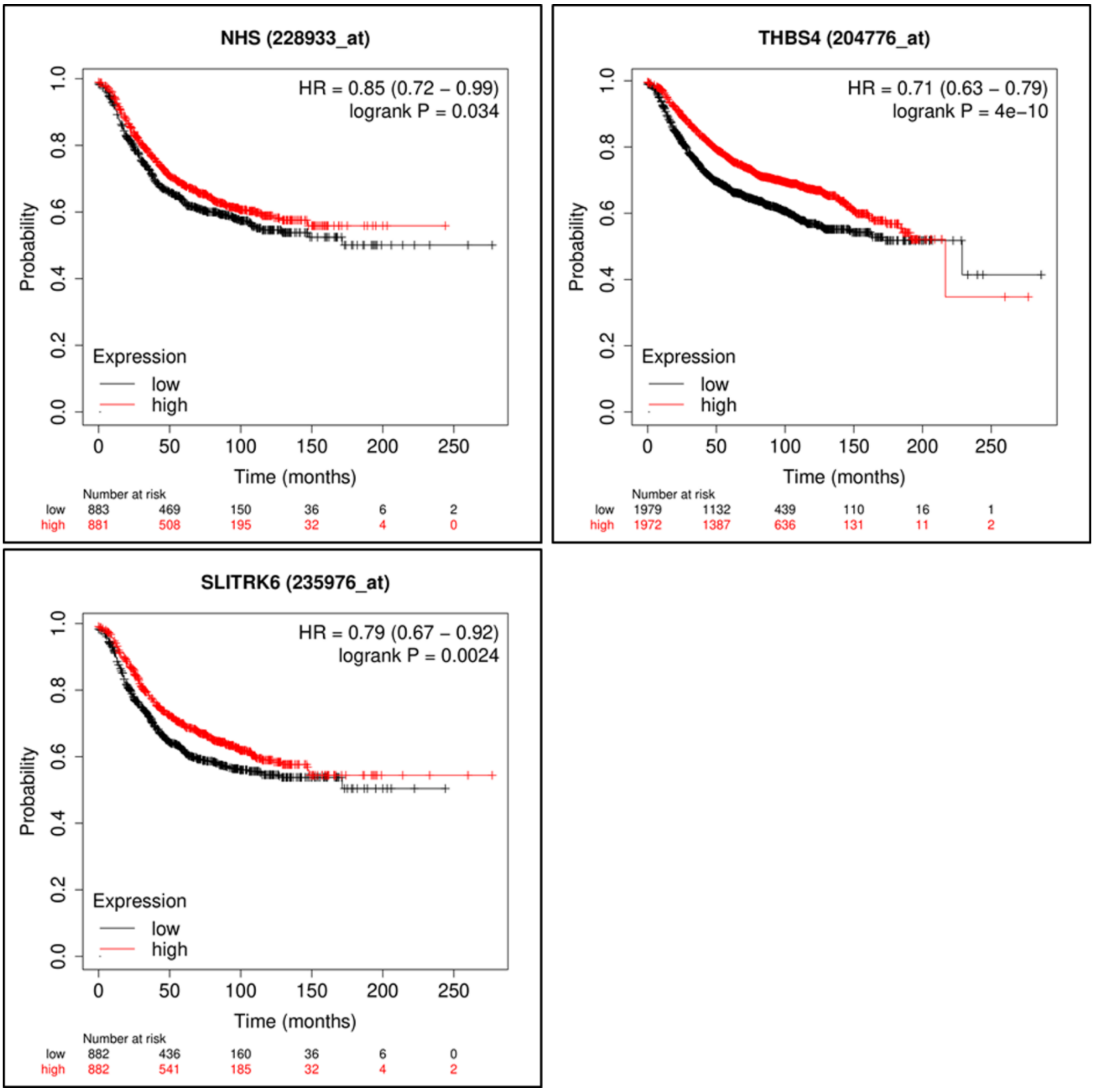


**Figure S5.** Association of DEGs (*NHC*, *THBS4* and *SLITRK6*) in KIF14-positive cells with relapse-free survival of breast cancer patients according to Kaplan-Meyer Plotter database.

**Table S8.** KEGG enrichment analysis of up- and down-regulated genes in KIF14-positive cells located in torpedo-like structures ($p < 0.05$)

| Term | P-value | Adjusted P-value | Genes |
|---|---|---|---|
| VEGF signaling pathway | 0.001 | 0.311 | *PLA2G4F; NRAS; PLA2G4C; PIK3CB; MAPK14* |
| Fc epsilon RI signaling pathway | 0.002 | 0.294 | *PLA2G4F; NRAS; PLA2G4C; PIK3CB; MAPK14* |
| Ether lipid metabolism | 0.003 | 0.330 | *UGT8; PLA2G4F; PLA2G4C; PLA2G7* |
| GnRH signaling pathway | 0.007 | 0.565 | *PLA2G4F; NRAS; PLCB4; PLA2G4C; MAPK14* |
| Long-term depression | 0.008 | 0.474 | *PLA2G4F; NRAS; PLCB4; PLA2G4C* |
| Arachidonic acid metabolism | 0.009 | 0.468 | *PLA2G4F; CYP2U1; PLA2G4C; CYP4F8* |
| AGE-RAGE signaling pathway in diabetic complications | 0.010 | 0.435 | *NRAS; PLCB4; NFATC1; PIK3CB; MAPK14* |
| Inflammatory mediator regulation of TRP channels | 0.010 | 0.381 | *PLA2G4F; PLCB4; PLA2G4C; PIK3CB; MAPK14* |
| T cell receptor signaling pathway | 0.010 | 0.352 | *NRAS; PTPRC; NFATC1; PIK3CB; MAPK14* |
| Acute myeloid leukemia | 0.011 | 0.330 | *CSF1R; NRAS; BCL2A1; PIK3CB* |
| Phospholipase D signaling pathway | 0.013 | 0.363 | *PLA2G4F; NRAS; PLCB4; RALB; PLA2G4C; PIK3CB* |
| Prolactin signaling pathway | 0.013 | 0.336 | *NRAS; PIK3CB; SOCS6; MAPK14* |
| Oxytocin signaling pathway | 0.015 | 0.357 | *PLA2G4F; NRAS; CAMK1D; PLCB4; PLA2G4C; NFATC1* |
| Inositol phosphate metabolism | 0.016 | 0.347 | *PLCB4; IPMK; INPP5J; PIK3CB* |
| Rap1 signaling pathway | 0.018 | 0.379 | *CSF1R; NRAS; PLCB4; RALB; PIK3CB; MAPK14; FGFR1* |
| Sphingolipid signaling pathway | 0.020 | 0.379 | *NRAS; PLCB4; S1PR3; PIK3CB; MAPK14* |
| Platelet activation | 0.023 | 0.418 | *PLA2G4F; PLCB4; PLA2G4C; PIK3CB; MAPK14* |
| Osteoclast differentiation | 0.025 | 0.432 | *CSF1R; SIRPA; NFATC1; PIK3CB; MAPK14* |
| Relaxin signaling pathway | 0.028 | 0.447 | *NRAS; MMP13; PLCB4; PIK3CB; MAPK14* |
| Vascular smooth muscle contraction | 0.029 | 0.450 | *PLA2G4F; EDN2; PLCB4; CALD1; PLA2G4C* |
| Fc gamma R-mediated phagocytosis | 0.031 | 0.454 | *PLA2G4F; PTPRC; ARPC5L; PIK3CB* |
| Ras signaling pathway | 0.032 | 0.454 | *CSF1R; NRAS; PLA2G4F; RALB; PLA2G4C; PIK3CB; FGFR1* |
| Signaling pathways regulating pluripotency of stem cells | 0.035 | 0.473 | *NRAS; PIK3CB; MAPK14; TBX3; FGFR1* |

| | | | |
|---|---|---|---|
| Choline metabolism in cancer | 0.040 | 0.517 | *PLA2G4F; NRAS; PLA2G4C; PIK3CB* |
| Phosphatidylinositol signaling system | 0.040 | 0.496 | *PLCB4; IPMK; INPP5J; PIK3CB* |
| alpha-Linolenic acid metabolism | 0.042 | 0.492 | *PLA2G4F; PLA2G4C* |
| C-type lectin receptor signaling pathway | 0.047 | 0.535 | *NRAS; NFATC1; PIK3CB; MAPK14* |

A total of 105 upregulated and 155 downregulated genes have been analyzed

**Table S9.** GO enrichment analysis of up- and down-regulated genes in KIF14-positive cells located in torpedo-like structures ($p < 0.05$)

| Term | P-value | Adjusted P-value | Genes |
|---|---|---|---|
| phosphate-containing compound metabolic process (GO:0006796) | 0.001 | 1.000 | *PLA2G4F; PTPRC; PLCB4; IPMK; PLA2G4C; INPP5J; ACYP2; PIK3CB; LIMD1* |
| negative regulation of signal transduction (GO:0009968) | 0.001 | 1.000 | *EPHB6; KANK1; CSF1R; LEPROT; PTPRC; NEURL1; CRY1; KLK14; NFATC1; EPHB1; FGFR1* |
| negative regulation of DNA metabolic process (GO:0051053) | 0.002 | 1.000 | *ZRANB3; DFFA; CDAN1; ERCC4* |
| phosphatidylinositol metabolic process (GO:0046488) | 0.003 | 1.000 | *CSF1R; PLA2G4F; PLA2G4C; C2ORF66; INPP5J; PIK3CB* |
| positive regulation of kinase activity (GO:0033674) | 0.004 | 1.000 | *EPHB6; CSF1R; PTPRC; EEF1A2; EPHB1; FGFR1* |
| regulation of cellular protein localization (GO:1903827) | 0.004 | 1.000 | *RALB; LCP1; RAB11FIP5* |
| positive regulation of voltage-gated potassium channel activity (GO:1903818) | 0.006 | 1.000 | *LRRC26; NOS1AP* |
| negative regulation of heterotypic cell-cell adhesion (GO:0034115) | 0.006 | 1.000 | *IL1RN; MYADM* |
| regulation of cell-cell adhesion involved in gastrulation (GO:0070587) | 0.006 | 1.000 | *IL1RN; MYADM* |
| collagen fibril organization (GO:0030199) | 0.006 | 1.000 | *COL2A1; LUM; DPT* |
| keratan sulfate biosynthetic process (GO:0018146) | 0.006 | 1.000 | *B4GAT1; LUM; B4GALT6* |
| keratan sulfate metabolic process (GO:0042339) | 0.010 | 1.000 | *B4GAT1; LUM; B4GALT6* |
| PERK-mediated unfolded protein response (GO:0036499) | 0.010 | 1.000 | *ASNS; ATF3* |
| extracellular matrix organization (GO:0030198) | 0.010 | 1.000 | *CSGALNACT1; SCUBE3; COL2A1; MMP13; LUM; DPT; TLL2; LCP1* |
| regulation of execution phase of apoptosis (GO:1900117) | 0.012 | 1.000 | *DFFA; DLC1* |

| | | | |
|---|---|---|---|
| negative regulation of Rho protein signal transduction (GO:0035024) | 0.014 | 1.000 | *KANK1; DLC1* |
| inositol lipid-mediated signaling (GO:0048017) | 0.014 | 1.000 | *CSF1R; PIK3CB; FGFR1* |
| tRNA aminoacylation (GO:0043039) | 0.015 | 1.000 | *PPA2; PPA1; EEF1E1* |
| regulation of clathrin-dependent endocytosis (GO:2000369) | 0.016 | 1.000 | *BMP2K; PIK3CB* |
| protein O-linked glycosylation (GO:0006493) | 0.017 | 1.000 | *B4GAT1; GALNT3; B3GALT2; GCNT3; ADAMTS7* |
| phosphatidylinositol acyl-chain remodeling (GO:0036149) | 0.018 | 1.000 | *PLA2G4F; PLA2G4C* |
| regulation of reactive oxygen species biosynthetic process (GO:1903426) | 0.018 | 1.000 | *GPRIN1; NOS1AP* |
| response to ionizing radiation (GO:0010212) | 0.018 | 1.000 | *RAD51B; EYA1; ERCC4; MAPK14* |
| negative regulation of cell communication (GO:0010648) | 0.019 | 1.000 | *EPHB6; CSF1R; EPHB1; FGFR1* |
| extracellular matrix disassembly (GO:0022617) | 0.019 | 1.000 | *SCUBE3; MMP13; TLL2; LCP1* |
| regulation of telomere maintenance via telomere lengthening (GO:1904356) | 0.020 | 1.000 | *ERCC4; POT1* |
| DNA packaging (GO:0006323) | 0.020 | 1.000 | *CDAN1; NAA10* |
| detection of mechanical stimulus (GO:0050982) | 0.020 | 1.000 | *DENND5B; PKDREJ* |
| protein complex subunit organization (GO:0071822) | 0.021 | 1.000 | *COL2A1; LUM; DPT* |
| sulfur compound biosynthetic process (GO:0044272) | 0.022 | 1.000 | *CSGALNACT1; SLC26A2; B4GAT1; LUM; B4GALT6* |
| tRNA aminoacylation for protein translation (GO:0006418) | 0.022 | 1.000 | *PPA2; PPA1; EEF1E1* |
| DNA conformation change (GO:0071103) | 0.022 | 1.000 | *TOP3B; NAA10* |
| negative regulation of signaling (GO:0023057) | 0.023 | 1.000 | *EPHB6; CSF1R; EPHB1; FGFR1* |
| polyol metabolic process (GO:0019751) | 0.023 | 1.000 | *PLCB4; IPMK; INPP5J* |
| inositol phosphate metabolic process (GO:0043647) | 0.023 | 1.000 | *PLCB4; IPMK; INPP5J* |

| | | | |
|---|---|---|---|
| neutrophil degranulation (GO:0043312) | 0.023 | 1.000 | *ADGRG3; NRAS; PTPRC; DGAT1; ANXA3; DLC1; NAPRT; RAB18; SIRPA; YPEL5; MAPK14; CAMP* |
| neutrophil activation involved in immune response (GO:0002283) | 0.025 | 1.000 | *ADGRG3; NRAS; PTPRC; DGAT1; ANXA3; DLC1; NAPRT; RAB18; SIRPA; YPEL5; MAPK14; CAMP* |
| regulation of heterotypic cell-cell adhesion (GO:0034114) | 0.025 | 1.000 | *IL1RN; MYADM* |
| osteoclast differentiation (GO:0030316) | 0.025 | 1.000 | *CSF1R; MAPK14* |
| neutrophil mediated immunity (GO:0002446) | 0.026 | 1.000 | *ADGRG3; NRAS; PTPRC; DGAT1; ANXA3; DLC1; NAPRT; RAB18; SIRPA; YPEL5; MAPK14; CAMP* |
| proteoglycan metabolic process (GO:0006029) | 0.027 | 1.000 | *CSGALNACT1; GAL3ST4* |
| inositol phosphate-mediated signaling (GO:0048016) | 0.027 | 1.000 | *EDN2; NFATC1* |
| positive regulation of lipid metabolic process (GO:0045834) | 0.030 | 1.000 | *EEF1A2; CREBL2* |
| negative regulation of G-protein coupled receptor protein signaling pathway (GO:0045744) | 0.030 | 1.000 | *CRY1; KLK14* |
| negative regulation of protein polymerization (GO:0032272) | 0.033 | 1.000 | *KANK1; MYADM* |
| positive regulation of biosynthetic process (GO:0009891) | 0.033 | 1.000 | *GPRIN1; CREBL2* |
| negative regulation of actin filament polymerization (GO:0030837) | 0.033 | 1.000 | *KANK1; MYADM* |
| positive regulation of transferase activity (GO:0051347) | 0.033 | 1.000 | *EPHB6; CSF1R; POT1; EPHB1; FGFR1* |
| phosphatidylinositol-mediated signaling (GO:0048015) | 0.036 | 1.000 | *CSF1R; PIK3CB; FGFR1* |
| positive regulation of neutrophil chemotaxis (GO:0090023) | 0.039 | 1.000 | *CAMK1D; THBS4* |
| negative regulation of cytoskeleton organization (GO:0051494) | 0.039 | 1.000 | *KANK1; MYADM* |
| DNA geometric change (GO:0032392) | 0.039 | 1.000 | *ZRANB3; POT1* |

| | | | |
|---|---|---|---|
| proteoglycan biosynthetic process (GO:0030166) | 0.039 | 1.000 | *CSGALNACT1; GAL3ST4* |
| cellular senescence (GO:0090398) | 0.039 | 1.000 | *MAPK14; TBX3* |
| glycosaminoglycan biosynthetic process (GO:0006024) | 0.040 | 1.000 | *CSGALNACT1; B4GAT1; LUM; B4GALT6* |
| positive regulation of neutrophil migration (GO:1902624) | 0.042 | 1.000 | *CAMK1D; THBS4* |
| prostaglandin metabolic process (GO:0006693) | 0.042 | 1.000 | *EDN2; CYP4F8* |
| regulation of kinase activity (GO:0043549) | 0.043 | 1.000 | *EPHB6; CSF1R; EPHB1; FGFR1* |
| phosphatidylethanolamine acyl-chain remodeling (GO:0036152) | 0.045 | 1.000 | *PLA2G4F; PLA2G4C* |
| positive regulation of granulocyte chemotaxis (GO:0071624) | 0.045 | 1.000 | *CAMK1D; THBS4* |
| negative regulation of telomere maintenance via telomere lengthening (GO:1904357) | 0.045 | 1.000 | *ERCC4; POT1* |
| peptide metabolic process (GO:0006518) | 0.047 | 1.000 | *PCSK1; PROS1; EEF1E1; CPN1* |
| phosphatidylcholine acyl-chain remodeling (GO:0036151) | 0.048 | 1.000 | *PLA2G4F; PLA2G4C* |
| peptidyl-glutamic acid modification (GO:0018200) | 0.048 | 1.000 | *NAA10; PROS1* |
| transcription from RNA polymerase III promoter (GO:0006383) | 0.048 | 1.000 | *TBP; SNAPC1* |
| regulation of protein localization to cell surface (GO:2000008) | 0.048 | 1.000 | *LEPROT; RAB11FIP5* |
| oligosaccharide metabolic process (GO:0009311) | 0.049 | 1.000 | *GALNT3; B3GALT2; GAL3ST4* |

A total of 105 upregulated and 155 downregulated genes have been analyzed

**Table S10.** Differentially expressed genes between Mieap-positive and Mieap-negative cells located in torpedo-like structures ($p < 0.05$)

| Genes | P-value | LogFC |
|---|---|---|
| *FOSB* | 0.000 | -8.642 |
| *CCDC18* | 0.001 | 4.732 |
| *GPR153* | 0.001 | -7.251 |
| *ZNF337* | 0.001 | -7.229 |
| *MYH4* | 0.001 | -8.198 |
| *IER5* | 0.001 | -4.042 |
| *MTG1* | 0.001 | -6.903 |
| *SAMD9* | 0.001 | 7.136 |
| *OLFML2B* | 0.001 | -7.023 |
| *KDR* | 0.002 | -7.390 |
| *COL4A1* | 0.002 | -4.392 |
| *MYH3* | 0.002 | -5.666 |
| *SLIT2* | 0.002 | -6.863 |
| *RRAS2* | 0.002 | 6.386 |
| *FOPNL* | 0.002 | 5.779 |
| *DGKI* | 0.002 | -7.395 |
| *MED10* | 0.002 | -6.759 |
| *TACC1* | 0.002 | -3.804 |
| *KLHL7* | 0.002 | 5.690 |
| *KDM4C* | 0.003 | 4.068 |
| *LIAS* | 0.003 | 6.305 |
| *B4GALT6* | 0.003 | -7.148 |
| *MFSD8* | 0.003 | 6.289 |
| *NOTCH4* | 0.003 | -7.197 |
| *BIN2* | 0.003 | -7.121 |
| *TCF4* | 0.003 | -6.239 |
| *PRPF18* | 0.004 | 6.746 |
| *ACTG2* | 0.004 | -7.096 |
| *RASD1* | 0.004 | -5.307 |
| *DHFR2* | 0.004 | 5.975 |
| *TOE1* | 0.004 | 6.024 |
| *FAM110B* | 0.005 | -6.496 |
| *RMI2* | 0.005 | 6.043 |
| *LTB4R2* | 0.005 | -6.939 |
| *RSAD1* | 0.005 | 6.041 |
| *MXRA7* | 0.005 | -6.586 |
| *STK32B* | 0.005 | -6.910 |
| *LACTB2* | 0.005 | 6.003 |
| *CAPN9* | 0.006 | -6.896 |
| *NPR2* | 0.006 | -6.273 |
| *MBIP* | 0.006 | 6.541 |
| *C17orf58* | 0.006 | 6.491 |
| *CDO1* | 0.006 | -6.833 |
| *SLC2A13* | 0.007 | 6.359 |
| *BICDL1* | 0.007 | 5.241 |
| *MMP11* | 0.007 | -6.834 |
| *KIF26B* | 0.007 | -4.854 |

| | | |
|---|---|---|
| *ADAMTS6* | 0.007 | -6.756 |
| *CENPL* | 0.008 | 5.946 |
| *THY1* | 0.008 | -6.283 |
| *APEX2* | 0.008 | 5.242 |
| *PLEK2* | 0.008 | -6.383 |
| *SYNPO2* | 0.008 | -6.323 |
| *DUSP22* | 0.009 | -5.930 |
| *SLC26A8* | 0.009 | -6.691 |
| *TEKT2* | 0.010 | -6.663 |
| *H3C7* | 0.010 | 5.700 |
| *ACTA1* | 0.010 | -5.537 |
| *SUSD4* | 0.010 | -6.651 |
| *PDCD1LG2* | 0.010 | -6.053 |
| *MRVI1* | 0.011 | -6.120 |
| *ISOC1* | 0.011 | 3.816 |
| *ZNF17* | 0.011 | 5.715 |
| *RNF181* | 0.011 | 5.035 |
| *ZNF423* | 0.012 | -5.243 |
| *FSCN2* | 0.012 | -6.534 |
| *KXD1* | 0.013 | 4.521 |
| *SYT11* | 0.013 | -6.548 |
| *ZNF470* | 0.013 | -5.880 |
| *BCYRN1* | 0.014 | 4.066 |
| *BOC* | 0.014 | -5.814 |
| *VAV1* | 0.014 | -6.492 |
| *H2BC11* | 0.014 | 5.046 |
| *SCAMP5* | 0.015 | 6.045 |
| *PHF11* | 0.016 | 3.318 |
| *SLC10A5* | 0.016 | -6.449 |
| *M1AP* | 0.016 | -6.523 |
| *C19orf18* | 0.016 | 5.950 |
| *H1-6* | 0.017 | -5.919 |
| *MED7* | 0.017 | 5.438 |
| *PKNOX1* | 0.017 | -4.008 |
| *SDAD1P1* | 0.017 | 5.940 |
| *GLI2* | 0.017 | -6.544 |
| *WRAP73* | 0.018 | 5.444 |
| *ZNF624* | 0.018 | -3.533 |
| *ATP8A2* | 0.018 | -6.378 |
| *ZEB2* | 0.018 | -3.619 |
| *PALB2* | 0.019 | 2.613 |
| *PGAM5* | 0.019 | 5.881 |
| *MRPL23* | 0.019 | 5.500 |
| *LOC441666* | 0.020 | -5.324 |
| *CSGALNACT1* | 0.020 | 3.520 |
| *RPH3A* | 0.021 | -6.315 |
| *PCGF1* | 0.021 | 5.384 |
| *DOK4* | 0.021 | -6.280 |
| *P3H3* | 0.022 | -6.288 |
| *CSTF1* | 0.022 | 2.818 |

| | | |
|---|---|---|
| *C9orf43* | 0.022 | -4.995 |
| *LINC00536* | 0.022 | 5.554 |
| *CTSC* | 0.023 | -4.773 |
| *DBP* | 0.023 | 5.847 |
| *USP49* | 0.023 | 4.744 |
| *SPARCL1* | 0.023 | -3.782 |
| *C3* | 0.023 | -3.234 |
| *EIF6* | 0.023 | 4.055 |
| *ACTR10* | 0.024 | 2.868 |
| *CCDC192* | 0.024 | -6.232 |
| *SPAG6* | 0.024 | -5.620 |
| *NCS1* | 0.024 | -4.655 |
| *ZSWIM5* | 0.025 | 5.783 |
| *SCN3A* | 0.025 | -6.288 |
| *HTRA1* | 0.025 | -4.905 |
| *MRPL22* | 0.026 | -6.204 |
| *A2M* | 0.026 | -3.680 |
| *IL15RA* | 0.026 | -6.258 |
| *IFIT1* | 0.026 | 4.646 |
| *PIP4K2A* | 0.026 | -3.162 |
| *LSM5* | 0.026 | 2.546 |
| *ZNF256* | 0.026 | 5.286 |
| *SUFU* | 0.027 | -2.885 |
| *CRLF3* | 0.027 | -3.588 |
| *SLC7A6* | 0.027 | 5.255 |
| *FBXL6* | 0.027 | 4.603 |
| *ZNF837* | 0.027 | 5.248 |
| *TMEM75* | 0.027 | 4.870 |
| *DPM1* | 0.028 | 3.254 |
| *PRKAB1* | 0.028 | 4.163 |
| *EIF4A1* | 0.028 | -4.955 |
| *RTCB* | 0.028 | 3.101 |
| *STPG3-AS1* | 0.029 | -6.143 |
| *SGO1-AS1* | 0.029 | 5.304 |
| *NEURL1B* | 0.029 | 5.706 |
| *CPXM2* | 0.029 | -6.129 |
| *ADCY10* | 0.029 | -5.768 |
| *DNAJC4* | 0.029 | 4.135 |
| *WNT4* | 0.029 | 5.319 |
| *MPZL3* | 0.030 | 3.347 |
| *ZNF573* | 0.030 | 3.455 |
| *C11orf1* | 0.030 | 5.331 |
| *MAGI1-AS1* | 0.031 | 5.248 |
| *FNDC1* | 0.031 | -3.688 |
| *MRPS15* | 0.031 | 2.897 |
| *TRAF3IP3* | 0.031 | -4.871 |
| *POMK* | 0.032 | -3.259 |
| *AXDND1* | 0.032 | 5.638 |
| *MAP2K6* | 0.032 | -2.863 |
| *BMPR1A* | 0.032 | 3.064 |

| | | |
|---|---|---|
| *RNF225* | 0.033 | 5.685 |
| *GNRHR* | 0.033 | 5.618 |
| *TCEANC* | 0.033 | 5.218 |
| *CPVL* | 0.033 | -5.128 |
| *TRIM59* | 0.033 | 5.201 |
| *HSPA13* | 0.033 | 5.184 |
| *SLC39A14* | 0.033 | -3.540 |
| *MMD* | 0.033 | -6.072 |
| *FH* | 0.034 | 3.467 |
| *MOCS3* | 0.034 | 3.806 |
| *STK16* | 0.035 | -4.559 |
| *CD248* | 0.035 | -6.029 |
| *CDK8* | 0.035 | 3.180 |
| *PDE12* | 0.035 | -2.416 |
| *KBTBD8* | 0.035 | -6.059 |
| *ZMYM4-AS1* | 0.035 | -6.022 |
| *URM1* | 0.036 | 4.547 |
| *RBP5* | 0.037 | -5.315 |
| *MPZL2* | 0.037 | -3.372 |
| *JMJD4* | 0.037 | -4.479 |
| *CTDNEP1* | 0.038 | 2.824 |
| *CX3CL1* | 0.038 | -3.549 |
| *NMD3* | 0.038 | -2.116 |
| *DIPK1B* | 0.038 | -5.972 |
| *FLJ37453* | 0.038 | -5.985 |
| *CPAMD8* | 0.038 | -2.362 |
| *CDKN2AIPNL* | 0.038 | 5.207 |
| *EDDM13* | 0.038 | 4.080 |
| *ZNF763* | 0.038 | 5.095 |
| *IER2* | 0.039 | -3.345 |
| *C8orf58* | 0.039 | 4.436 |
| *KRT81* | 0.039 | -5.969 |
| *TAP1* | 0.039 | 5.467 |
| *SPI1* | 0.040 | -5.940 |
| *TMEM44* | 0.040 | -5.253 |
| *MALRD1* | 0.041 | 5.496 |
| *ILK* | 0.041 | -5.576 |
| *PKD2* | 0.041 | -4.585 |
| *DENND4B* | 0.041 | -2.554 |
| *HABP4* | 0.041 | -6.034 |
| *LOC102724788* | 0.042 | 5.494 |
| *USP18* | 0.042 | 4.959 |
| *NOC4L* | 0.042 | -3.707 |
| *ZNF284* | 0.042 | -5.239 |
| *COX10* | 0.043 | 5.417 |
| *ZNF200* | 0.043 | 3.615 |
| *EXOSC2* | 0.043 | 3.617 |
| *MX2* | 0.044 | 3.849 |
| *ETS1* | 0.044 | -4.910 |
| *SLC29A4* | 0.044 | 5.070 |

| | | |
|---|---|---|
| *RPL39* | 0.044 | -3.729 |
| *RBAK* | 0.044 | 2.460 |
| *PRC1-AS1* | 0.045 | 5.022 |
| *TMEM70* | 0.045 | -4.425 |
| *HEIH* | 0.045 | -5.893 |
| *CARD9* | 0.045 | 3.534 |
| *LINC01811* | 0.045 | -5.877 |
| *HMCN1* | 0.046 | -4.058 |
| *MAP3K21* | 0.046 | 5.433 |
| *ZC2HC1C* | 0.046 | 5.017 |
| *ST7* | 0.047 | -3.950 |
| *COA3* | 0.047 | 5.019 |
| *NINJ2-AS1* | 0.047 | 5.057 |
| *ADAMTS16* | 0.048 | -5.848 |
| *CAVIN3* | 0.048 | -5.855 |
| *PROSER1* | 0.048 | 2.218 |
| *TMEM69* | 0.048 | 4.343 |
| *EOGT* | 0.049 | -4.266 |
| *CCDC122* | 0.049 | 3.098 |
| *COL4A2* | 0.049 | -3.012 |
| *BNIP3* | 0.049 | 2.278 |
| *KIAA1671-AS1* | 0.049 | -4.611 |
| *ZNF517* | 0.049 | 5.382 |
| *BCL7B* | 0.049 | 2.836 |
| *TFPI2* | 0.049 | 3.871 |
| *H2AC16* | 0.049 | 5.319 |
| *BTN2A3P* | 0.049 | 5.401 |
| *TIGD7* | 0.049 | 5.320 |
| *SYT14* | 0.049 | -5.841 |

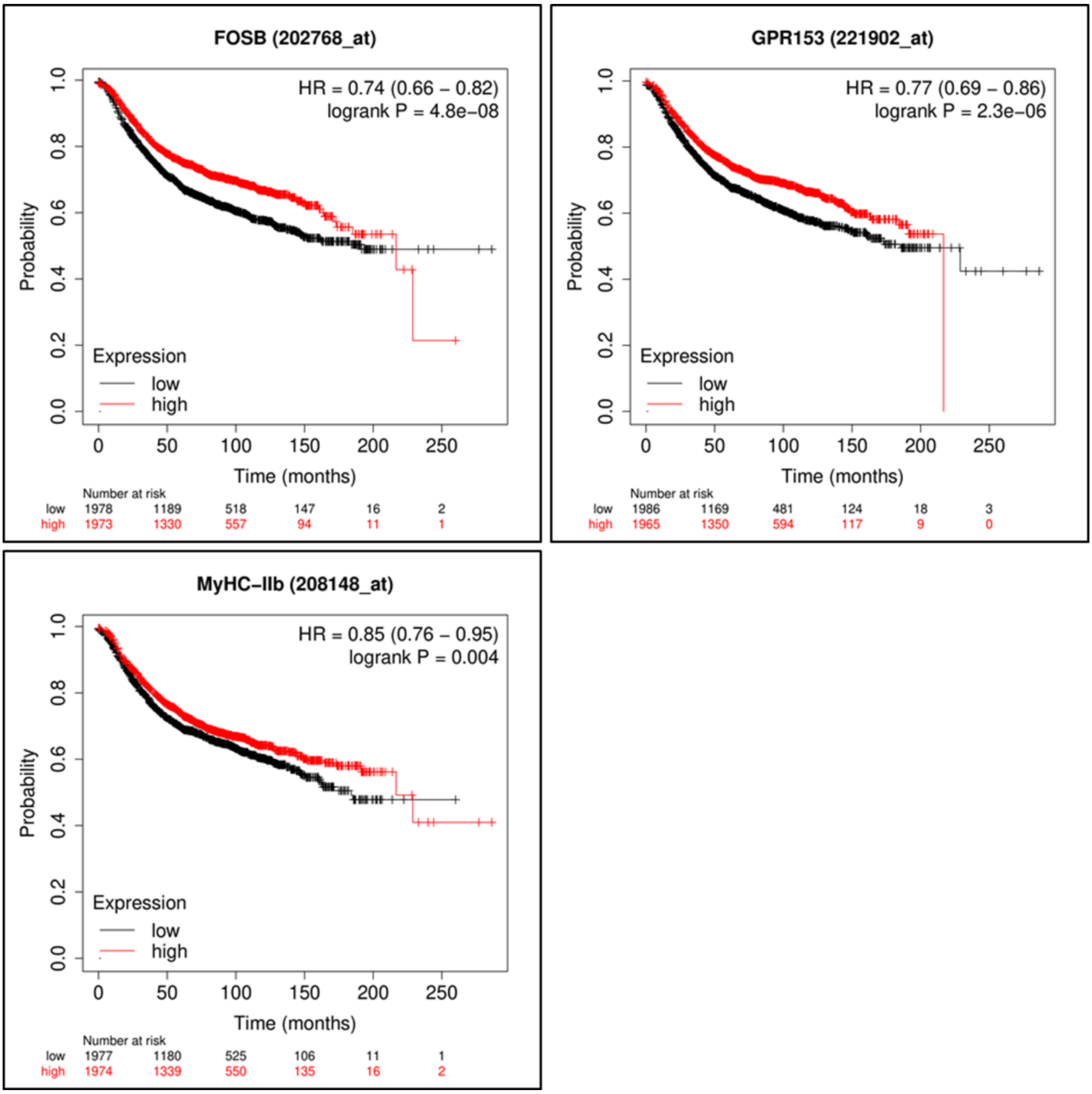


**Figure S6.** Association of DEGs (*FOSB*, *GPR153* and *MYH4*) in Mieap-positive cells with relapse-free survival of breast cancer patients according to Kaplan-Meyer Plotter database.

**Table S11.** KEGG enrichment analysis of up- and down-regulated genes in Mieap-positive cells located in torpedo-like structures ($p < 0.05$)

| Term | P-value | Adjusted P-value | Genes |
|---|---|---|---|
| Sulfur relay system | 0.003 | 1.000 | *MOCS3; URM1* |
| Herpes simplex virus 1 infection | 0.004 | 0.629 | *ZNF17; ZNF284; ZNF470; CARD9; TAP1; C3; ZNF517; ZNF624; ZNF337; RBAK; ZNF763; ZNF256; ZNF200* |
| Hedgehog signaling pathway | 0.016 | 1.000 | *SUFU; BOC; GLI2* |
| Basal cell carcinoma | 0.034 | 1.000 | *SUFU; GLI2; WNT4* |
| Mitophagy | 0.037 | 1.000 | *BNIP3; RRAS2; PGAM5* |

A total of 106 upregulated and 120 downregulated genes have been analyzed

**Table S12.** GO enrichment analysis of up- and down-regulated genes in Mieap-positive cells located in torpedo-like structures ($p < 0.05$)

| Term | P-value | Adjusted P-value | Genes |
|---|---|---|---|
| branching morphogenesis of an epithelial tube (GO:0048754) | 0.000 | 0.610 | *COL4A1; NOTCH4; SLIT2; PKD2; GLI2* |
| epithelial tube morphogenesis (GO:0060562) | 0.001 | 1.000 | *COL4A1; SLIT2; PKD2; GLI2* |
| heterocycle biosynthetic process (GO:0018130) | 0.001 | 1.000 | *MOCS3; MBIP; LIAS* |
| mammary gland development (GO:0030879) | 0.002 | 1.000 | *NOTCH4; GLI2; WNT4* |
| pri-miRNA transcription from RNA polymerase II promoter (GO:0061614) | 0.003 | 1.000 | *SPI1; ETS1* |
| molybdopterin cofactor biosynthetic process (GO:0032324) | 0.003 | 1.000 | *MOCS3; MBIP* |
| molybdopterin cofactor metabolic process (GO:0043545) | 0.003 | 1.000 | *MOCS3; MBIP* |
| regulation of calcium ion-dependent exocytosis (GO:0017158) | 0.003 | 1.000 | *SCAMP5; RPH3A; SYT11; SYT14* |
| RNA phosphodiester bond hydrolysis (GO:0090501) | 0.004 | 1.000 | *TOE1; LACTB2; PDE12* |
| vesicle fusion (GO:0006906) | 0.004 | 1.000 | *RPH3A; SAMD9; SYT11; TAP1; SYT14* |
| tRNA wobble uridine modification (GO:0002098) | 0.004 | 1.000 | *MOCS3; URM1* |
| tRNA wobble base modification (GO:0002097) | 0.005 | 1.000 | *MOCS3; URM1* |
| central nervous system projection neuron axonogenesis (GO:0021952) | 0.005 | 1.000 | *SLIT2; GLI2* |
| nuclear-transcribed mRNA catabolic process, deadenylation-dependent decay (GO:0000288) | 0.006 | 1.000 | *EIF4A1; PDE12; LSM5; EXOSC2* |
| metanephric mesenchyme development (GO:0072075) | 0.007 | 1.000 | *PKD2; WNT4* |
| spinal cord development (GO:0021510) | 0.007 | 1.000 | *PKD2; GLI2* |
| ribosomal subunit export from nucleus (GO:0000054) | 0.008 | 1.000 | *EIF6; NMD3* |
| regulation of regulated secretory pathway (GO:1903305) | 0.008 | 1.000 | *RPH3A; SYT11; SYT14* |
| organic cyclic compound biosynthetic process (GO:1901362) | 0.008 | 1.000 | *MOCS3; MBIP; LIAS* |

| | | | |
|---|---|---|---|
| actin-myosin filament sliding (GO:0033275) | 0.009 | 1.000 | *ACTA1; MYH3; MYH4* |
| muscle filament sliding (GO:0030049) | 0.009 | 1.000 | *ACTA1; MYH3; MYH4* |
| regulation of triglyceride biosynthetic process (GO:0010866) | 0.009 | 1.000 | *C3; CTDNEP1* |
| positive regulation of cellular biosynthetic process (GO:0031328) | 0.010 | 1.000 | *CTDNEP1; ZEB2; EIF6; NMD3; PKD2; GLI2* |
| gland development (GO:0048732) | 0.010 | 1.000 | *NOTCH4; PKD2; GLI2; WNT4* |
| branching involved in blood vessel morphogenesis (GO:0001569) | 0.011 | 1.000 | *COL4A1; NOTCH4* |
| negative regulation of smooth muscle cell migration (GO:0014912) | 0.011 | 1.000 | *SLIT2; BMPR1A* |
| positive regulation of mitochondrial fission (GO:0090141) | 0.011 | 1.000 | *BNIP3; KDR* |
| cell migration involved in sprouting angiogenesis (GO:0002042) | 0.011 | 1.000 | *KDR; SLIT2* |
| regulation of mitochondrial translation (GO:0070129) | 0.012 | 1.000 | *COA3; MTG1* |
| cytoplasmic sequestering of protein (GO:0051220) | 0.012 | 1.000 | *SUFU; PKD2* |
| positive regulation of macroautophagy (GO:0016239) | 0.013 | 1.000 | *BNIP3; KDR; PIP4K2A* |
| cellular response to dsRNA (GO:0071359) | 0.014 | 1.000 | *PDE12; IFIT1* |
| cytoplasmic sequestering of transcription factor (GO:0042994) | 0.014 | 1.000 | *SUFU; PKD2* |
| positive regulation of focal adhesion assembly (GO:0051894) | 0.015 | 1.000 | *KDR; THY1* |
| response to interferon-alpha (GO:0035455) | 0.015 | 1.000 | *PDE12; MX2* |
| ureteric bud development (GO:0001657) | 0.015 | 1.000 | *SLIT2; PKD2* |
| mesonephric tubule development (GO:0072164) | 0.015 | 1.000 | *SLIT2; PKD2* |
| extracellular matrix organization (GO:0030198) | 0.016 | 1.000 | *CSGALNACT1; MMP11; COL4A2; COL4A1; KDR; HTRA1; A2M* |
| blood vessel endothelial cell migration (GO:0043534) | 0.017 | 1.000 | *KDR; SLIT2* |
| regulation of smooth muscle cell migration (GO:0014910) | 0.017 | 1.000 | *SLIT2; BMPR1A* |
| positive regulation of adherens junction organization (GO:1903393) | 0.019 | 1.000 | *KDR; THY1* |

| | | | |
|---|---|---|---|
| regulation of mitochondrial fission (GO:0090140) | 0.019 | 1.000 | *BNIP3; KDR* |
| inactivation of MAPK activity (GO:0000188) | 0.021 | 1.000 | *DUSP22; MBIP* |
| positive regulation of macromolecule biosynthetic process (GO:0010557) | 0.023 | 1.000 | *EIF6; KDR; NMD3; WNT4; GLI2* |
| GTP metabolic process (GO:0046039) | 0.023 | 1.000 | *MOCS3; MBIP* |
| positive regulation of leukocyte cell-cell adhesion (GO:1903039) | 0.023 | 1.000 | *THY1; ETS1* |
| hepaticobiliary system development (GO:0061008) | 0.023 | 1.000 | *PKD2; WNT4* |
| regulation of axonogenesis (GO:0050770) | 0.024 | 1.000 | *THY1; SLIT2; ZSWIM5* |
| cellular response to transforming growth factor beta stimulus (GO:0071560) | 0.027 | 1.000 | *DUSP22; STK16; WNT4; BMPR1A* |
| negative regulation of transcription factor import into nucleus (GO:0042992) | 0.028 | 1.000 | *SUFU; PKD2* |
| positive regulation of cell junction assembly (GO:1901890) | 0.028 | 1.000 | *KDR; THY1* |
| respiratory chain complex IV assembly (GO:0008535) | 0.032 | 1.000 | *COA3; COX10* |
| cellular protein catabolic process (GO:0044257) | 0.033 | 1.000 | *BNIP3; CPVL; CTSC* |
| strand displacement (GO:0000732) | 0.035 | 1.000 | *RMI2; PALB2* |
| endocrine system development (GO:0035270) | 0.035 | 1.000 | *GLI2; WNT4* |
| snRNA 3'-end processing (GO:0034472) | 0.035 | 1.000 | *TOE1; EXOSC2* |
| transcription initiation from RNA polymerase II promoter (GO:0006367) | 0.035 | 1.000 | *CDK8; NOTCH4; MED10; TCF4; MED7* |
| regulation of lipid biosynthetic process (GO:0046890) | 0.040 | 1.000 | *C3; EIF6* |
| coenzyme biosynthetic process (GO:0009108) | 0.042 | 1.000 | *MOCS3; LIAS* |
| regulation of cellular biosynthetic process (GO:0031326) | 0.042 | 1.000 | *C3; EIF6* |
| regulation of endothelial cell migration (GO:0010594) | 0.043 | 1.000 | *KDR; SLIT2; ETS1* |
| regulation of osteoblast differentiation (GO:0045667) | 0.043 | 1.000 | *SUFU; WNT4; BMPR1A* |
| artery morphogenesis (GO:0048844) | 0.045 | 1.000 | *PKD2; BMPR1A* |
| cyclic purine nucleotide metabolic process (GO:0052652) | 0.045 | 1.000 | *ADCY10; NPR2* |

| | | | |
|---|---|---|---|
| organelle membrane fusion (GO:0090174) | 0.046 | 1.000 | *RPH3A; SYT11; SYT14* |
| regulation of gene expression (GO:0010468) | 0.047 | 1.000 | *ZNF17; ZNF573; EIF4A1; ZNF284; ZNF470; PRKAB1; ETS1; ACTG2; ACTA1; ZNF837; SUFU; KDR; PCGF1; ZNF624; RBAK; ZNF256; ZNF200; WNT4* |
| positive regulation of release of sequestered calcium ion into cytosol (GO:0051281) | 0.048 | 1.000 | *THY1; PKD2* |
| liver development (GO:0001889) | 0.048 | 1.000 | *PKD2; WNT4* |
| positive regulation of bone mineralization (GO:0030501) | 0.048 | 1.000 | *WNT4; BMPR1A* |
| positive regulation of viral genome replication (GO:0045070) | 0.048 | 1.000 | *PDE12; IFIT1* |
| cofactor biosynthetic process (GO:0051188) | 0.048 | 1.000 | *MOCS3; MBIP* |
| proteolysis (GO:0006508) | 0.048 | 1.000 | *CAPN9; CPXM2; SUFU; HTRA1; CPVL; CTSC; FBXL6* |
| negative regulation of cell migration (GO:0030336) | 0.049 | 1.000 | *THY1; SLIT2; CX3CL1; BMPR1A* |
| sulfur compound biosynthetic process (GO:0044272) | 0.049 | 1.000 | *CSGALNACT1; LIAS; B4GALT6; CDO1* |

A total of 106 upregulated and 120 downregulated genes have been analyzed

**Table S13.** Differentially expressed genes between EZR-negative and EZR-positive cells located in torpedo-like structures ($p < 0.05$)

| Gene | P-value | LogFC |
|---|---|---|
| *CD109* | 0.000 | 7.836 |
| *ID4* | 0.000 | 7.848 |
| *CA5B* | 0.000 | 7.536 |
| *GCNT7* | 0.000 | 7.273 |
| *ST8SIA1* | 0.000 | 5.093 |
| *NR4A1* | 0.001 | 4.957 |
| *SYCP3* | 0.001 | -5.976 |
| *MAP2* | 0.001 | 6.998 |
| *LINC00539* | 0.001 | 7.450 |
| *SLC5A4* | 0.001 | 7.426 |
| *ZNF416* | 0.001 | 7.004 |
| *CGRRF1* | 0.001 | 5.424 |
| *ADAMTS6* | 0.001 | 7.398 |
| *TMEM72-AS1* | 0.001 | 7.008 |
| *ZBTB42* | 0.001 | 4.585 |
| *DCAF8L1* | 0.001 | 7.244 |
| *FAM174C* | 0.002 | -6.202 |
| *TECTA* | 0.002 | 6.910 |
| *TCHH* | 0.002 | 3.354 |
| *DKK2* | 0.002 | 4.907 |
| *FCGR2B* | 0.002 | 5.947 |
| *SHCBP1* | 0.003 | 5.146 |
| *ERG* | 0.003 | 6.681 |
| *EHHADH* | 0.003 | -6.538 |
| *NR4A3* | 0.003 | 6.266 |
| *SPTB* | 0.004 | 6.095 |
| *SCNN1D* | 0.004 | -5.931 |
| *FBXO44* | 0.004 | 5.561 |
| *JAG1* | 0.004 | 3.777 |
| *ZNF48* | 0.005 | -5.777 |
| *MYH11* | 0.005 | 4.037 |
| *ADAMTS10* | 0.005 | 6.698 |
| *ALMS1P1* | 0.005 | -5.905 |
| *MFSD13A* | 0.006 | -5.718 |
| *SLC26A8* | 0.006 | 5.865 |
| *EME1* | 0.006 | 3.973 |
| *C12orf57* | 0.006 | 6.438 |
| *ACOX2* | 0.006 | 3.914 |
| *RAD51B* | 0.006 | -3.888 |
| *P3H2* | 0.006 | 6.412 |
| *SLC34A2* | 0.007 | 5.464 |
| *PRELID1* | 0.007 | -3.957 |
| *MIR4697HG* | 0.007 | 5.486 |
| *SLFNL1* | 0.007 | 6.381 |
| *VCPKMT* | 0.007 | 5.681 |
| *CARNS1* | 0.007 | -5.299 |
| *RAD51D* | 0.007 | -4.984 |

| | | |
|---|---|---|
| *ITGA5* | 0.008 | 3.486 |
| *IMP3* | 0.008 | -5.644 |
| *GUCY1B1* | 0.008 | 6.040 |
| *SYNC* | 0.008 | 3.844 |
| *TLR2* | 0.008 | 5.146 |
| *MUC3A* | 0.009 | 4.191 |
| *CFAP61* | 0.009 | -5.627 |
| *NUDT15* | 0.009 | 4.080 |
| *LOC105371485* | 0.009 | -5.580 |
| *KCNN3* | 0.009 | 4.484 |
| *NUP37* | 0.010 | -6.027 |
| *UBL7-AS1* | 0.011 | 6.257 |
| *FAM184A* | 0.012 | -4.310 |
| *EIF4A1* | 0.012 | -5.455 |
| *ADAM20* | 0.012 | -5.073 |
| *STX18-AS1* | 0.013 | 4.411 |
| *ITGA4* | 0.013 | 4.204 |
| *LOC105374580* | 0.013 | 5.416 |
| *SLC19A1* | 0.013 | -3.405 |
| *OVCH2* | 0.014 | 6.113 |
| *LINC00847* | 0.014 | -5.371 |
| *MRPS23* | 0.014 | 2.894 |
| *RAPGEF3* | 0.015 | -3.509 |
| *CHCHD5* | 0.015 | -5.500 |
| *LRGUK* | 0.015 | -4.259 |
| *NAV3* | 0.016 | 3.105 |
| *AMN1* | 0.016 | -5.358 |
| *FIGNL1* | 0.019 | 3.754 |
| *FAM98B* | 0.019 | 3.018 |
| *TRIM65* | 0.020 | -4.667 |
| *DBF4B* | 0.020 | 3.371 |
| *LINC01535* | 0.020 | -4.520 |
| *FANCC* | 0.020 | 2.817 |
| *SPARCL1* | 0.020 | 3.049 |
| *ARL6IP6* | 0.020 | -4.673 |
| *ASIC3* | 0.020 | 4.473 |
| *CREB5* | 0.021 | 5.930 |
| *ETNK2* | 0.022 | -5.302 |
| *CD302* | 0.022 | -5.304 |
| *TUBG1* | 0.022 | 2.652 |
| *EGLN2* | 0.023 | -2.727 |
| *CYCS* | 0.023 | 3.001 |
| *MAD2L1* | 0.024 | 4.139 |
| *PAXIP1-AS1* | 0.024 | -4.606 |
| *ALG8* | 0.024 | 2.422 |
| *LRRC75B* | 0.025 | -5.165 |
| *NFS1* | 0.025 | -3.365 |
| *ASB8* | 0.027 | -4.029 |
| *TRMT44* | 0.027 | -2.701 |
| *SLIT3* | 0.028 | 3.826 |

| | | |
|---|---|---|
| *TGFB1I1* | 0.028 | 3.824 |
| *FAM3B* | 0.028 | 4.786 |
| *DNAAF4* | 0.028 | -5.141 |
| *BICD1* | 0.028 | 3.248 |
| *ILDR2* | 0.029 | 4.499 |
| *SNAPIN* | 0.029 | 3.865 |
| *DNAJC9* | 0.029 | -3.352 |
| *ANGPTL2* | 0.030 | -4.200 |
| *ZNF79* | 0.031 | -4.439 |
| *LOC101927787* | 0.032 | -4.530 |
| *RYR2* | 0.032 | 3.510 |
| *ALKBH2* | 0.032 | 3.463 |
| *CDK8* | 0.032 | 2.825 |
| *CA5BP1* | 0.033 | -4.149 |
| *NMNAT3* | 0.033 | -4.367 |
| *PLCG2* | 0.033 | 2.432 |
| *TTC31* | 0.033 | 2.377 |
| *ZNF324* | 0.034 | -3.089 |
| *MARK1* | 0.034 | 4.325 |
| *SLC25A11* | 0.035 | -3.017 |
| *ANO9* | 0.035 | -3.112 |
| *MAGI2* | 0.035 | 4.634 |
| *ZNF75D* | 0.035 | -2.504 |
| *TRIM17* | 0.036 | -4.980 |
| *TFCP2L1* | 0.038 | 3.603 |
| *SYT8* | 0.038 | -3.476 |
| *CYSTM1* | 0.039 | 3.133 |
| *WDR75* | 0.040 | 2.052 |
| *HCK* | 0.041 | 3.710 |
| *AIF1* | 0.042 | 4.875 |
| *TG* | 0.042 | -2.689 |
| *LMF2* | 0.042 | -3.196 |
| *TP73-AS1* | 0.042 | -2.345 |
| *MIR9-3HG* | 0.042 | -4.915 |
| *INTS7* | 0.042 | -2.765 |
| *PTPRB* | 0.042 | 3.566 |
| *GET4* | 0.042 | -2.946 |
| *KCNK3* | 0.042 | 4.386 |
| *TMEM220* | 0.042 | 4.324 |
| *DOCK2* | 0.042 | 2.552 |
| *AKAP12* | 0.043 | 2.878 |
| *FAM221B* | 0.043 | -4.488 |
| *VTCN1* | 0.045 | 3.018 |
| *MORC3* | 0.045 | 1.853 |
| *RGS1* | 0.045 | 2.784 |
| *ALKBH4* | 0.045 | 4.282 |
| *PINK1-AS* | 0.045 | 3.708 |
| *WWTR1* | 0.045 | 3.267 |
| *DNAH17* | 0.046 | 3.619 |
| *ZHX2* | 0.046 | 2.280 |

| | | |
|---|---|---|
| *IFI6* | 0.046 | 2.704 |
| *GID4* | 0.047 | -3.549 |
| *ZNF580* | 0.047 | -3.036 |
| *COL7A1* | 0.047 | -3.204 |
| *SLIT2* | 0.047 | 3.219 |
| *HMCN2* | 0.047 | 3.813 |
| *TOPORS* | 0.048 | 2.009 |
| *ALG1* | 0.048 | -3.476 |
| *FUT1* | 0.048 | -4.827 |
| *PPP5D1* | 0.048 | -4.171 |
| *ST20* | 0.049 | -4.546 |
| *SLPI* | 0.049 | -2.857 |
| *ZRSR2* | 0.049 | 2.013 |
| *LRP3* | 0.049 | -3.068 |
| *LINC00216* | 0.049 | 4.110 |

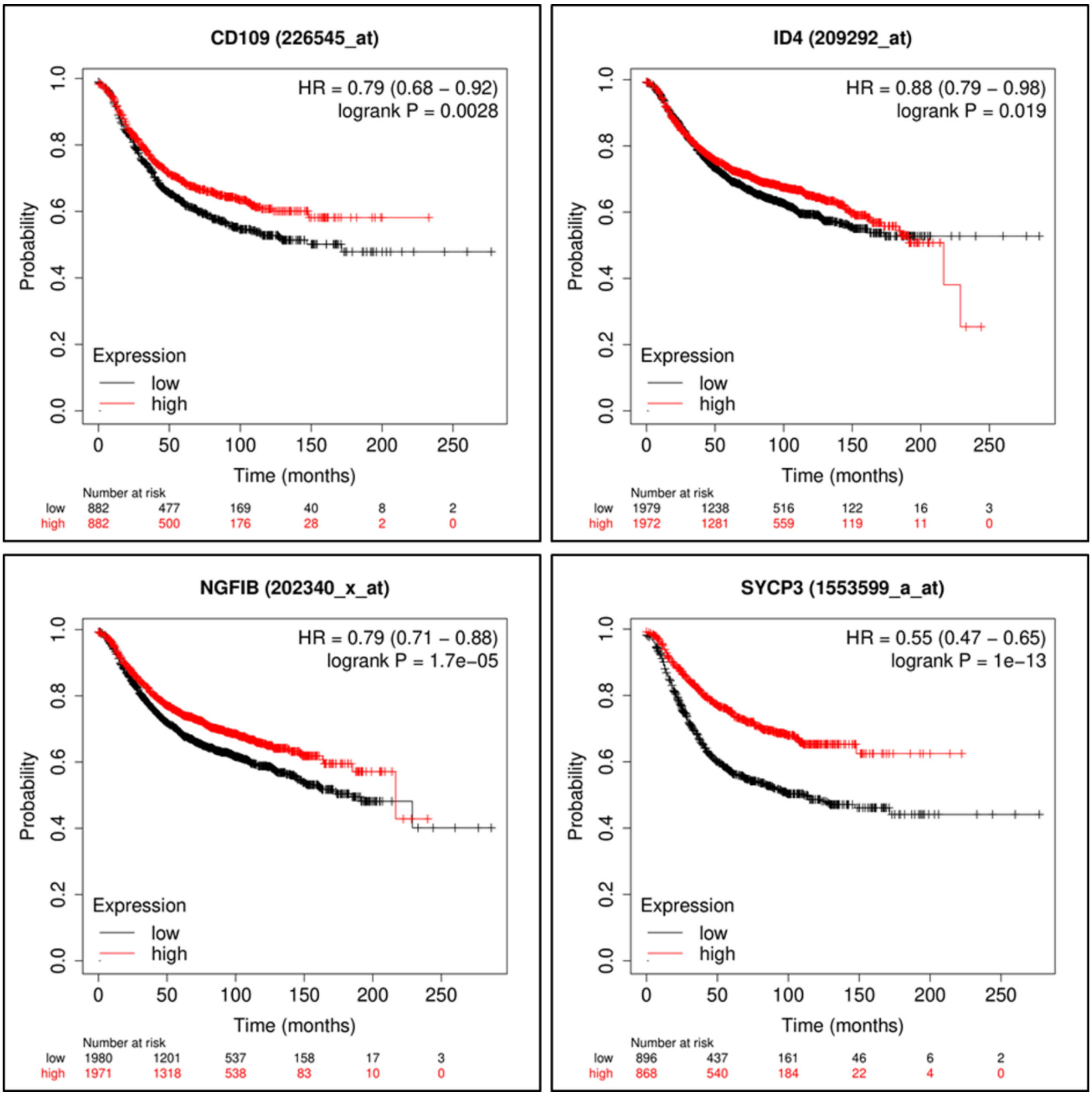


**Figure S7.** Association of DEGs (*CD109*, *ID4*, *NR4A1* and *SYCP3*) in EZR-negative cells with relapse-free survival of breast cancer patients according to Kaplan-Meyer Plotter database.

**Table S14.** KEGG enrichment analysis of up- and down-regulated genes in EZR-negative cells located in torpedo-like structures ($p < 0.05$)

| Term | P-value | Adjusted P-value | Genes |
|---|---|---|---|
| Homologous recombination | 0.000 | 0.102 | *RAD51B; RAD51D; EME1; SYCP3* |
| Fc gamma R-mediated phagocytosis | 0.006 | 0.989 | *HCK; PLCG2; DOCK2; FCGR2B* |
| Cortisol synthesis and secretion | 0.016 | 1.000 | *NR4A1; KCNK3; CREB5* |
| Arrhythmogenic right ventricular cardioı | 0.021 | 1.000 | *RYR2; ITGA4; ITGA5* |
| beta-Alanine metabolism | 0.026 | 1.000 | *EHHADH; CARNS1* |
| Hypertrophic cardiomyopathy (HCM) | 0.032 | 1.000 | *RYR2; ITGA4; ITGA5* |
| Insulin secretion | 0.033 | 1.000 | *RYR2; KCNN3; CREB5* |
| Dilated cardiomyopathy (DCM) | 0.038 | 1.000 | *RYR2; ITGA4; ITGA5* |
| Aldosterone synthesis and secretion | 0.045 | 1.000 | *NR4A1; KCNK3; CREB5* |

A total of 98 upregulated and 64 downregulated genes have been analyzed

**Table S15.** GO enrichment analysis of up- and down-regulated genes in EZR-negative cells located in torpedo-like structures ($p < 0.05$)

| Term | P-value | Adjusted P-value | Genes |
|---|---|---|---|
| regulation of phagocytosis (GO:0050764) | 0.000 | 1.000 | *HCK; FCGR2B; DOCK2; TLR2* |
| peptidyl-proline hydroxylation (GO:0019511) | 0.001 | 1.000 | *EGLN2; P3H2* |
| regulation of chemokine-mediated signaling pathway (GO:0070099) | 0.001 | 1.000 | *SLIT3; SLIT2* |
| strand invasion (GO:0042148) | 0.001 | 1.000 | *RAD51B; RAD51D* |
| neuron projection extension involved in neuron projection guidance (GO:1902284) | 0.002 | 1.000 | *SLIT3; SLIT2* |
| DNA recombinase assembly (GO:0000730) | 0.002 | 1.000 | *RAD51B; RAD51D* |
| glomerular visceral epithelial cell development (GO:0072015) | 0.002 | 1.000 | *JAG1; MAGI2* |
| glomerular epithelial cell development (GO:0072310) | 0.002 | 1.000 | *JAG1; MAGI2* |
| DNA repair complex assembly (GO:0090735) | 0.002 | 1.000 | *RAD51B; RAD51D* |
| axon extension involved in axon guidance (GO:0048846) | 0.002 | 0.902 | *SLIT3; SLIT2* |
| double-strand break repair via synthesis-dependent strand annealing (GO:0045003) | 0.002 | 0.820 | *RAD51B; RAD51D* |
| endodermal cell differentiation (GO:0035987) | 0.002 | 0.926 | *ITGA4; COL7A1; ITGA5* |
| negative regulation of phagocytosis (GO:0050765) | 0.002 | 0.888 | *FCGR2B; TLR2* |
| central nervous system projection neuron axonogenesis (GO:0021952) | 0.003 | 1.000 | *C12ORF57; SLIT2* |
| endoderm formation (GO:0001706) | 0.003 | 1.000 | *ITGA4; COL7A1; ITGA5* |
| negative regulation of chemotaxis (GO:0050922) | 0.003 | 1.000 | *SLIT2; AIF1* |
| response to ionizing radiation (GO:0010212) | 0.004 | 1.000 | *RAD51B; RAD51D; FIGNL1; INTS7* |
| positive regulation of mitotic cell cycle phase transition (GO:1901992) | 0.004 | 1.000 | *RAD51B; DBF4B; AIF1* |
| glomerular visceral epithelial cell differentiation (GO:0072112) | 0.005 | 1.000 | *JAG1; MAGI2* |
| inorganic anion transport (GO:0015698) | 0.005 | 1.000 | *SLC34A2; SLC26A8; ANO9* |

| | | | |
|---|---|---|---|
| oligosaccharide-lipid intermediate biosynthetic process (GO:0006490) | 0.006 | 1.000 | *ALG8; ALG1* |
| negative regulation of smooth muscle cell migration (GO:0014912) | 0.006 | 1.000 | *SLIT2; AIF1* |
| cell migration involved in sprouting angiogenesis (GO:0002042) | 0.006 | 1.000 | *NR4A1; SLIT2* |
| smooth muscle contraction (GO:0006939) | 0.006 | 1.000 | *HMCN2; MYH11* |
| fatty acid beta-oxidation using acyl-CoA oxidase (GO:0033540) | 0.006 | 1.000 | *ACOX2; EHHADH* |
| maintenance of protein location in nucleus (GO:0051457) | 0.006 | 1.000 | *MORC3; TOPORS* |
| positive regulation of G2/M transition of mitotic cell cycle (GO:0010971) | 0.007 | 1.000 | *RAD51B; DBF4B* |
| cilium movement (GO:0003341) | 0.007 | 1.000 | *CFAP61; DNAH17; DNAAF4* |
| negative regulation of cell motility (GO:2000146) | 0.008 | 1.000 | *JAG1; NAV3; MAGI2; SLIT2* |
| positive regulation of cell cycle G2/M phase transition (GO:1902751) | 0.008 | 1.000 | *RAD51B; DBF4B* |
| mitotic recombination (GO:0006312) | 0.009 | 1.000 | *RAD51B; RAD51D* |
| dolichol-linked oligosaccharide biosynthetic process (GO:0006488) | 0.009 | 1.000 | *ALG8; ALG1* |
| maintenance of protein localization in organelle (GO:0072595) | 0.009 | 1.000 | *MORC3; TOPORS* |
| blood vessel endothelial cell migration (GO:0043534) | 0.009 | 1.000 | *NR4A1; SLIT2* |
| negative regulation of lymphocyte activation (GO:0051250) | 0.010 | 1.000 | *VTCN1; FCGR2B* |
| cellular respiration (GO:0045333) | 0.011 | 1.000 | *CHCHD5; NR4A3; CYCS* |
| axon extension (GO:0048675) | 0.011 | 1.000 | *SLIT3; SLIT2* |
| regulation of monocyte chemotaxis (GO:0090025) | 0.014 | 1.000 | *SLIT2; AIF1* |
| cellular response to catecholamine stimulus (GO:0071870) | 0.015 | 1.000 | *RYR2; NR4A3* |
| regulation of proteolysis involved in cellular protein catabolic process (GO:1903050) | 0.015 | 1.000 | *DNAAF4; NUDT15* |
| positive regulation of endocytosis (GO:0045807) | 0.015 | 1.000 | *DOCK2; FCGR2B; BICD1* |
| response to glucocorticoid (GO:0051384) | 0.016 | 1.000 | *SLIT3; SLIT2* |

| | | | |
|---|---|---|---|
| receptor clustering (GO:0043113) | 0.016 | 1.000 | *ITGA4; MAGI2* |
| negative regulation of sequestering of calcium ion (GO:0051283) | 0.016 | 1.000 | *RYR2; PLCG2* |
| negative regulation of cell migration (GO:0030336) | 0.017 | 1.000 | *JAG1; NAV3; MAGI2; SLIT2* |
| heterotypic cell-cell adhesion (GO:0034113) | 0.017 | 1.000 | *ITGA4; ITGA5* |
| strand displacement (GO:0000732) | 0.019 | 1.000 | *RAD51B; RAD51D* |
| response to ketone (GO:1901654) | 0.019 | 1.000 | *SLIT3; SLIT2* |
| negative chemotaxis (GO:0050919) | 0.019 | 1.000 | *SLIT3; SLIT2* |
| release of sequestered calcium ion into cytosol (GO:0051209) | 0.020 | 1.000 | *RYR2; PLCG2* |
| negative regulation of endocytosis (GO:0045806) | 0.021 | 1.000 | *FCGR2B; TLR2* |
| regulation of defense response to virus by virus (GO:0050690) | 0.021 | 1.000 | *HCK; DOCK2* |
| regulation of striated muscle contraction (GO:0006942) | 0.023 | 1.000 | *C12ORF57; RYR2* |
| dicarboxylic acid transport (GO:0006835) | 0.023 | 1.000 | *SLC26A8; SLC19A1* |
| protein alkylation (GO:0008213) | 0.023 | 1.000 | *VCPKMT; FAM98B* |
| lysosome localization (GO:0032418) | 0.023 | 1.000 | *SNAPIN; NR4A3* |
| negative regulation of lymphocyte proliferation (GO:0050672) | 0.023 | 1.000 | *VTCN1; FCGR2B* |
| Fc receptor mediated stimulatory signaling pathway (GO:0002431) | 0.024 | 1.000 | *HCK; NR4A3; PLCG2; FCGR2B* |
| regulation of actin cytoskeleton reorganization (GO:2000249) | 0.024 | 1.000 | *HCK; RAPGEF3* |
| regulation of leukocyte chemotaxis (GO:0002688) | 0.024 | 1.000 | *ZNF580; SLIT2* |
| negative regulation of transmembrane receptor protein serine/threonine kinase signaling pathway (GO:0090101) | 0.025 | 1.000 | *TGFB1I1; CD109; MAGI2* |
| cellular response to hormone stimulus (GO:0032870) | 0.027 | 1.000 | *NR4A3; SLIT3; SLIT2* |
| ion transport (GO:0006811) | 0.031 | 1.000 | *RYR2; SCNN1D; SLC26A8; ANO9; ASIC3; KCNK3* |
| epithelial tube morphogenesis (GO:0060562) | 0.031 | 1.000 | *RYR2; SLIT2* |

| | | | |
|---|---|---|---|
| regulation of interleukin-8 production (GO:0032677) | 0.031 | 1.000 | *ZNF580; TLR2* |
| negative regulation of T cell activation (GO:0050868) | 0.031 | 1.000 | *VTCN1; FCGR2B* |
| regulation of microtubule polymerization (GO:0031113) | 0.033 | 1.000 | *NAV3; MAP2* |
| positive regulation of ATPase activity (GO:0032781) | 0.033 | 1.000 | *RYR2; DNAJC9* |
| ion transmembrane transport (GO:0034220) | 0.033 | 1.000 | *RYR2; SCNN1D; ANO9; ASIC3; KCNK3* |
| calcium ion transmembrane import into cytosol (GO:0097553) | 0.034 | 1.000 | *RYR2; PLCG2* |
| vesicle-mediated transport in synapse (GO:0099003) | 0.034 | 1.000 | *SNAPIN; SYT8* |
| positive regulation of smooth muscle cell proliferation (GO:0048661) | 0.036 | 1.000 | *NR4A3; AIF1* |
| organic cyclic compound biosynthetic process (GO:1901362) | 0.036 | 1.000 | *NFS1; TFCP2L1* |
| regulation of cell proliferation (GO:0042127) | 0.039 | 1.000 | *WWTR1; HCK; JAG1; NR4A3; TGFB1I1; MAGI2; P3H2; FAM98B; DBF4B; CGRRF1; SHCBP1* |
| regulation of proteasomal protein catabolic process (GO:0061136) | 0.040 | 1.000 | *DNAAF4; NUDT15* |
| positive regulation of phagocytosis (GO:0050766) | 0.040 | 1.000 | *FCGR2B; DOCK2* |
| DNA biosynthetic process (GO:0071897) | 0.040 | 1.000 | *RAD51B; RAD51D* |
| transcription initiation from RNA polymerase II promoter (GO:0006367) | 0.041 | 1.000 | *WWTR1; NR4A1; CDK8; NR4A3* |
| negative regulation of cytokine-mediated signaling pathway (GO:0001960) | 0.042 | 1.000 | *SLIT3; SLIT2* |
| neuron migration (GO:0001764) | 0.042 | 1.000 | *DNAAF4; MARK1* |
| positive regulation of epithelial to mesenchymal transition (GO:0010718) | 0.042 | 1.000 | *WWTR1; TGFB1I1* |
| regulation of ATPase activity (GO:0043462) | 0.042 | 1.000 | *VCPKMT; DNAJC9* |
| bicarbonate transport (GO:0015701) | 0.044 | 1.000 | *CA5B; SLC26A8* |

| | | | |
|---|---|---|---|
| positive regulation of receptor-mediated endocytosis (GO:0048260) | 0.044 | 1.000 | *MAGI2; BICD1* |
| negative regulation of apoptotic process (GO:0043066) | 0.044 | 1.000 | *HCK; PRELID1; FIGNL1; IFI6; VTCN1; ITGA5; TOPORS; MAD2L1* |
| cell morphogenesis involved in neuron differentiation (GO:0048667) | 0.044 | 1.000 | *MAP2; SLIT3; SLIT2* |
| protein localization to organelle (GO:0033365) | 0.044 | 1.000 | *BICD1; TOPORS; GET4* |
| cellular response to radiation (GO:0071478) | 0.045 | 1.000 | *FIGNL1; INTS7* |
| cellular response to lipoteichoic acid (GO:0071223) | 0.048 | 1.000 | *TLR2* |
| negative regulation of membrane potential (GO:0045837) | 0.048 | 1.000 | *PRELID1* |
| macropinocytosis (GO:0044351) | 0.048 | 1.000 | *DOCK2* |
| release of sequestered calcium ion into cytosol by sarcoplasmic reticulum (GO:0014808) | 0.048 | 1.000 | *RYR2* |
| response to heparin (GO:0071503) | 0.048 | 1.000 | *SLIT2* |
| striated muscle hypertrophy (GO:0014897) | 0.048 | 1.000 | *RYR2* |
| regulation of nuclear cell cycle DNA replication (GO:0033262) | 0.048 | 1.000 | *DBF4B* |
| negative regulation of acute inflammatory response (GO:0002674) | 0.048 | 1.000 | *FCGR2B* |
| cellular response to monoamine stimulus (GO:0071868) | 0.048 | 1.000 | *NR4A3* |
| response to lipoteichoic acid (GO:0070391) | 0.048 | 1.000 | *TLR2* |
| positive regulation of humoral immune response (GO:0002922) | 0.048 | 1.000 | *FCGR2B* |
| cellular response to bacterial lipopeptide (GO:0071221) | 0.048 | 1.000 | *TLR2* |
| response to DNA damage checkpoint signaling (GO:0072423) | 0.048 | 1.000 | *EME1* |
| positive regulation of phospholipid transport (GO:2001140) | 0.048 | 1.000 | *PRELID1* |
| negative regulation of phospholipase activity (GO:0010519) | 0.048 | 1.000 | *BICD1* |

| | | | |
|---|---|---|---|
| regulation of skeletal muscle contraction (GO:0014819) | 0.048 | 1.000 | *C12ORF57* |
| positive regulation of protein acetylation (GO:1901985) | 0.048 | 1.000 | *RAPGEF3* |
| retinal ganglion cell axon guidance (GO:0031290) | 0.048 | 1.000 | *SLIT2* |
| response to catecholamine (GO:0071869) | 0.048 | 1.000 | *NR4A3* |
| negative regulation of mononuclear cell migration (GO:0071676) | 0.048 | 1.000 | *SLIT2* |
| nucleoside phosphate catabolic process (GO:1901292) | 0.048 | 1.000 | *NUDT15* |
| negative regulation of leukocyte degranulation (GO:0043301) | 0.048 | 1.000 | *FCGR2B* |
| positive regulation of myeloid leukocyte cytokine production involved in immune response (GO:0061081) | 0.048 | 1.000 | *NR4A3* |
| positive regulation of protein localization to centrosome (GO:1904781) | 0.048 | 1.000 | *BICD1* |
| metal ion transport (GO:0030001) | 0.049 | 1.000 | *SCNN1D; SLC5A4; KCNK3* |
| sprouting angiogenesis (GO:0002040) | 0.049 | 1.000 | *NR4A1; SLIT2* |
| positive regulation of interleukin-8 production (GO:0032757) | 0.049 | 1.000 | *ZNF580; TLR2* |
| regulation of T cell activation (GO:0050863) | 0.049 | 1.000 | *PRELID1; VTCN1* |
| regulation of smooth muscle cell proliferation (GO:0048660) | 0.049 | 1.000 | *NR4A3; AIF1* |

A total of 98 upregulated and 64 downregulated genes have been analyzed

**Table S16.** Breast cancer patients with distant metastasis and positive expression of KIF14 and Mieap and negative expression of EZR at the tips of torpedo-like structures

| Patients | KIF14 expression | Mieap expression | EZR loss |
|---|---|---|---|
| 1 | No | No | No |
| 2 | Yes | Yes | Yes |
| 3 | Yes | Yes | Yes |
| 4 | No | Yes | Yes |
| 5 | No | No | No |
| 6 | Yes | Yes | No |
| 7 | Yes | Yes | No data |
| 8 | Yes | Yes | Yes |
| 9 | Yes | Yes | Yes |
| 10 | Yes | Yes | Yes |
| 11 | Yes | Yes | Yes |
| 12 | No data | No data | Yes |
| 13 | Yes | Yes | Yes |
| 14 | Yes | Yes | Yes |
| 15 | Yes | Yes | Yes |
| 16 | Yes | No data | No data |
| 17 | Yes | Yes | Yes |
| 18 | No data | No | Yes |
| 19 | Yes | Yes | Yes |

Orange color indicates the simultaneous expression of all three markers. Blue color indicates the simultaneous expression of any two markers.

**Table S17.** Expression of *MKI67, PLAU, PLAUR, MMP2, MMP9* and *MMP13* genes in KIF14-positive, Mieap-positive, EZR-negative cells located in torpedo-like structures

| Markers | KIF14-positive* | | Mieap-positive** | | EZR-negative*** | |
|---|---|---|---|---|---|---|
| | logFC | p-value | logFC | p-value | logFC | p-value |
| MKI67 | -0.425 | 0.802 | 0.470 | 0.677 | 1.736 | 0.126 |
| PLAU | - | - | 0.483 | 0.823 | - | - |
| PLAUR | 0.004 | 0.999 | 1.255 | 0.435 | -0.306 | 0.759 |
| MMP2 | 1.228 | 0.506 | 0.495 | 0.669 | 0.789 | 0.461 |
| MMP9 | -1.295 | 0.615 | 3.203 | 0.252 | -0.380 | 0.785 |
| MMP13 | -5.336 | 0.027 | -1.182 | 0.626 | -0.493 | 0.798 |

*, compared to KIF14-negative cells; **, compared to Mieap-negative cells; ***, compared to EZR-positive cells.

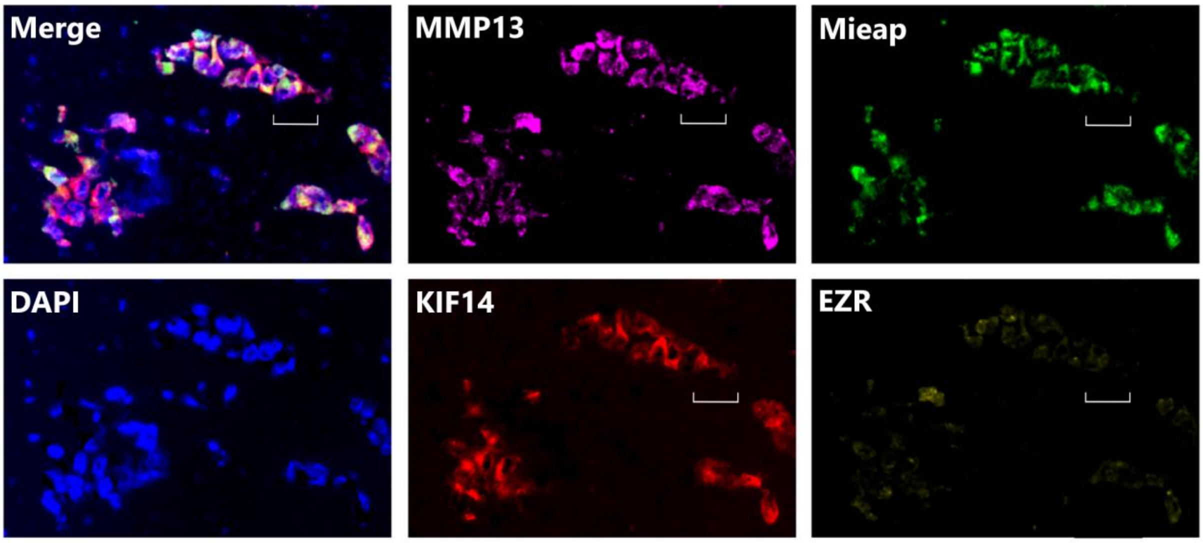


**Figure S8.** Expression of KIF14, Mieap, EZR and MMP13 in the torpedo-like structures.

**Table S18.** Clinicopathological parameters of breast cancer patients

| Parameter | | Number of cases, % |
|---|---|---|
| Age | ≤ 50 | 45 (53.5) |
| | > 50 | 35 (41.7) |
| | No data | 4 (4.8) |
| Menopause | Premenopausal | 44 (52.4) |
| | Postmenopausal | 35 (41.6) |
| | No data | 5 (6.0) |
| Tumor size | T1 | 32 (38.1) |
| | T2 | 38 (45.2) |
| | T3 | 3 (3.6) |
| | T4 | 7 (8.3) |
| | No data | 4 (4.8) |
| Grade | 1 | 4 (4.7) |
| | 2 | 58 (69.1) |
| | 3 | 9 (10.7) |
| | No data | 13 (15.5) |
| Lymph node metastases | N0 | 33 (39.2) |
| | N1 | 33 (39.2) |
| | N2 | 9 (10.7) |
| | N3 | 6 (7.4) |
| | No data | 3 (3.6) |
| Distant metastases | M0 | 45 (53.6) |
| | M1 | 39 (46.4) |
| Estrogen receptors | Yes | 53 (63.1) |
| | No | 26 (31.0) |
| | No data | 5 (5.9) |
| Progesterone receptors | Yes | 46 (54.8) |
| | No | 31 (36.9) |
| | No data | 7 (8.33) |
| HER2 | Yes | 39 (46.4) |
| | No | 39 (46.4) |
| | No data | 6 (7.2) |
| Molecular subtype | Luminal A | 14 (16.7) |
| | Luminal B | 39 (46.4) |
| | Triple-negative | 17 (20.2) |
| | HER2-positive | 9 (10.7) |
| | No data | 5 (6.0) |
| NAC | Yes | 34 (40.5) |
| | No | 50 (59.5) |

NAC, neoadjuvant chemotherapy

**Table S19.** Protocol of the assessment of immunohistochemical staining in breast tumors

| Parameter | Value |
|---|---|
| Diffuse staining within the tumor section | Yes, No;<br>Weak,<br>Moderate |
| Tumor cells with positive staining in comparison with background staining (regardless of morphological structures) | Up to 10%,<br>Up to 50% |
| Tumor cells with negative staining compared to background staining (regardless of morphological structures) | More than 50%<br>Totally |
| Tumor cells with negative staining at the tips (up to 3 rows) of: | |
| torpedo-like sprouts associated with solid structures | Yes, No;<br>Single, Many; |
| torpedo-like structures (separated from solid structures) | No structures |
| Negative staining in single tumor cells | Yes, No;<br>Single, Many;<br>No single tumor cells |
| Tumor cells with positive staining at the tips (up to 3 rows) of: | |
| torpedo-like sprouts associated with solid structures | Yes, No;<br>Single, Many; |
| torpedo-like structures (separated from solid structures) | No structures |
| Tumor cells with positive focal or diffuse staining in torpedo-like structures (except for the tips) | Yes, No |
| Positive staining in single tumor cells | Yes, No;<br>Single, Many;<br>No structures |
| Negative segmental staining at the periphery (up to 2 layers) of structures in the cells with | |
| solid<br>alveolar<br>trabecular<br>tubular | Yes, No;<br>Single, Many;<br>No structures |
| Negative segmental staining at the periphery (up to 2 layers) of structures in the cells with | |
| solid<br>alveolar<br>trabecular<br>tubular | Yes, No;<br>Single, Many;<br>No structures |
| Positive segmental staining at the periphery (up to 2 layers) of structures in the cells with | |
| solid<br>alveolar<br>trabecular<br>tubular | Yes, No;<br>Single, Many;<br>No structures |
| Positive segmental staining at the periphery (up to 2 layers) of structures in the cells with | |
| solid<br>alveolar<br>trabecular<br>tubular | Yes, No;<br>Single, Many;<br>No structures |
| Atypical positive staining with nonspecific localization in tumor cells | Yes, No |

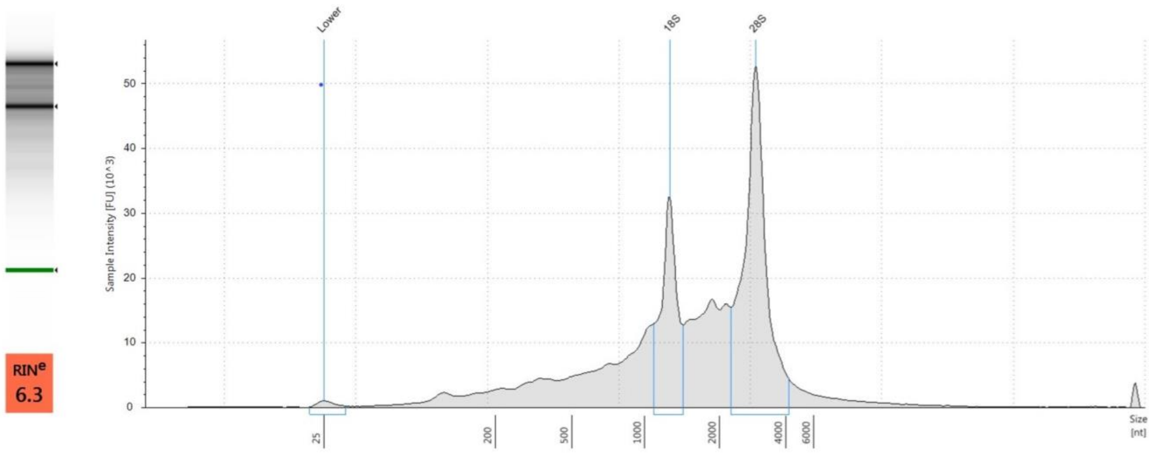


**Figure S9.** RNA integrity (RIN) of tumor tissue section following RNA-preserving immunolabeling.

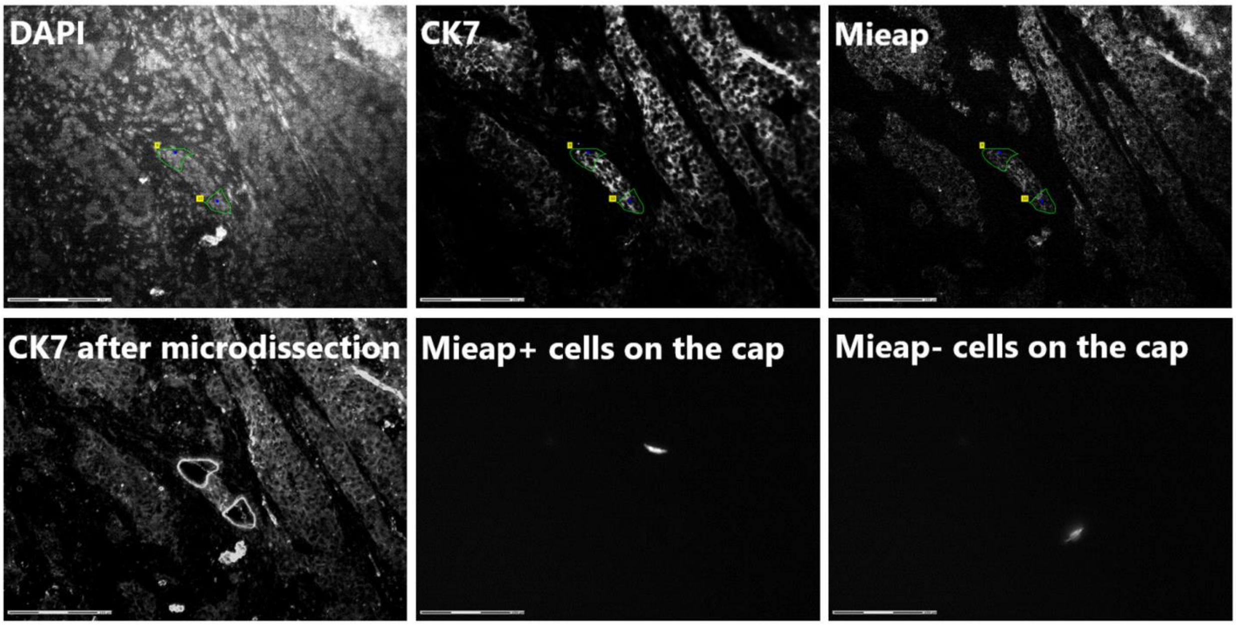


**Figure S10.** Fluorescence-guided laser microdissection on the example of CK7-positive Mieap-positive and Mieap-negative cells of the torpedo-like structure in breast tumor.
